# Supplementary material for: Chaperone-mediated autophagy regulates the metastatic state of mesenchymal tumors
Source: EMBO Mol Med. 2025 Mar 7;17(4):747–74. doi: 10.1038/s44321-025-00210-w (PMC11982252; doi:10.1038/s44321-025-00210-w)
Supplement: Supplementary file 1 — Appendix [file 44321_2025_210_MOESM1_ESM.pdf]

Appendix: Table of Contents

Appendix Tables 2

Appendix Table S1: Clinical information of patients providing lung cancer tissue samples and matching brain metastasis. 2

Appendix Table S2. Primers used for qRT-PCR. 3

Appendix Table S3. Antibodies used for WB, IF, and IHC. 4

Appendix Table S4. Statistical analysis of western blot densitometry from figure 7. 6

Appendix Tables

Appendix Table S1: Clinical information of patients providing lung cancer tissue samples and matching brain metastasis.

Patient	Age	Sex	Ethnicity
1	63	M	Caucasian
2	68	M	Caucasian
3	62	M	Caucasian
4	66	M	Caucasian
5	73	F	Caucasian
6	65	M	Caucasian

Appendix Table S2. Primers used for qRT-PCR.

Gene	Forward Primer (5'-3')	Reverse Primer (5'-3')
β-actin	GCAAGCAGGAGTATGACGAG	CAAATAAAGCCATGCCAATC
CDH1	CTCGACACCCGATTCAAAGT	CCAGGCGTAGACCAAGAAAT
ESD	TTTACTTCGGCCCGCTTCTT	TCCTGTCCAATTGCTGATTGC
HPRT	GATCAGTCAACGGGGGACATAAA	CTTGCGCTCATCTTAGGCTTTGT
LAMP-2A	ACTGTTTCAGTGTCTGGAGCAT	ATGGGCACAAGGAAGTTGTC
LAMP-2B	AGGGTTCAGCCTTTCAATGT	CTGAAAGACCAGCACCAACTA
LAMP-2C	TCAGTGTCTGGAGCATTTCAG	GGTCAGAGTCAGCAGAACATT
LAMP1	TGTGGACAAGTACAACGTGAG	CGTGTTGTCCTTCCTCTCATAG
SMAD4	TGCATTCCAGCCTCCCATTT	GCACACCTTTGCCTATGTGC
TGFβR2	ATGACATCTCGCTGTAATGC	GGATGCCCTGGTGGTTGA
VIM	GATTCACTCCCTCTGGTTGATAC	GTCATCGTGATGCTGAGAAGT

Appendix Table S3. Antibodies used for WB, IF, and IHC.

Target	Company	Catalog No.	Application	Dilution
β-actin	Santa Cruz	sc81178	WB	1:10000
E-Cadherin	Technology<br>Santa Cruz	sc71009	WB	1:1000
HK2	Thermo Fisher<br>Scientific	2H8L6	WB	1:1000
ID-1	BioCheck	BCH-1, clone<br>195-14	IHC	1:500
LAMP1	Santa Cruz	sc-20011	WB<br>IF	1:2000<br>1:300
LAMP-2A	Abcam	ab240018	WB<br>IHC<br>IF	1:2000<br>1:500<br>1:300
LAMP-2B	Abcam	ab18529	WB	1:2000
N-Cadherin	Technology<br>Santa Cruz	sc59987	WB	1:1000
p53	Santa Cruz	DO-1	WB	1:1000
Slug	Cell Signaling<br>Technology	C19G7	WB	1:1000
Smad2	Cell Signaling<br>Technology	D43B4	WB	1:1000
pSmad2	Cell Signaling<br>Technology	138D4	WB	1:1000
Smad3	Cell Signaling<br>Technology	C67H9	WB	1:2000
pSmad3	Cell Signaling<br>Technology	C25A9	WB	1:2000
Smad4	Cell Signaling<br>Technology	D3M6U	WB	1:2000
Snail	Cell Signaling<br>Technology	C15D3	WB	1:1000
TGFβR1	Abcam	Ab235178	WB	1:2000
TGFβR2	Abcam	ab184948	WB	1:1000
Twist	Santa Cruz	sc-81417	WB	1:1000
Vimentin	Atlas Antibodies Ab<br>Cell Signaling<br>Technology	HPA001762<br>D21H3	IHC<br>WB	1:1000<br>1:2000
Vinculin	Abcam	ab129002	WB	1:2000
Zeb1	Cell Signaling<br>Technology	D80D3	WB	1:1000
LDHA	Cell Signaling<br>Technology	3582	WB	1:1000
TOMM40	Santa Cruz	sc-11414	WB	1:1000
CTSD	Santa Cruz	sc-6486	WB	1:1000
HSC70	Proteintech	10654	WB	1:1000
LC3B	Sigma-Aldrich	L7543	WB	1:1000

Rabbit IgG (Alexa Fluor Plus 647)	Thermo Fisher Scientific	A32733	IF	1:500
Mouse IgG (Alexa Fluor Plus 488)	Thermo Fisher Scientific	A32723	IF	1:500
Rabbit IgG (Alexa Fluor 488)	Thermo Fisher Scientific	A32790	IF	1:500
Rabbit IgG (Alexa Fluor 594)	Invitrogen	A21207	IF	1:500
Rabbit IgG (HRP-conjugated)	Vector Laboratories	MP-7401	IHC	1:500

Appendix Table S4. Statistical analysis of western blot densitometry from figure 7.

Figure Panel	Cell line	Protein	Comparison	*P*-value
B	ES2	TGFβRII	CTRL-Tranilast	0.0037
			CTRL-CMA	0.0070
		Smad2	CTRL-Tranilast	0.0030
			CTRL-CMA	0.0192
		Smad3	CTRL-Tranilast	0.0008
			CTRL-CMA	0.0164
		Smad4	CTRL-Tranilast	0.0910
			CTRL-CMA	0.0037
		mP53	CTRL-Tranilast	0.0039
			CTRL-CMA	0.0051
C	FUOV1	TGFβRII	CTRL-CQ	0.0002
			CTRL-Tranilast	<0.0001
			Tranilast-Tranilast+CQ	0.0020
			CTRL-CMA	0.0031
			CMA-CMA+CQ	0.0187
	ES2		CTRL-CQ	0.0016
			CTRL-Tranilast	0.0119
			Tranilast-Tranilast+CQ	0.0298
			CTRL-CMA	0.0010
			CMA-CMA+CQ	0.0317
E	FUOV1	Snail	CTRL-CMA	0.0001
		Zeb1	CTRL-CMA	0.0006
		Slug	CTRL-CMA	0.0001
	ES2	Twist	CTRL-CMA	<0.0001
		Zeb1	CTRL-CMA	<0.0001
		Slug	CTRL-CMA	0.0633
F	OVPA8	Snail	CTRL-TGFβL	<0.0001
			TGFβL-TGFβL+CMA	0.0107
			CTRL-CMA	0.0212
G	OVPA8	LAMP-2A	NT-siLAMP-2A	0.0008
		TGFβRII	NT-siLAMP-2A	0.0019
H	HT1080	TGFβRII	WT-KO	0.0004
		TGFβRI	WT-KO	0.1982
		pSmad3	WT-KO	0.1715
		Smad2	WT-KO	0.0235
		Smad3	WT-KO	0.2684
		Smad4	WT-KO	0.0112

	A549	TGFβRII	WT-KO	0.4935
		TGFβRI	WT-KO	0.1284
		psmad2	WT-KO	0.3013
		pSmad3	WT-KO	0.1059
		Smad2	WT-KO	0.2913
		Smad3	WT-KO	0.0633
		Smad4	WT-KO	0.1187
I	HT1080 (tumor)	TGFβRII	WT-KO	0.0397
		TGFβRI	WT-KO	0.0162
		psmad2	WT-KO	0.1599
		Smad2	WT-KO	0.3088
		pSmad3	WT-KO	0.1377
		Smad3	WT-KO	0.0033
		Smad4	WT-KO	0.0075
J	HT1080	TGFβRII	WT-KO	<0.0001
			KO-KO RE	0.0027
