# Supplementary figures and images for: Chaperone-mediated autophagy regulates the metastatic state of mesenchymal tumors (part 1 of 2)
Source: EMBO Mol Med. 2025 Mar 7;17(4):747–74. doi: 10.1038/s44321-025-00210-w (PMC11982252; doi:10.1038/s44321-025-00210-w)

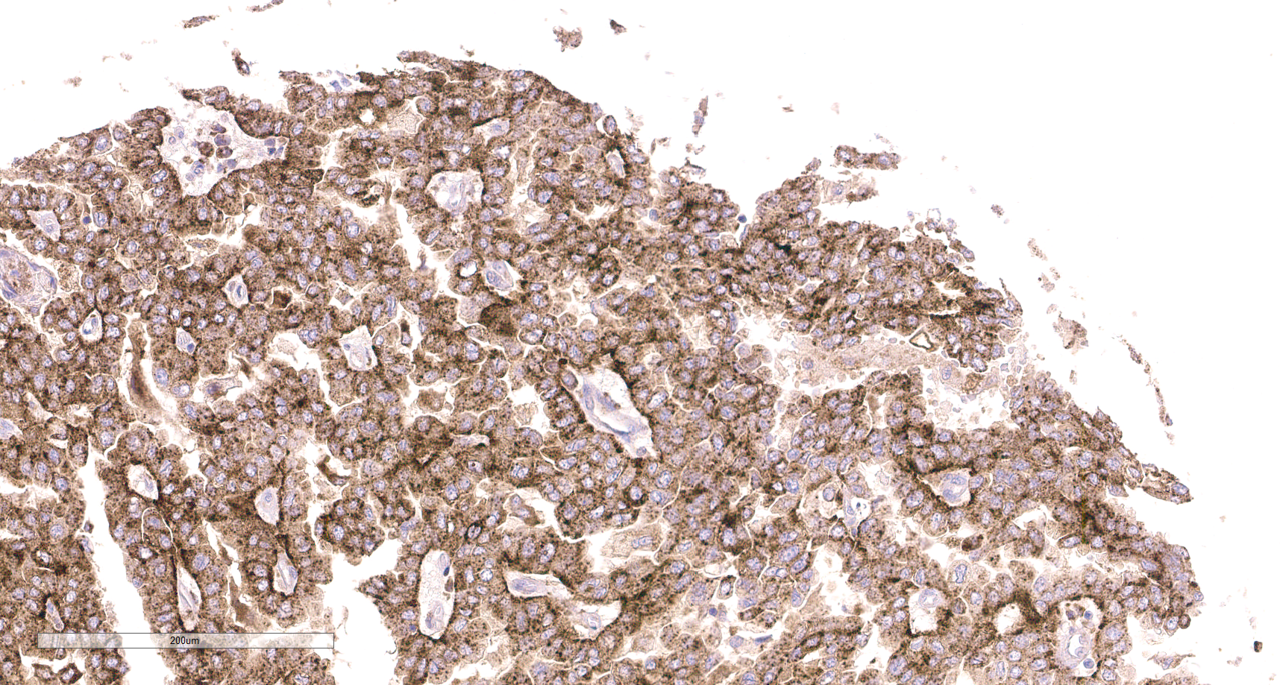

Supplement: Supplementary file 4 — Source data Fig. 2 [file 44321_2025_210_MOESM4_ESM.zip › Figure 2/2E/TMA_LAMP2A_tumor2.tif]

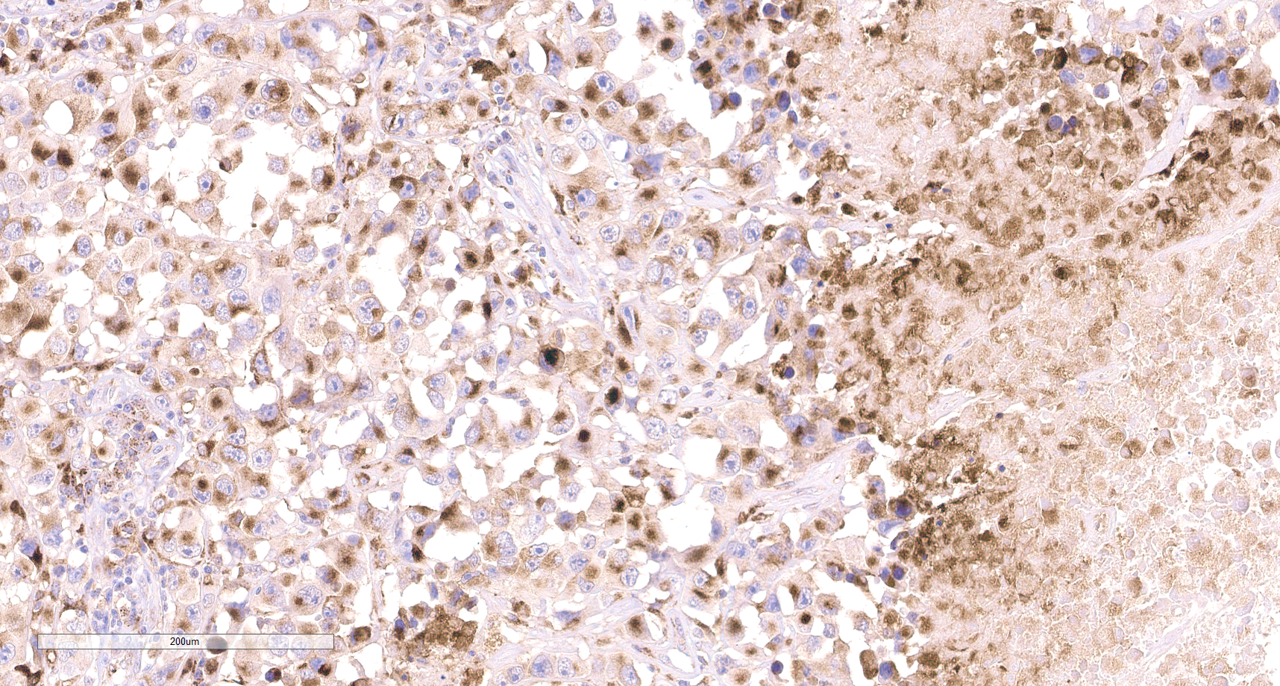

Supplement: Supplementary file 4 — Source data Fig. 2 [file 44321_2025_210_MOESM4_ESM.zip › Figure 2/2E/TMA_LAMP2A_tumor3.tif]

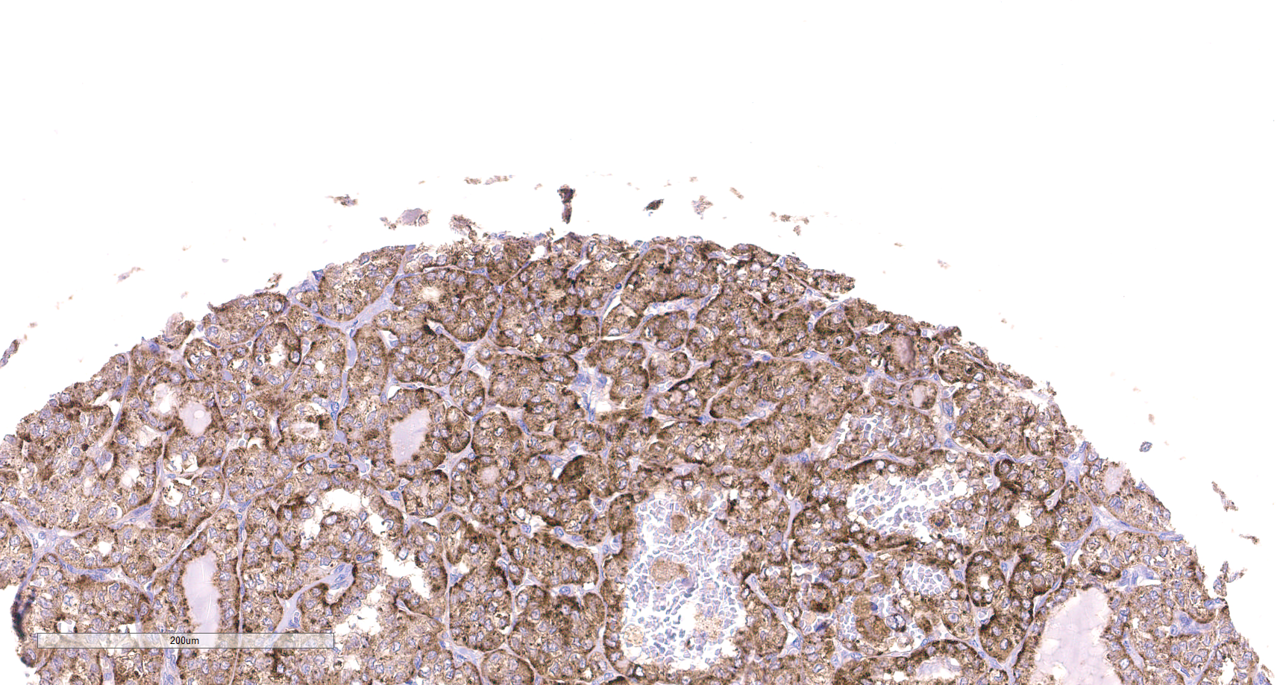

Supplement: Supplementary file 4 — Source data Fig. 2 [file 44321_2025_210_MOESM4_ESM.zip › Figure 2/2E/TMA_LAMP2A_tumor1.tif]

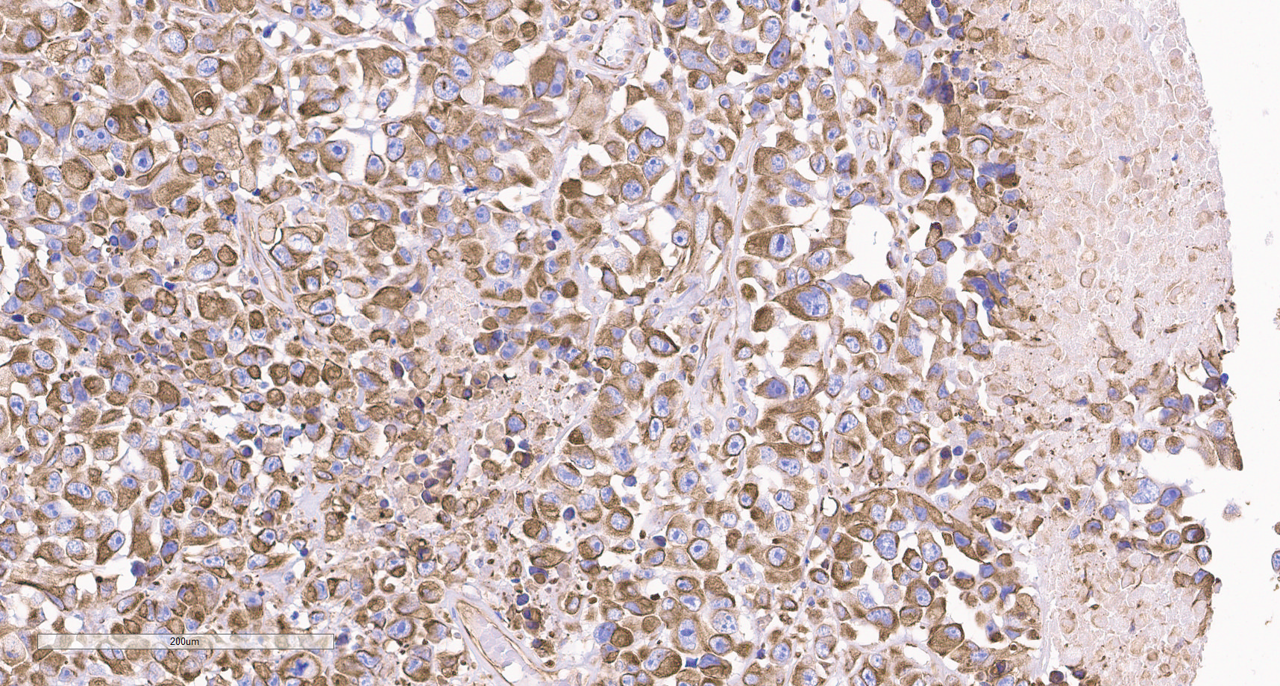

Supplement: Supplementary file 4 — Source data Fig. 2 [file 44321_2025_210_MOESM4_ESM.zip › Figure 2/2E/TMA_VIM_tumor4.tif]

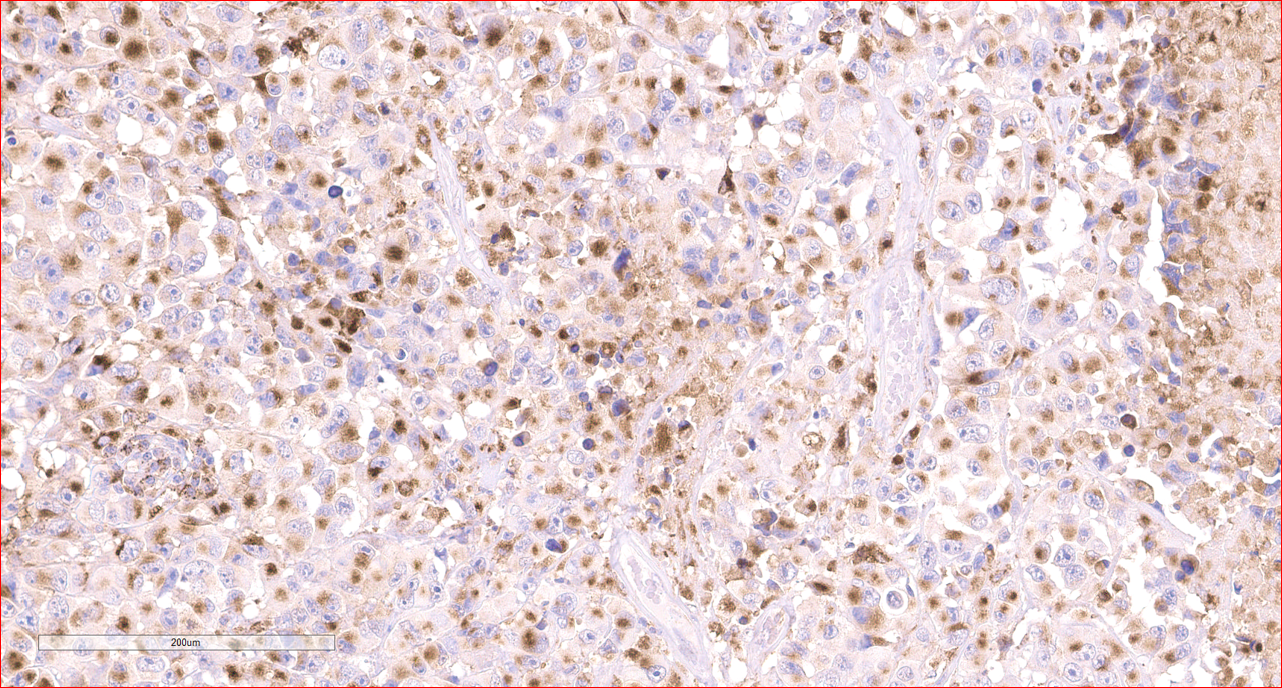

Supplement: Supplementary file 4 — Source data Fig. 2 [file 44321_2025_210_MOESM4_ESM.zip › Figure 2/2E/TMA_LAMP2A_tumor4.tif]

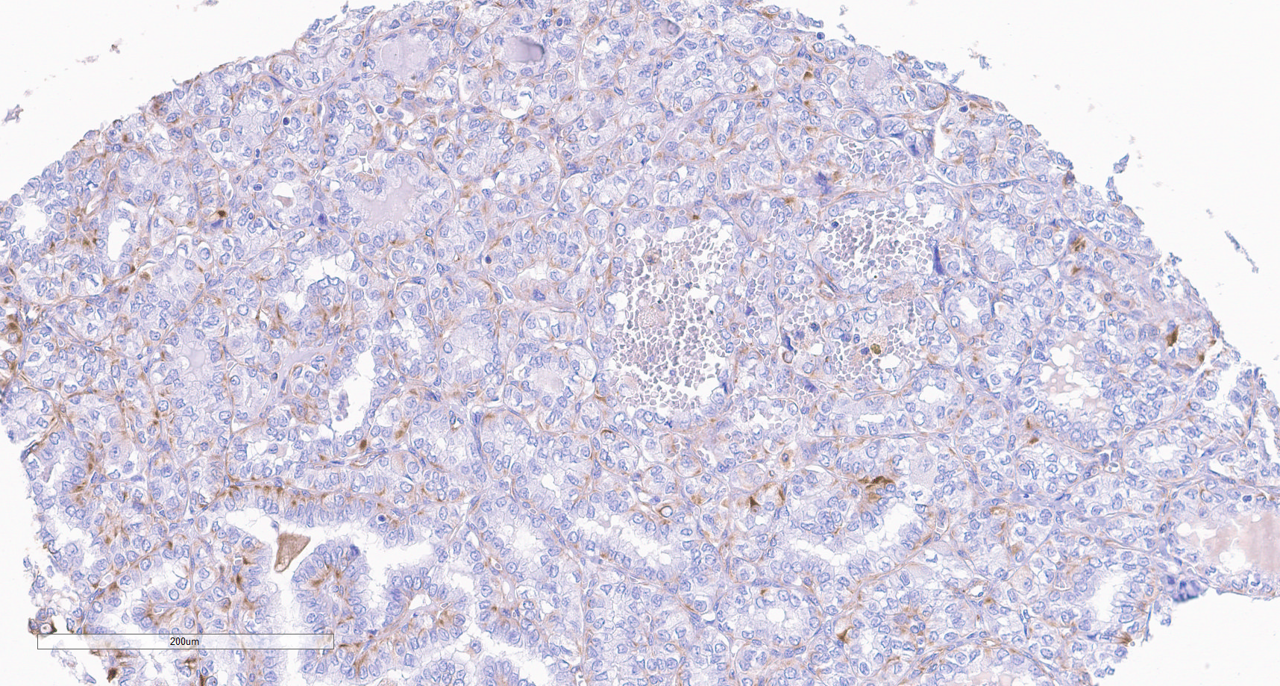

Supplement: Supplementary file 4 — Source data Fig. 2 [file 44321_2025_210_MOESM4_ESM.zip › Figure 2/2E/TMA_VIM_tumor1.tif]

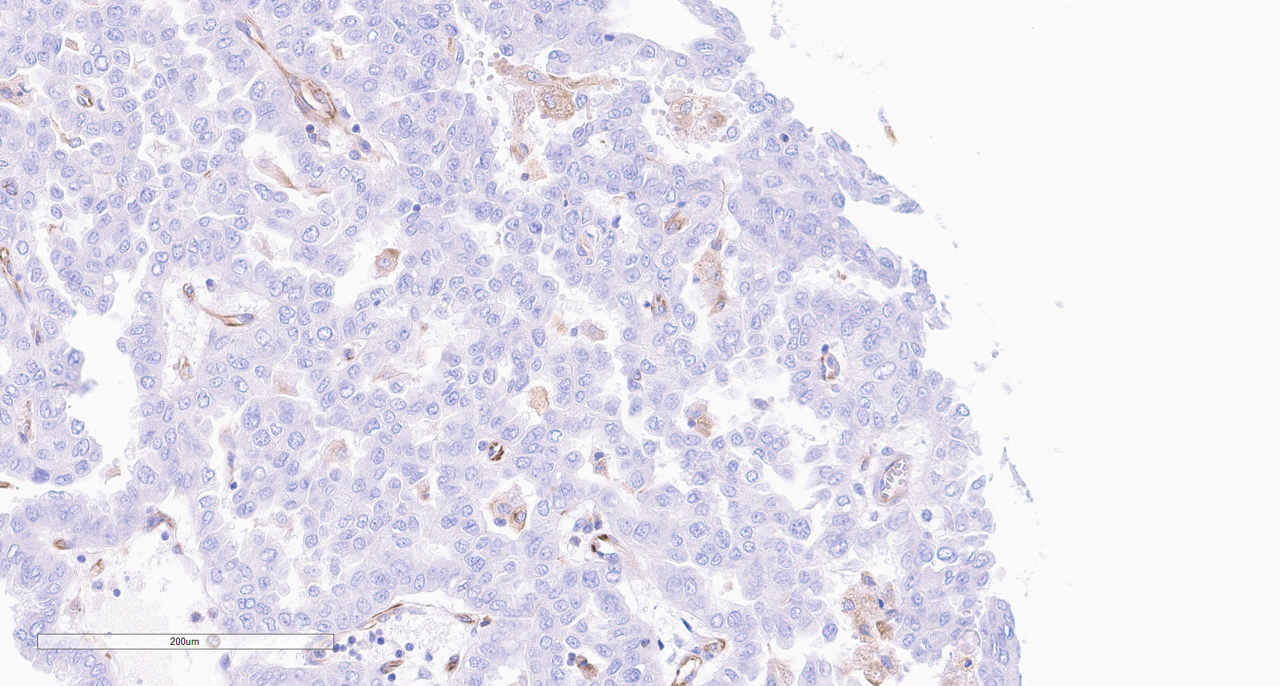

Supplement: Supplementary file 4 — Source data Fig. 2 [file 44321_2025_210_MOESM4_ESM.zip › Figure 2/2E/TMA_VIM_tumor2.tif]

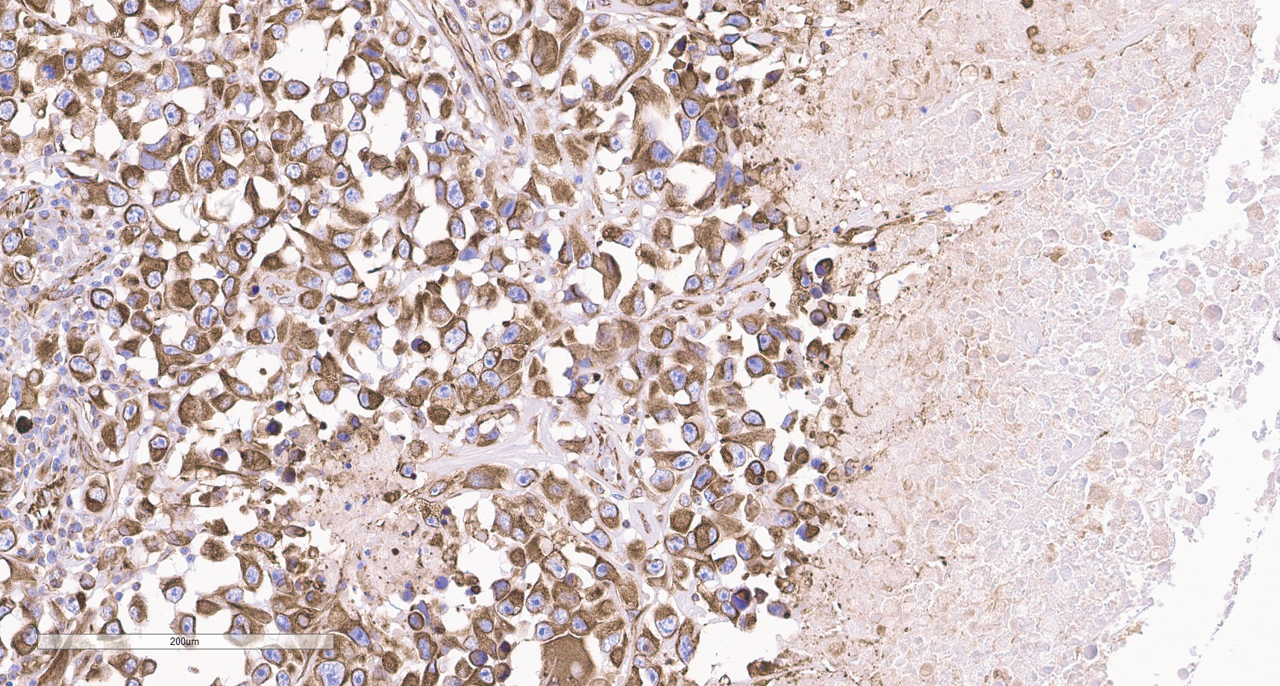

Supplement: Supplementary file 4 — Source data Fig. 2 [file 44321_2025_210_MOESM4_ESM.zip › Figure 2/2E/TMA_VIM_tumor3.tif]

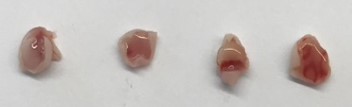

Supplement: Supplementary file 5 — Source data Fig. 3 [file 44321_2025_210_MOESM5_ESM.zip › Figure 3/3E/HT1080_tumors_WT_1.jpg]

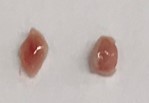

Supplement: Supplementary file 5 — Source data Fig. 3 [file 44321_2025_210_MOESM5_ESM.zip › Figure 3/3E/HT1080_tumors_WT_2.jpg]

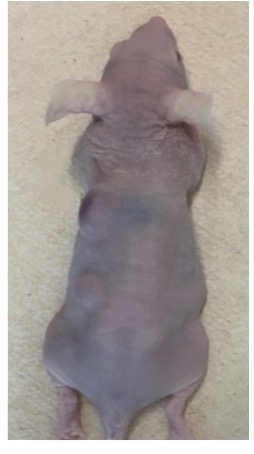

Supplement: Supplementary file 5 — Source data Fig. 3 [file 44321_2025_210_MOESM5_ESM.zip › Figure 3/3E/A549 mouse.jpg]

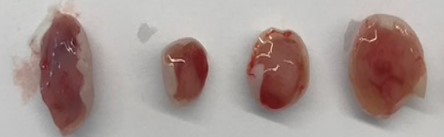

Supplement: Supplementary file 5 — Source data Fig. 3 [file 44321_2025_210_MOESM5_ESM.zip › Figure 3/3E/HT1080_tumors_KO_1.jpg]

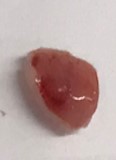

Supplement: Supplementary file 5 — Source data Fig. 3 [file 44321_2025_210_MOESM5_ESM.zip › Figure 3/3E/HT1080_tumors_KO_2.jpg]

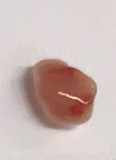

Supplement: Supplementary file 5 — Source data Fig. 3 [file 44321_2025_210_MOESM5_ESM.zip › Figure 3/3E/HT1080_tumors_KO_3.jpg]

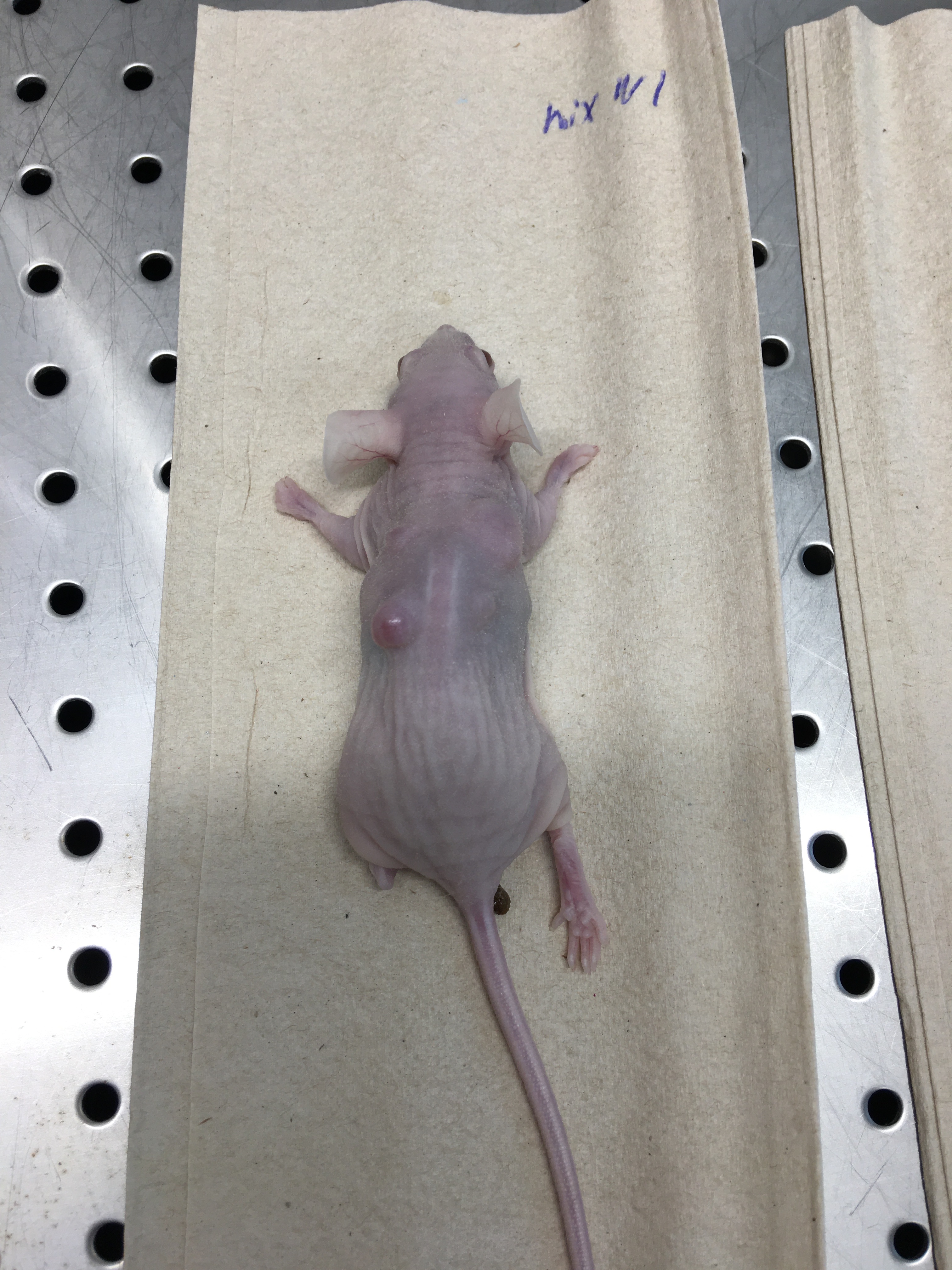

Supplement: Supplementary file 5 — Source data Fig. 3 [file 44321_2025_210_MOESM5_ESM.zip › Figure 3/3E/HT1080_mice_2.JPG]

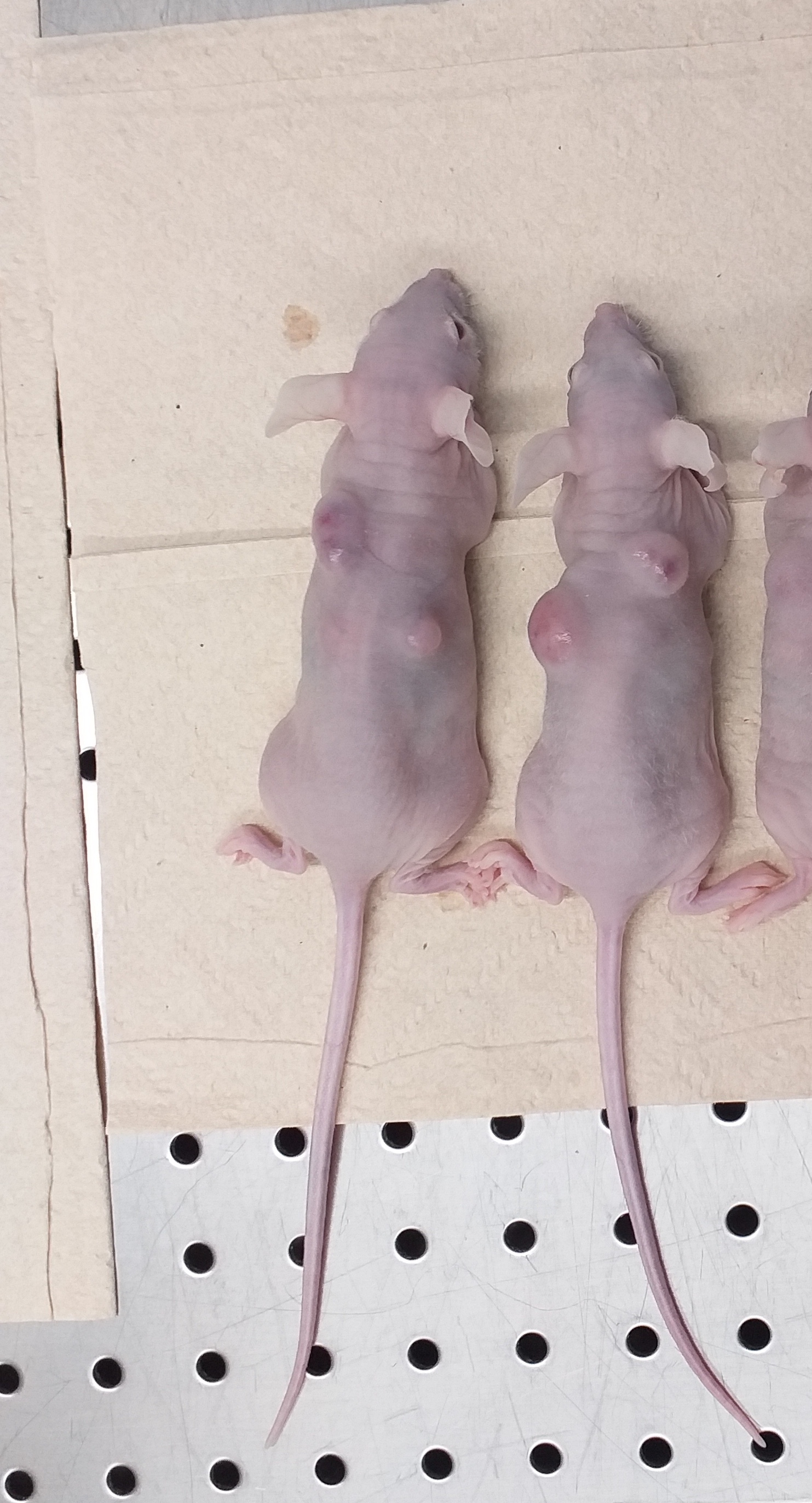

Supplement: Supplementary file 5 — Source data Fig. 3 [file 44321_2025_210_MOESM5_ESM.zip › Figure 3/3E/HT1080_mice_1.jpg]

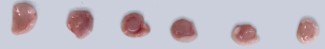

Supplement: Supplementary file 5 — Source data Fig. 3 [file 44321_2025_210_MOESM5_ESM.zip › Figure 3/3E/A549_tumors_2.jpg]

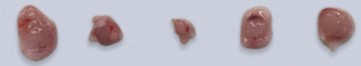

Supplement: Supplementary file 5 — Source data Fig. 3 [file 44321_2025_210_MOESM5_ESM.zip › Figure 3/3E/A549_tumors_1.jpg]

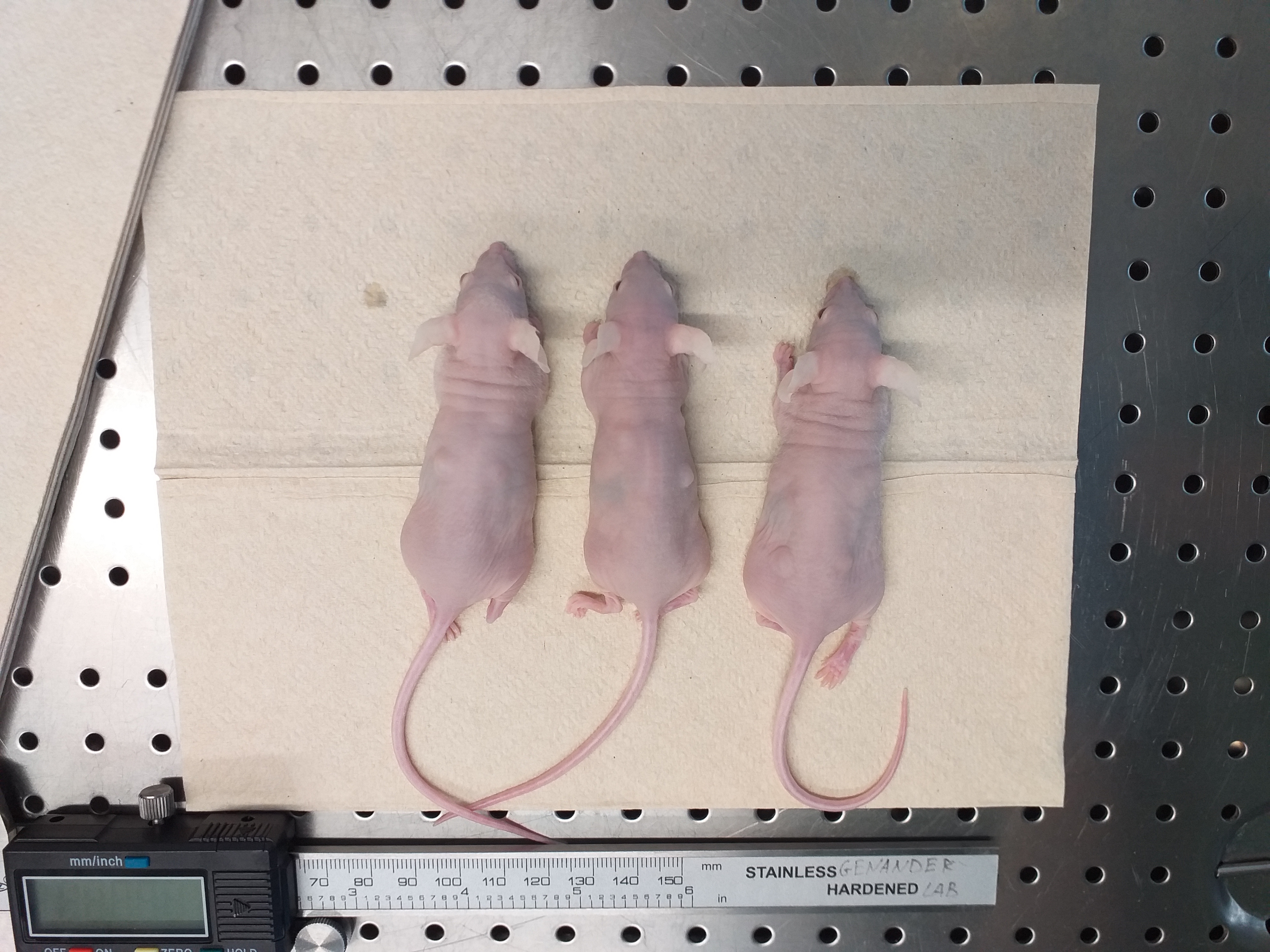

Supplement: Supplementary file 5 — Source data Fig. 3 [file 44321_2025_210_MOESM5_ESM.zip › Figure 3/3E/A549_mice.jpg]

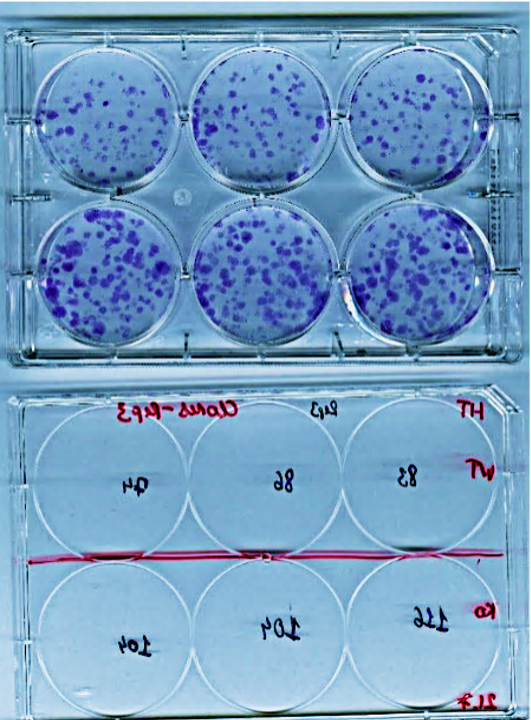

Supplement: Supplementary file 5 — Source data Fig. 3 [file 44321_2025_210_MOESM5_ESM.zip › Figure 3/3B/HT1080.png]

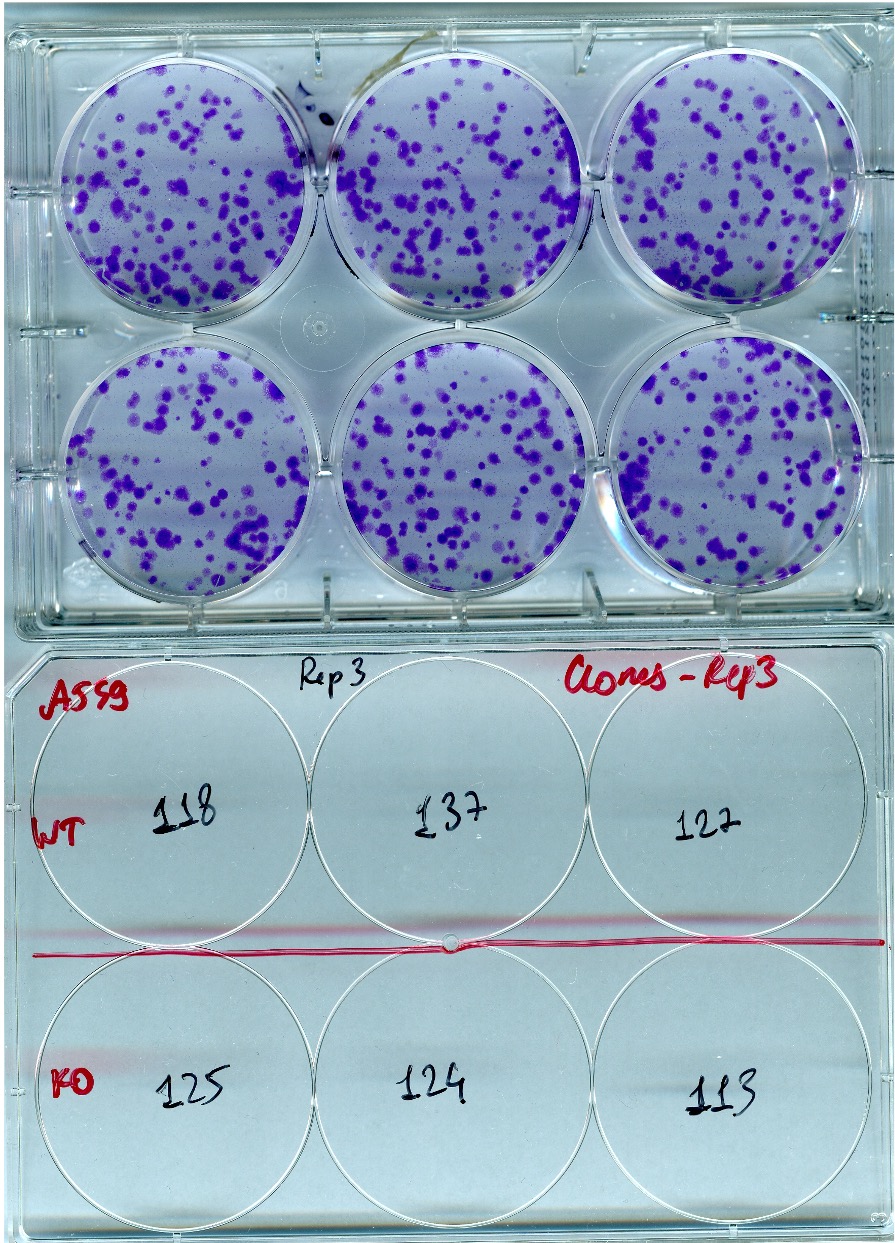

Supplement: Supplementary file 5 — Source data Fig. 3 [file 44321_2025_210_MOESM5_ESM.zip › Figure 3/3B/A549.jpg]

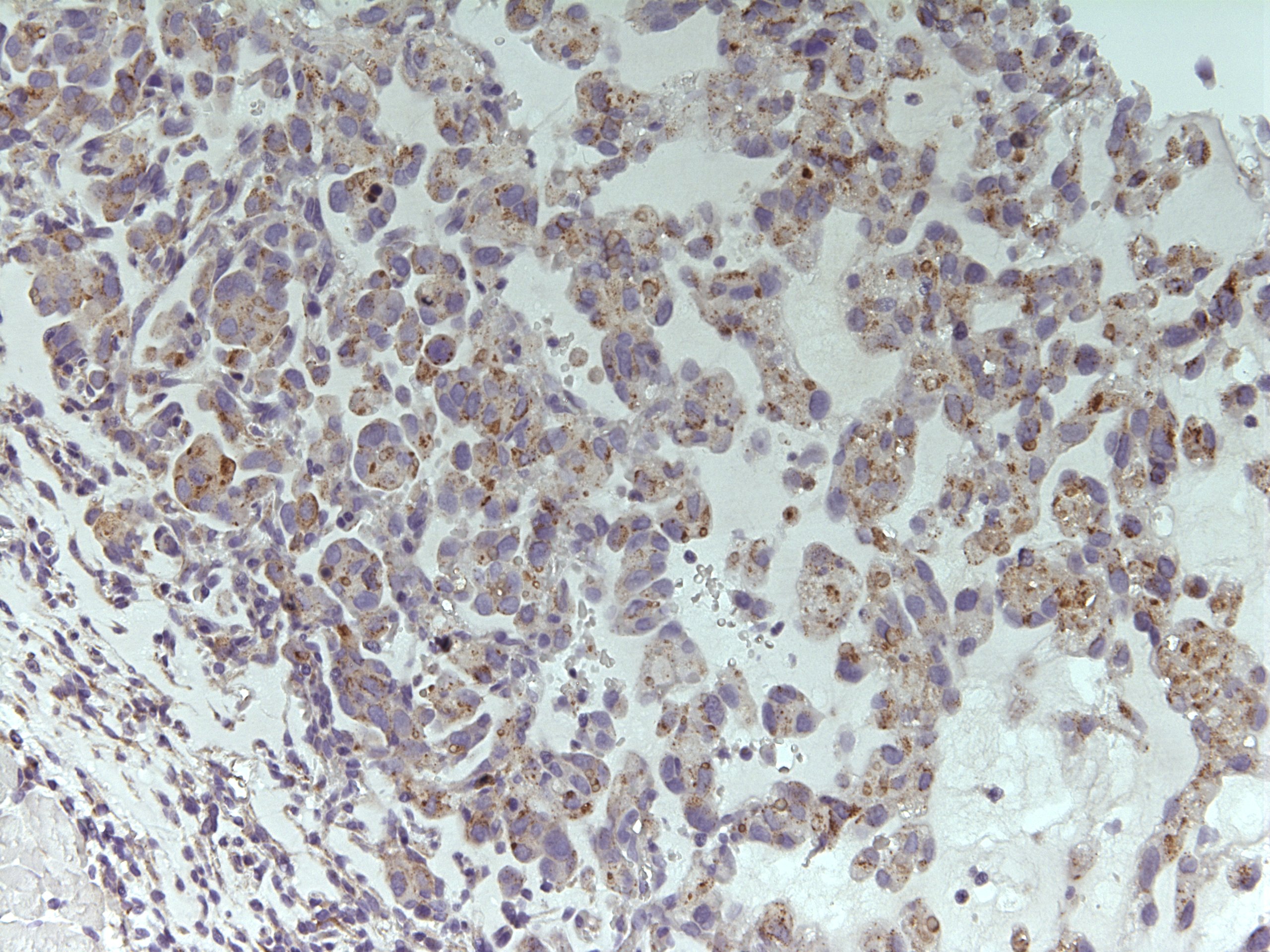

Supplement: Supplementary file 5 — Source data Fig. 3 [file 44321_2025_210_MOESM5_ESM.zip › Figure 3/3D/IHC_HT1080_WT_LAMP-2A.jpg]

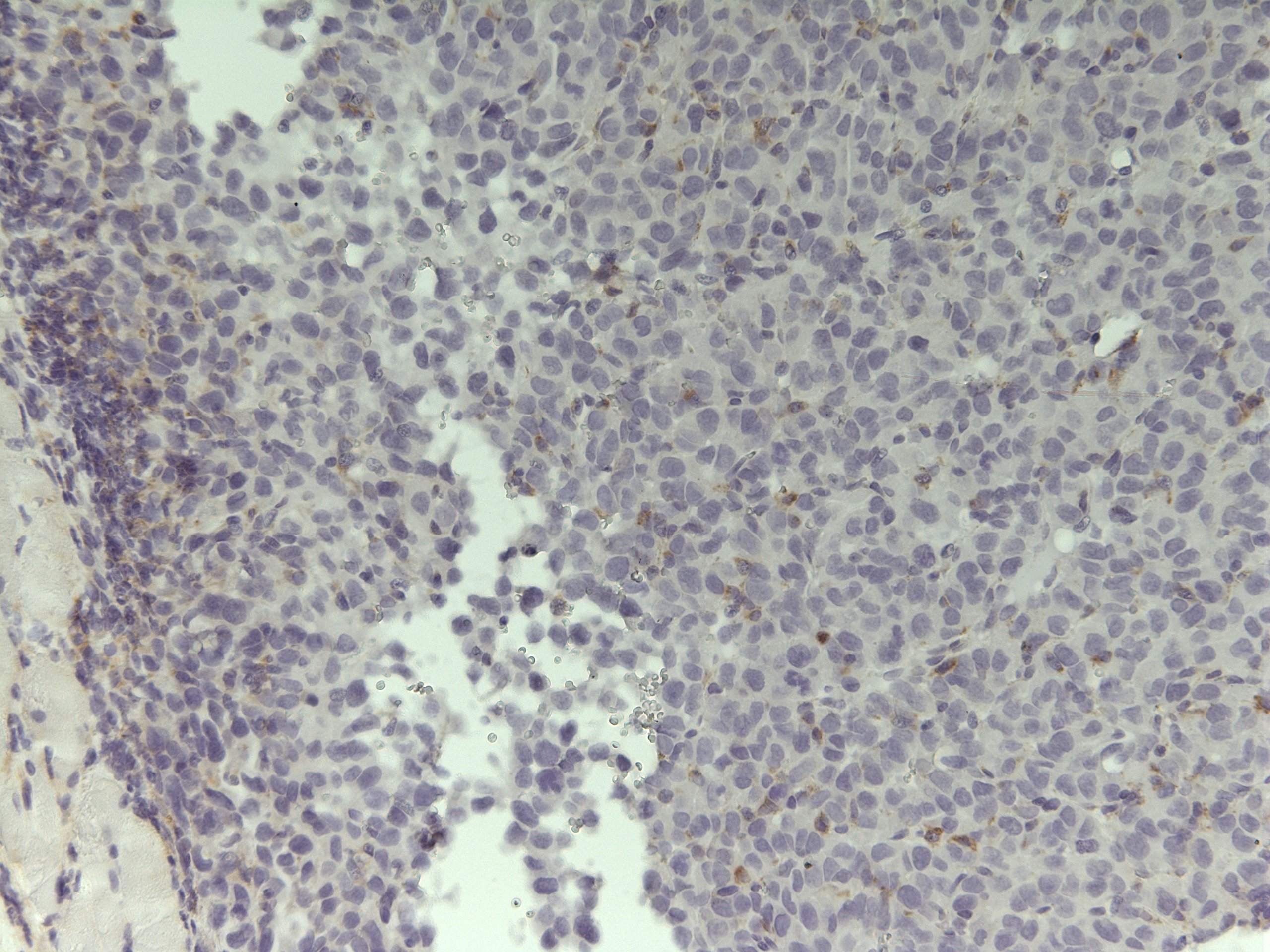

Supplement: Supplementary file 5 — Source data Fig. 3 [file 44321_2025_210_MOESM5_ESM.zip › Figure 3/3D/IHC_HT1080_LAMP-2A-KO_LAMP-2A.jpg]

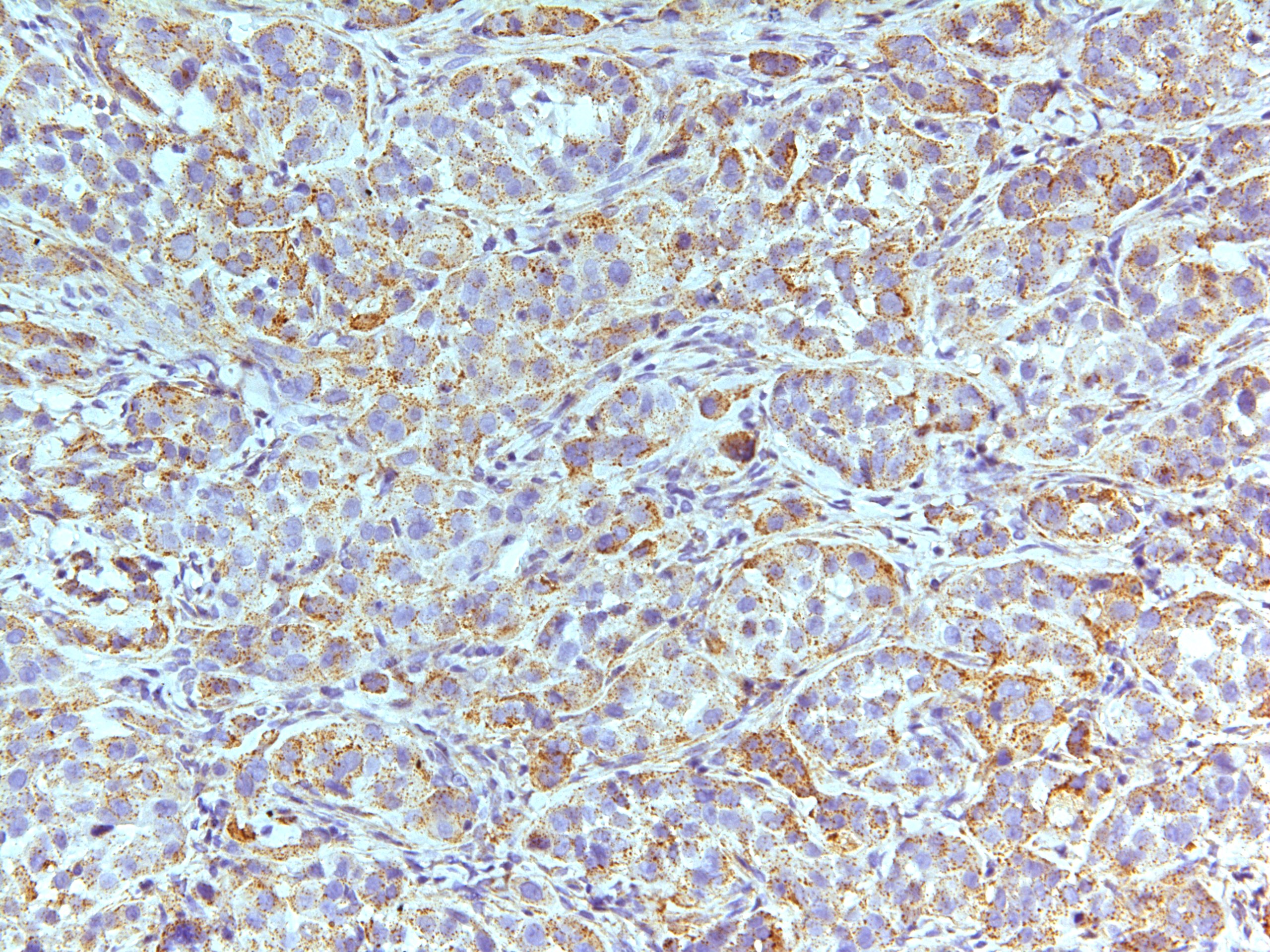

Supplement: Supplementary file 5 — Source data Fig. 3 [file 44321_2025_210_MOESM5_ESM.zip › Figure 3/3D/IHC_A549_WT_LAMP-2A.jpg]

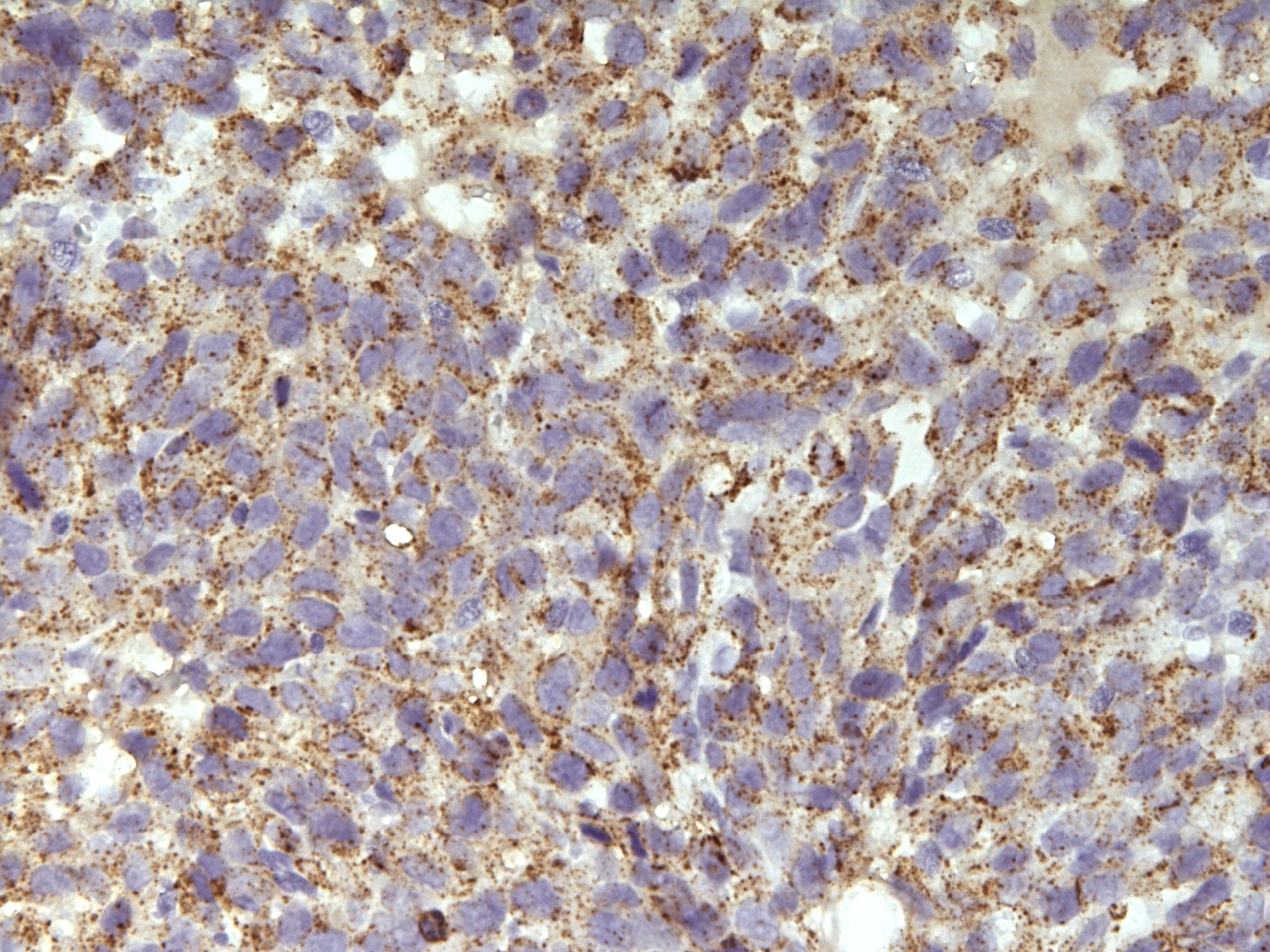

Supplement: Supplementary file 5 — Source data Fig. 3 [file 44321_2025_210_MOESM5_ESM.zip › Figure 3/3D/IHC_HT1080_LAMP-2A-KO_LAMP1.jpg]

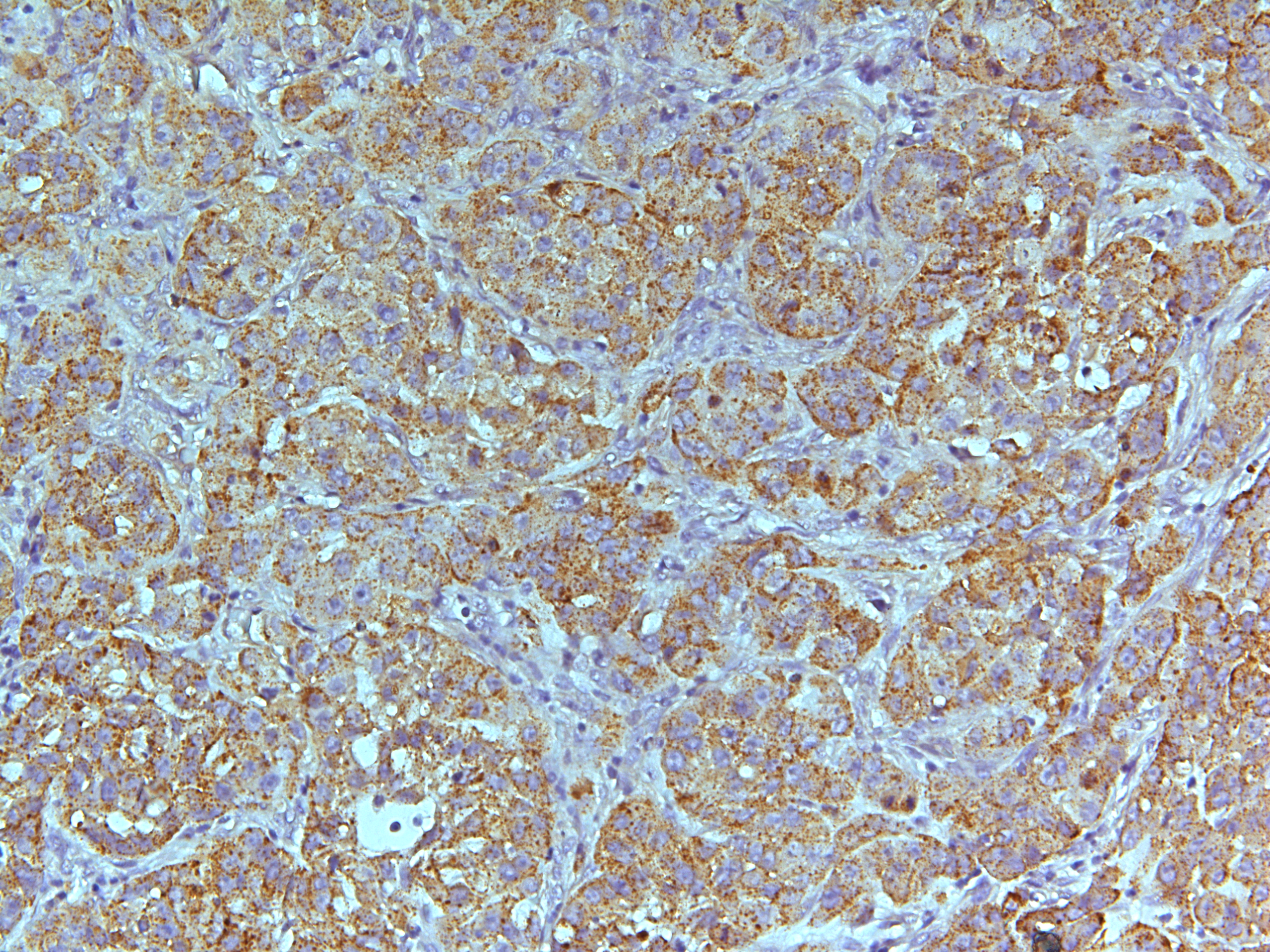

Supplement: Supplementary file 5 — Source data Fig. 3 [file 44321_2025_210_MOESM5_ESM.zip › Figure 3/3D/IHC_A549_LAMP-2A-KO_LAMP1.tif]

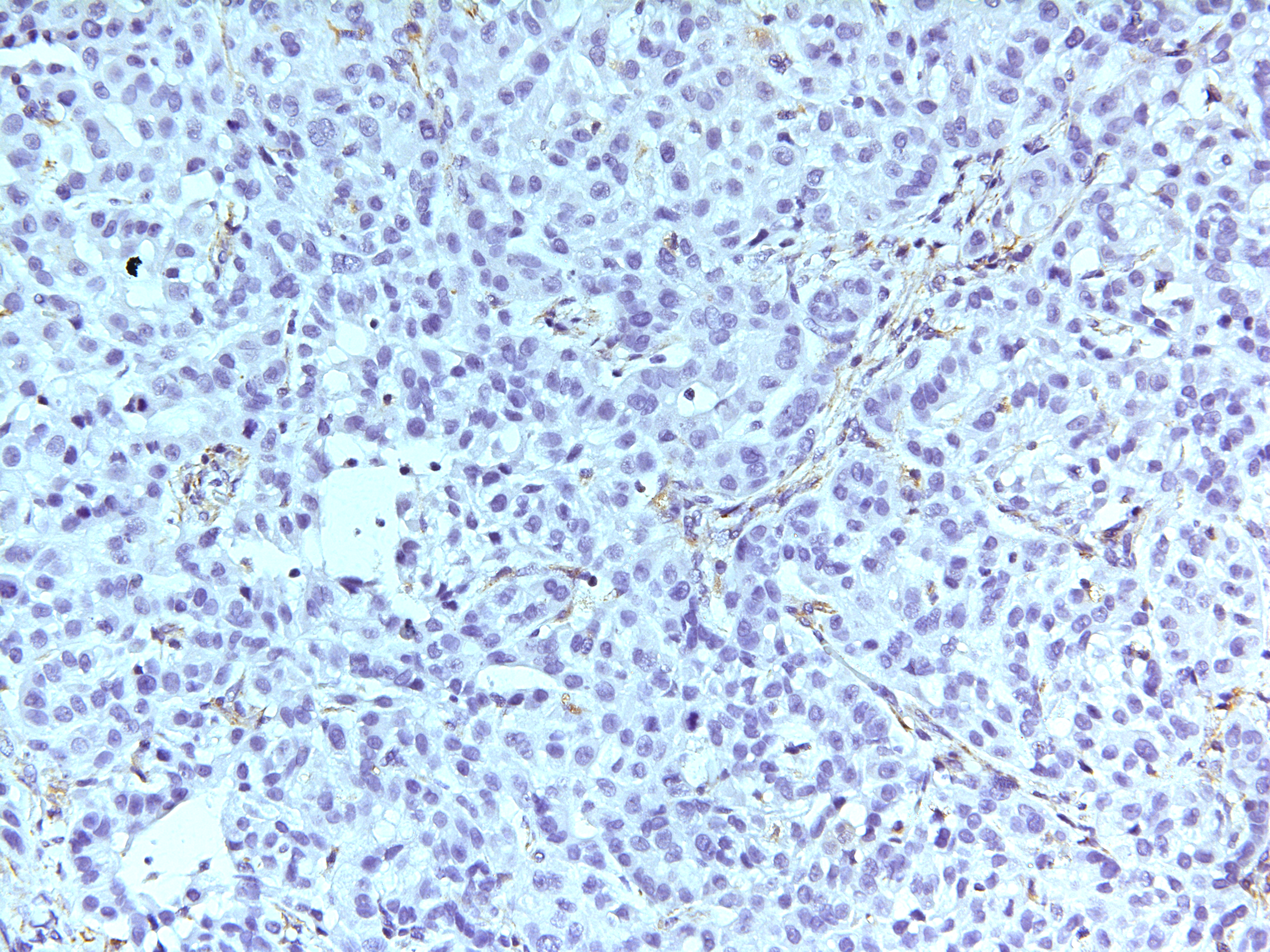

Supplement: Supplementary file 5 — Source data Fig. 3 [file 44321_2025_210_MOESM5_ESM.zip › Figure 3/3D/IHC_A549_LAMP-2A-KO_LAMP-2A.tif]

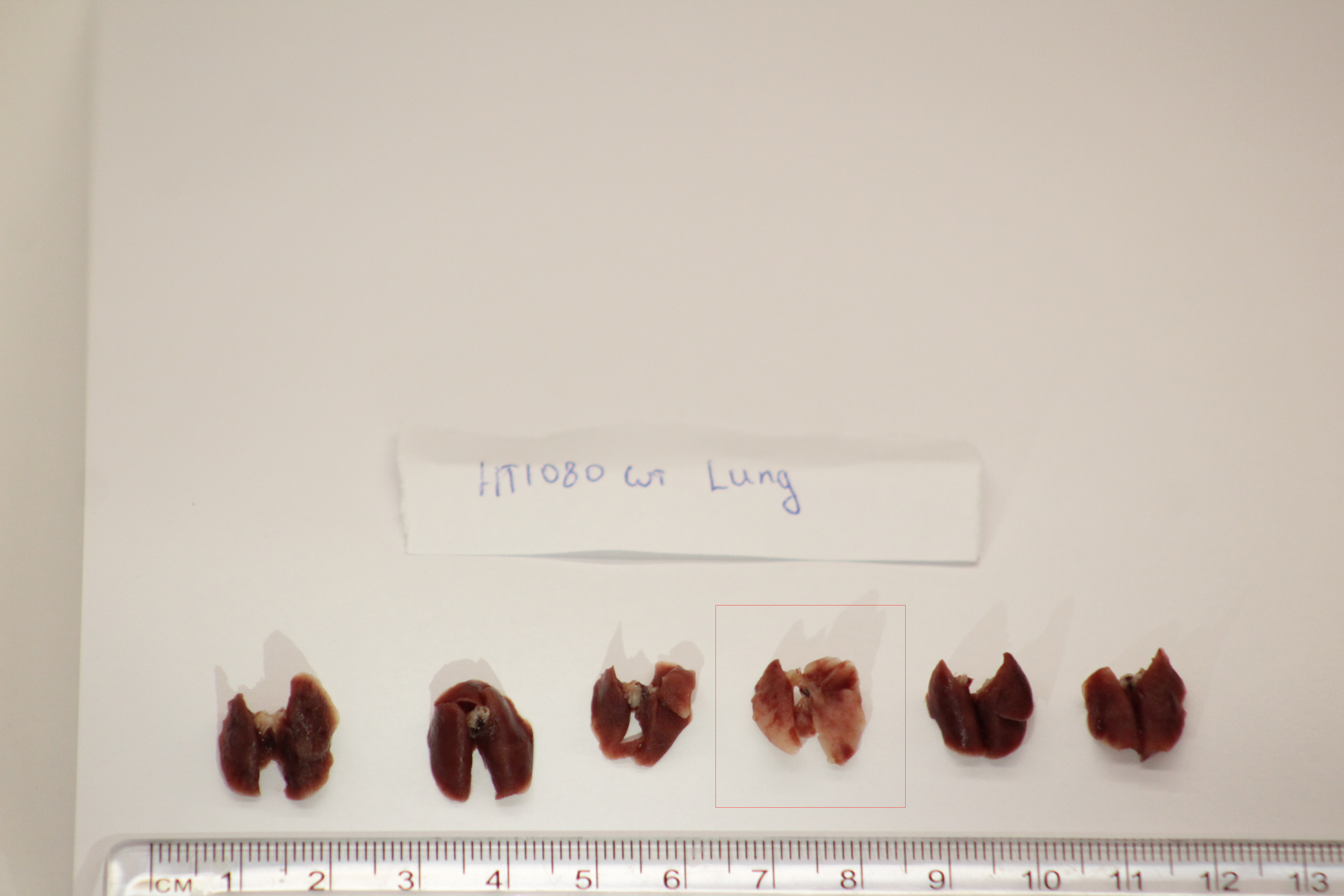

Supplement: Supplementary file 6 — Source data Fig. 4 [file 44321_2025_210_MOESM6_ESM.zip › Figure 4/4D/HT1080 WT - Lungs.tif]

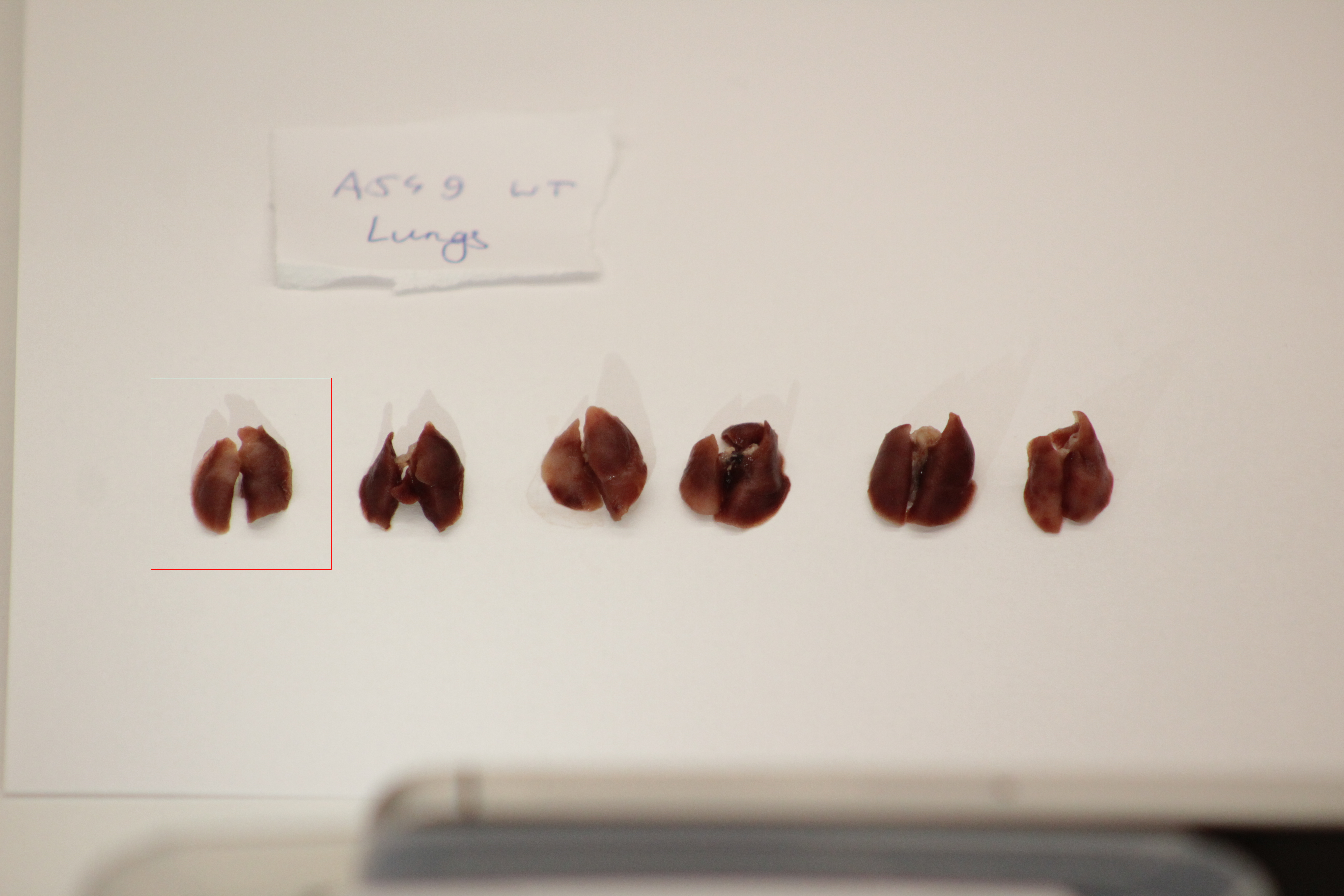

Supplement: Supplementary file 6 — Source data Fig. 4 [file 44321_2025_210_MOESM6_ESM.zip › Figure 4/4D/A549 WT - Lungs.tif]

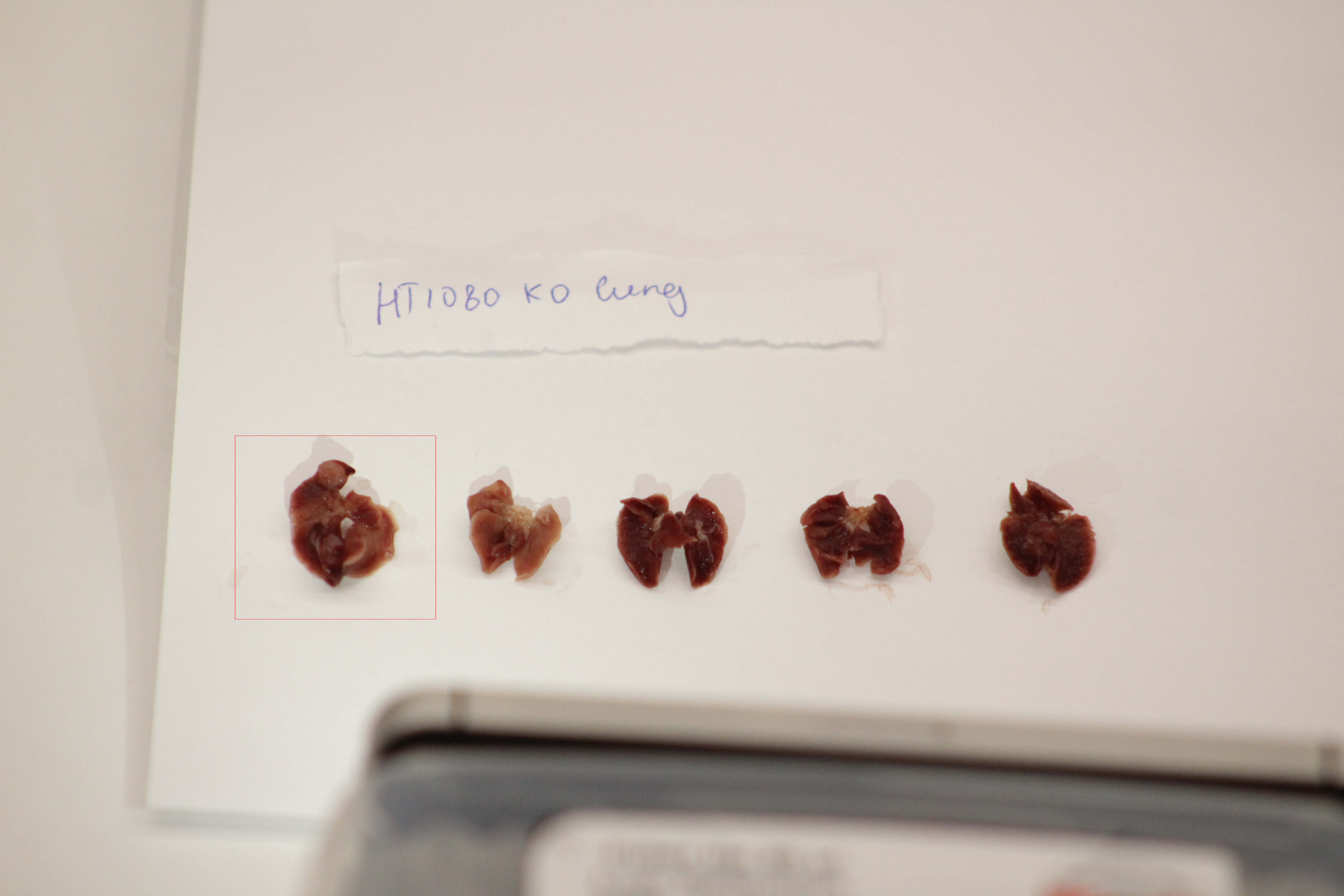

Supplement: Supplementary file 6 — Source data Fig. 4 [file 44321_2025_210_MOESM6_ESM.zip › Figure 4/4D/HT1080 KO - Lungs.tif]

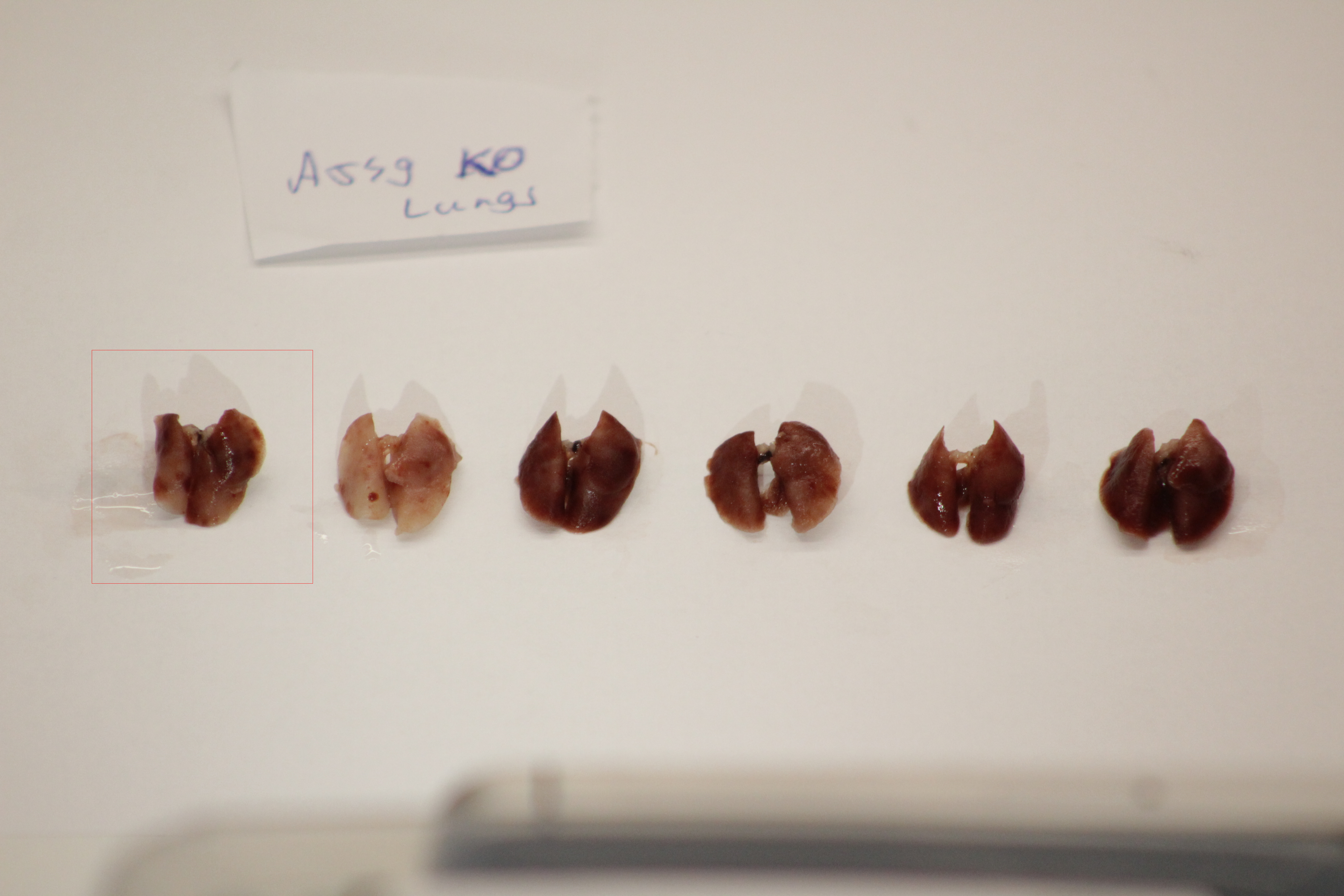

Supplement: Supplementary file 6 — Source data Fig. 4 [file 44321_2025_210_MOESM6_ESM.zip › Figure 4/4D/A549 KO - Lungs.tif]

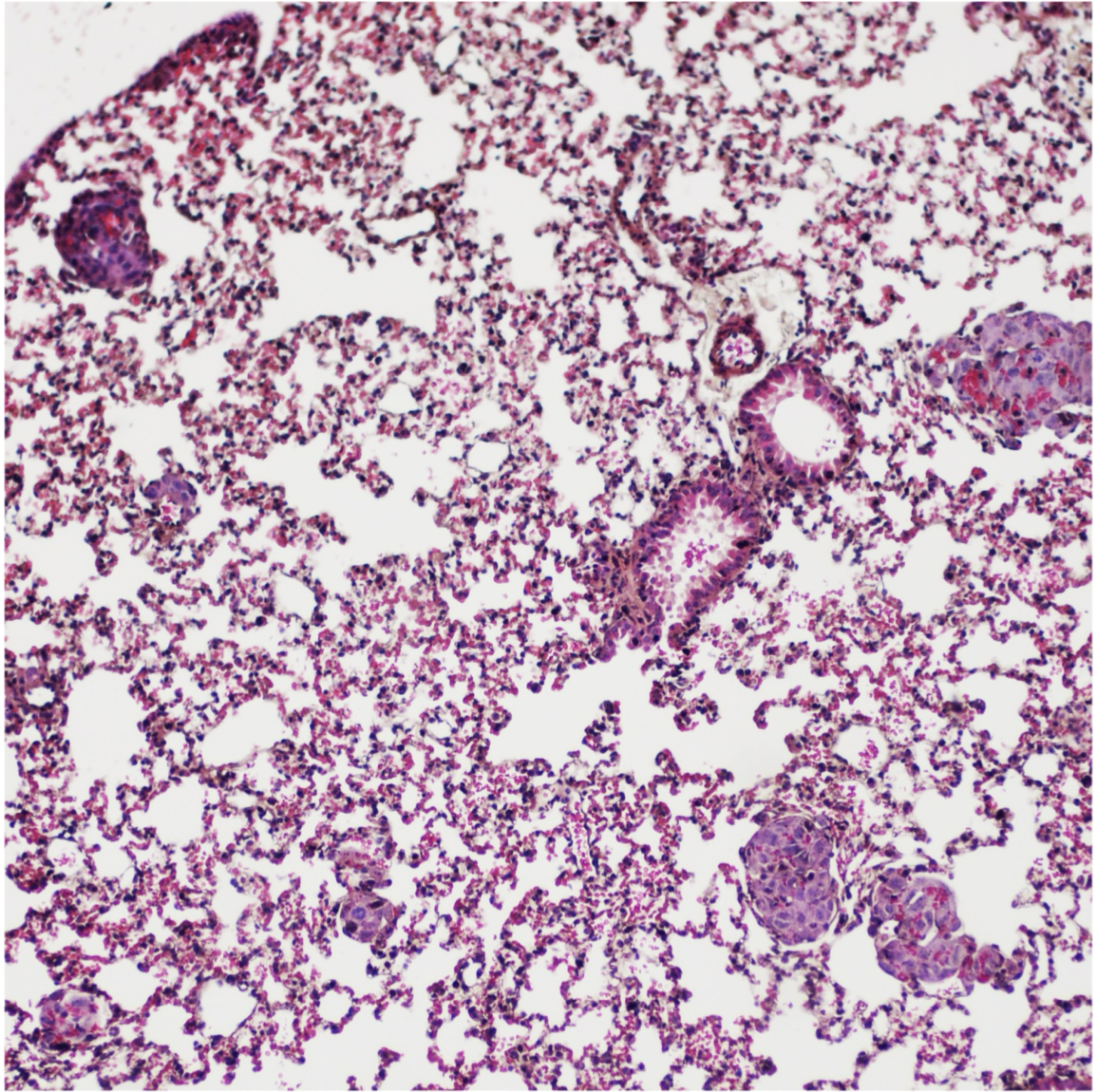

Supplement: Supplementary file 6 — Source data Fig. 4 [file 44321_2025_210_MOESM6_ESM.zip › Figure 4/4F/HT1080 WT - surface metastasis lung.tif]

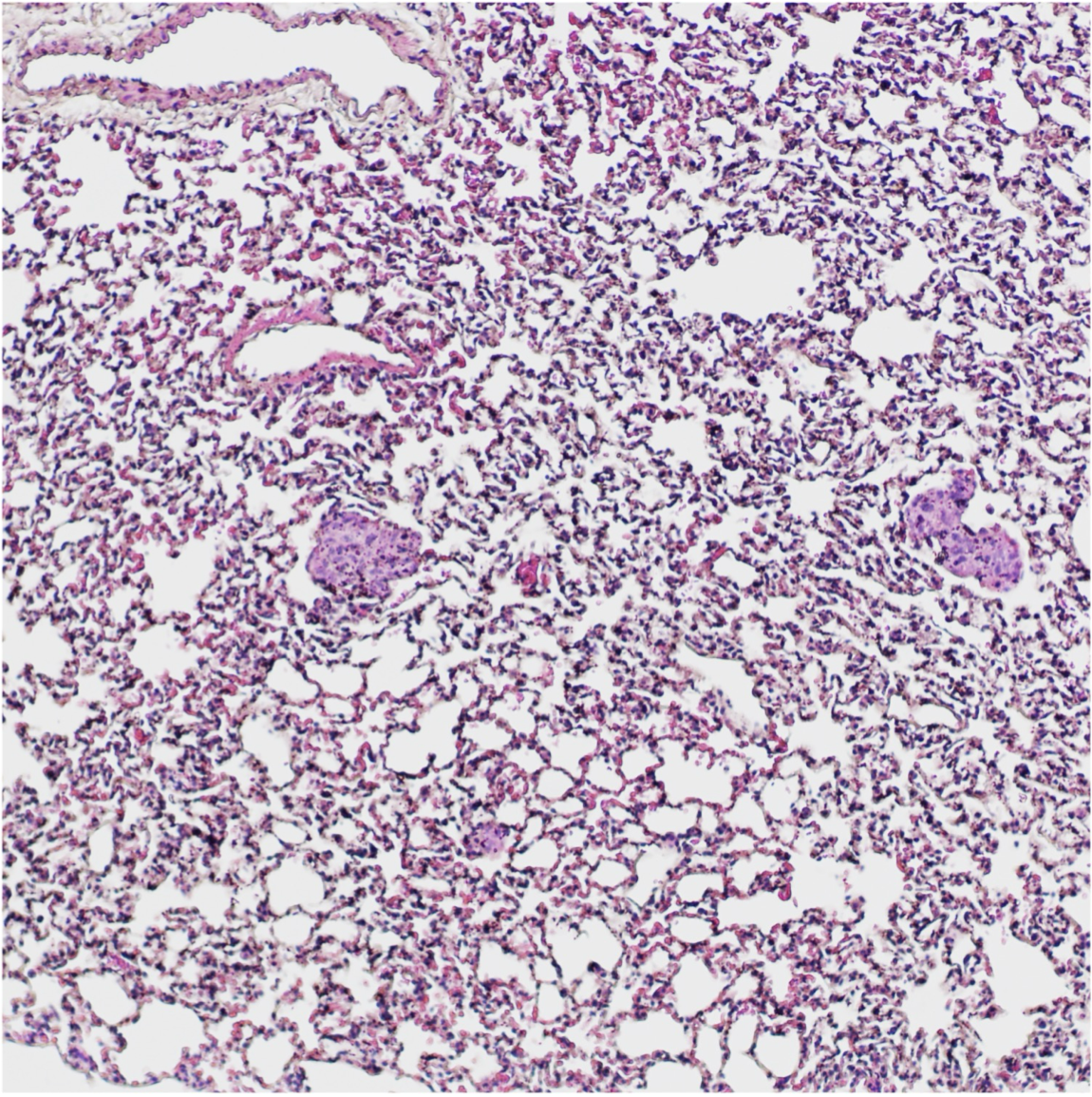

Supplement: Supplementary file 6 — Source data Fig. 4 [file 44321_2025_210_MOESM6_ESM.zip › Figure 4/4F/HT1080 WT - lung.tif]

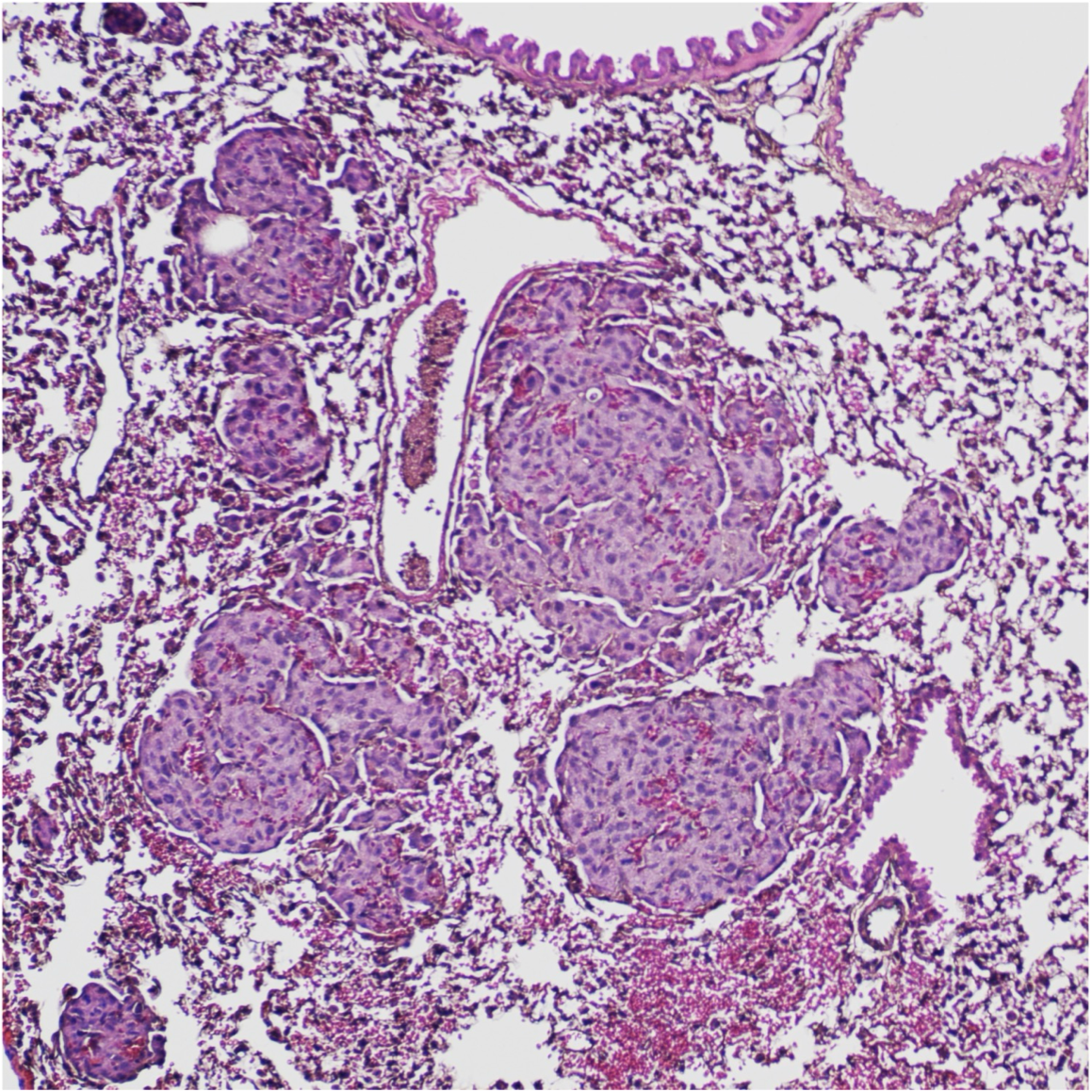

Supplement: Supplementary file 6 — Source data Fig. 4 [file 44321_2025_210_MOESM6_ESM.zip › Figure 4/4F/HT1080 KO - lung .tif]

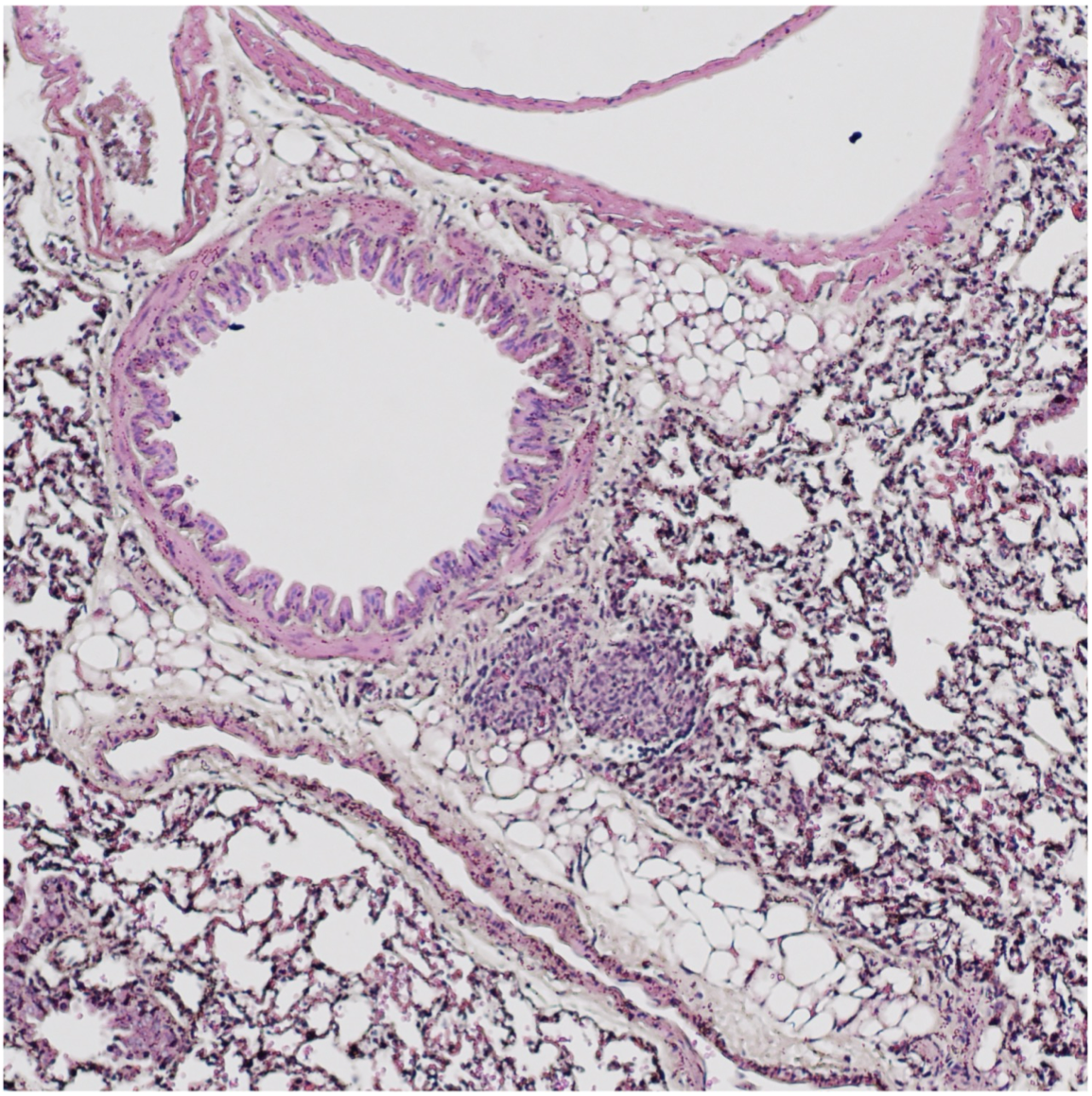

Supplement: Supplementary file 6 — Source data Fig. 4 [file 44321_2025_210_MOESM6_ESM.zip › Figure 4/4F/A549 KO - lung.tif]

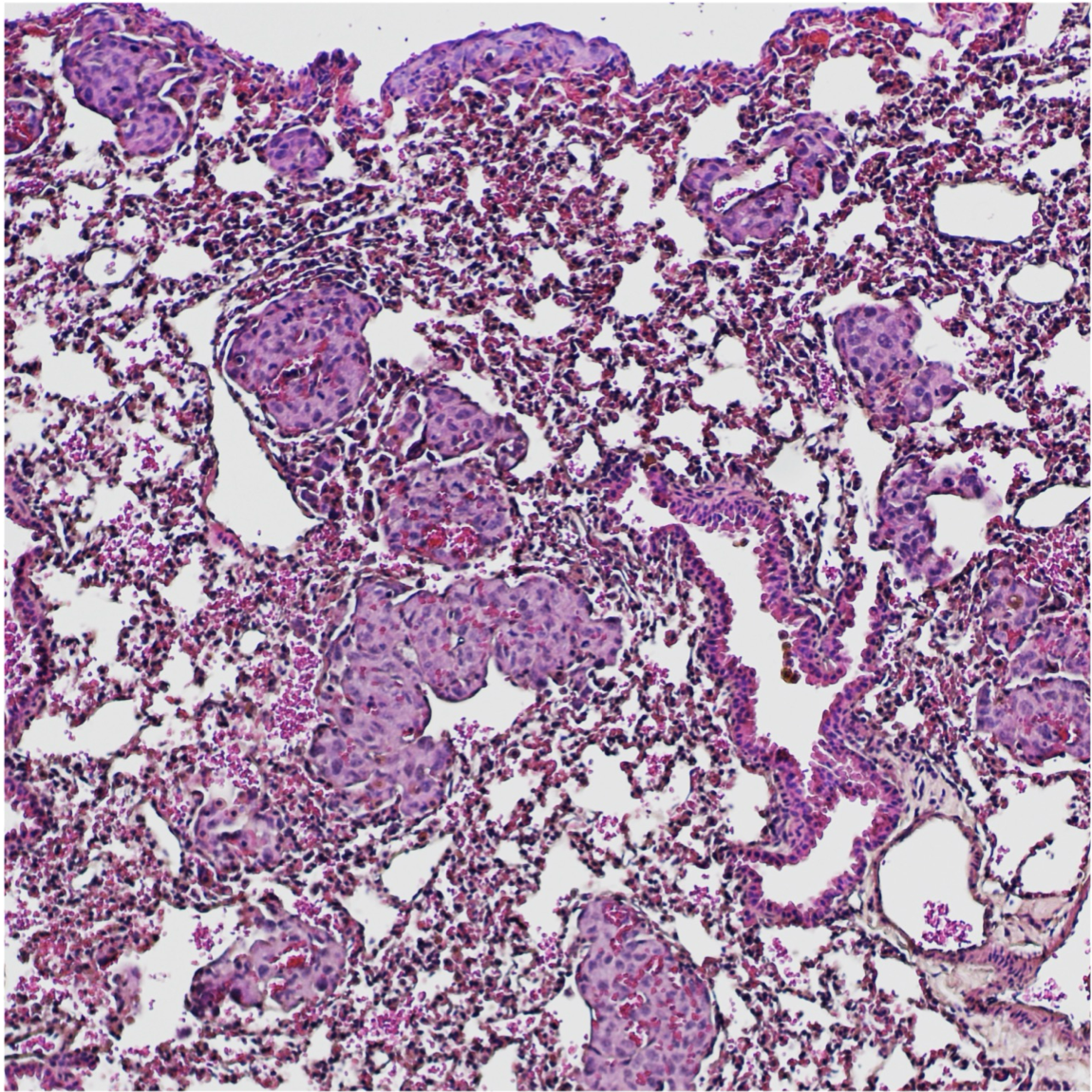

Supplement: Supplementary file 6 — Source data Fig. 4 [file 44321_2025_210_MOESM6_ESM.zip › Figure 4/4F/HT1080 KO - surface metastasis lung .tif]

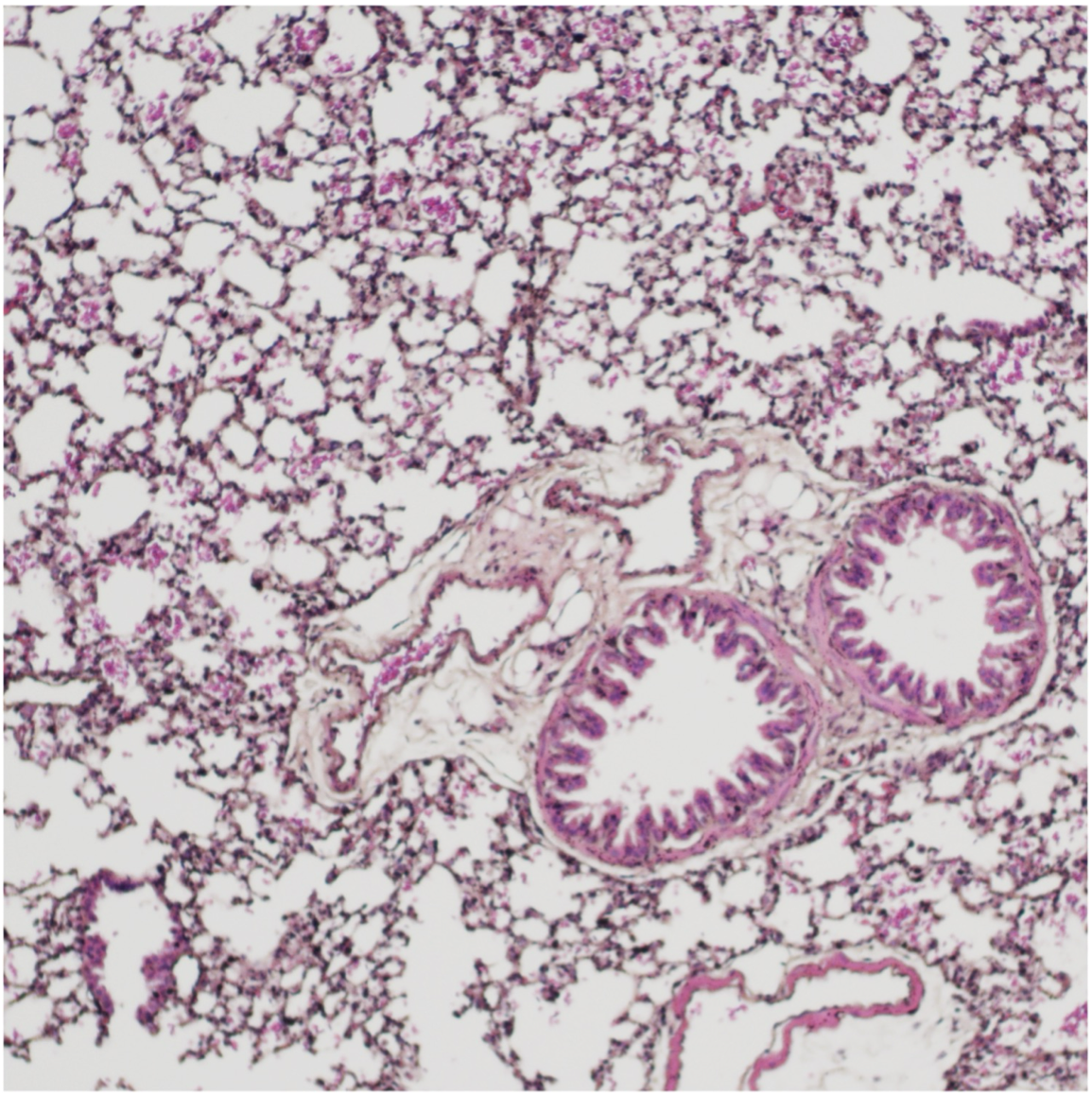

Supplement: Supplementary file 6 — Source data Fig. 4 [file 44321_2025_210_MOESM6_ESM.zip › Figure 4/4F/A549 WT - lung.tif]

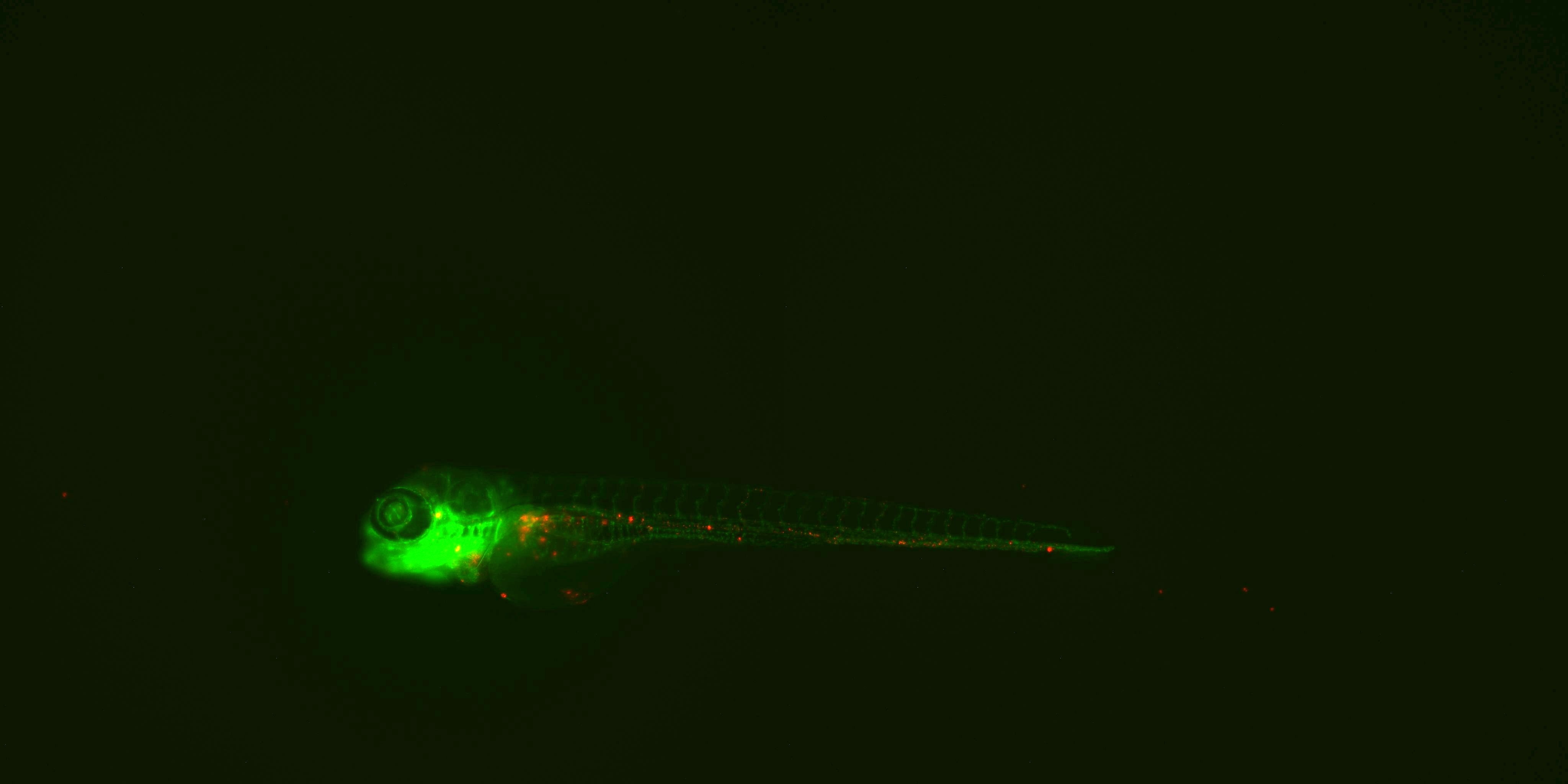

Supplement: Supplementary file 6 — Source data Fig. 4 [file 44321_2025_210_MOESM6_ESM.zip › Figure 4/4H/KO.jpg]

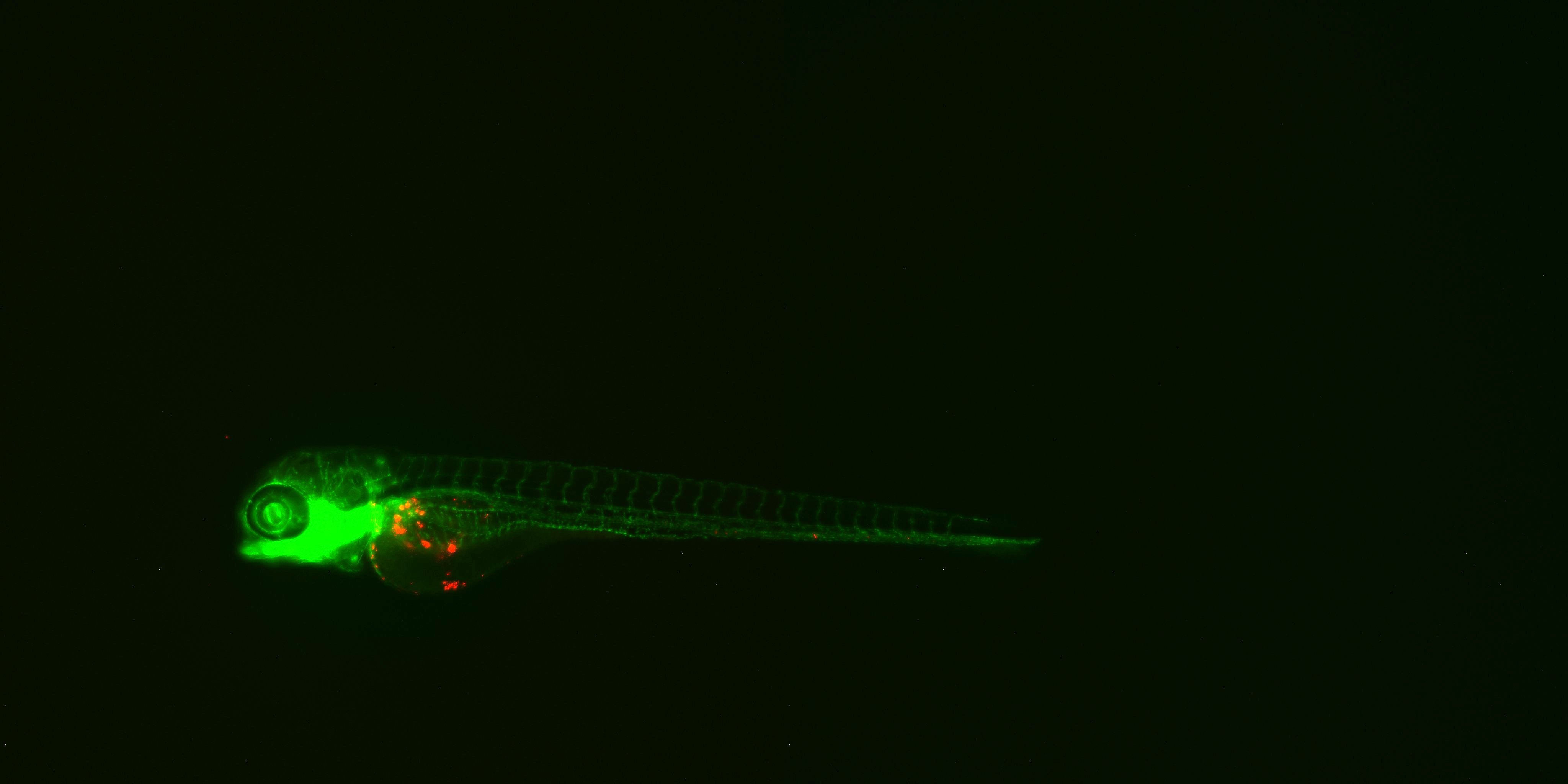

Supplement: Supplementary file 6 — Source data Fig. 4 [file 44321_2025_210_MOESM6_ESM.zip › Figure 4/4H/WT.jpg]

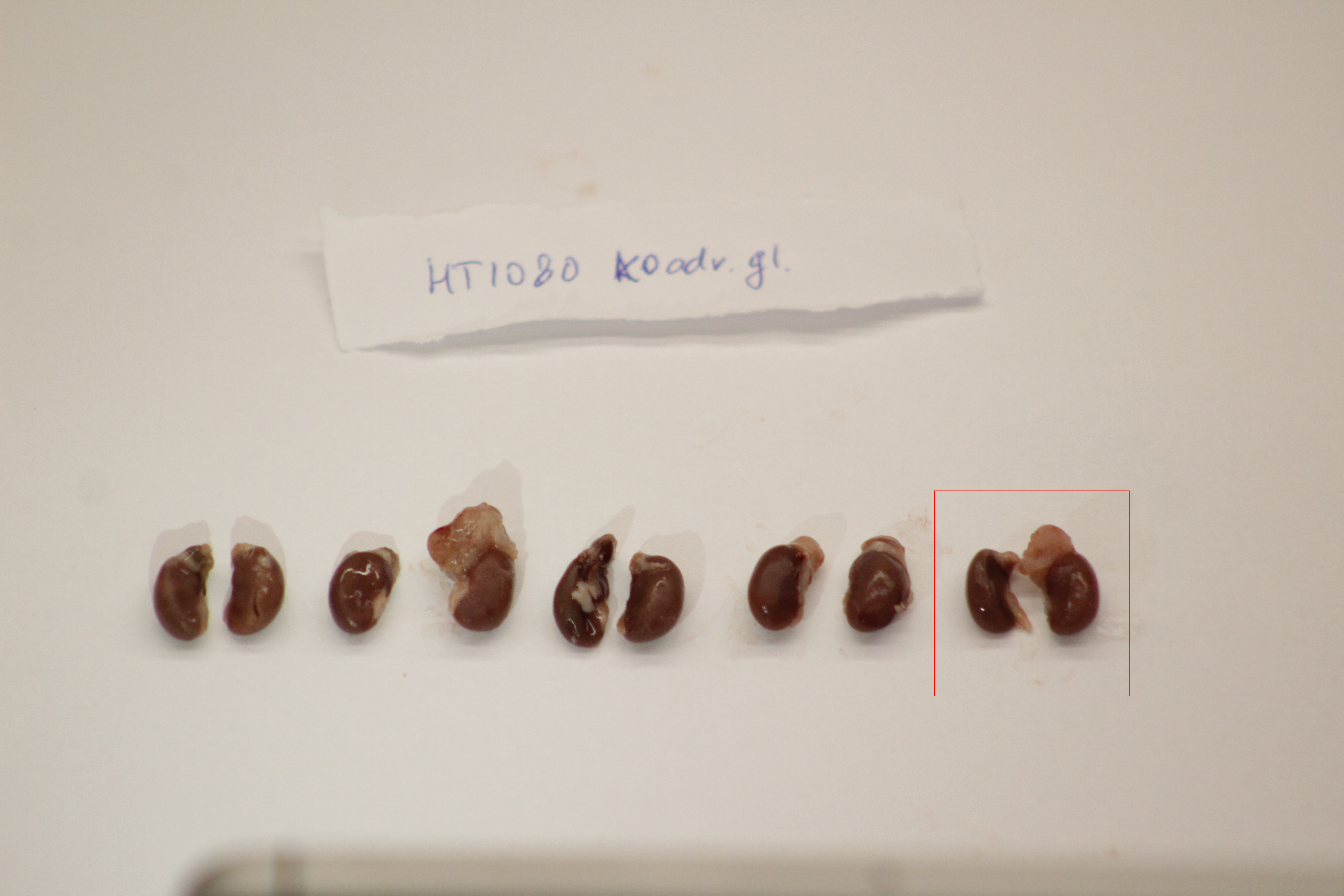

Supplement: Supplementary file 6 — Source data Fig. 4 [file 44321_2025_210_MOESM6_ESM.zip › Figure 4/4G/HT1080 KO - Adrenal glands.tif]

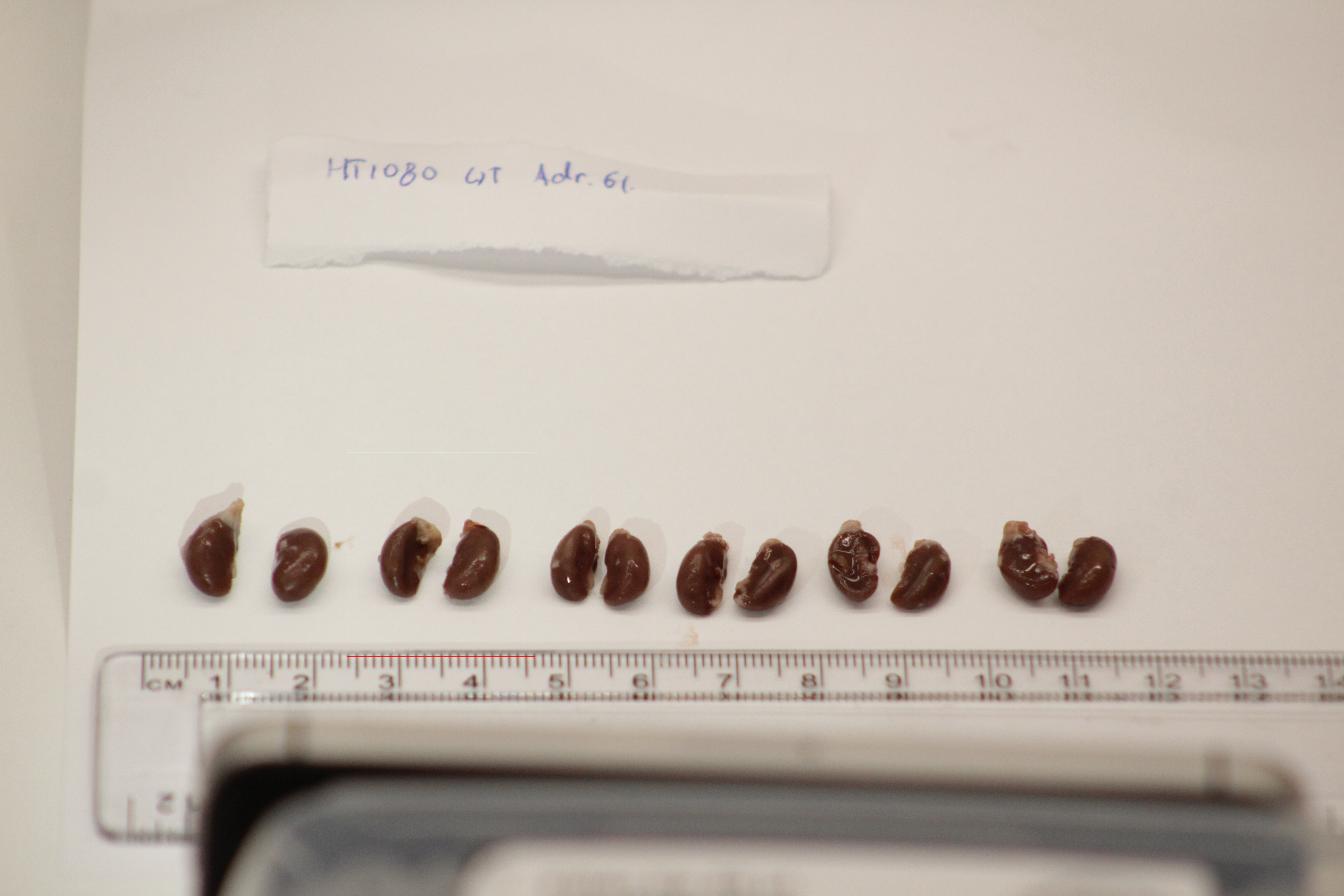

Supplement: Supplementary file 6 — Source data Fig. 4 [file 44321_2025_210_MOESM6_ESM.zip › Figure 4/4G/HT1080 WT - Adrenal glands.tif]

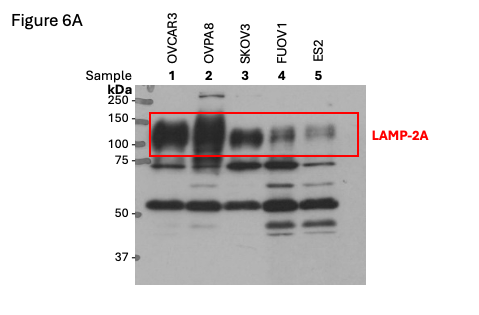

Supplement: Supplementary file 8 — Source data Fig. 6 [file 44321_2025_210_MOESM8_ESM.zip › Figure 6/6A/1. Figure 6A - LAMP-2A.tiff]

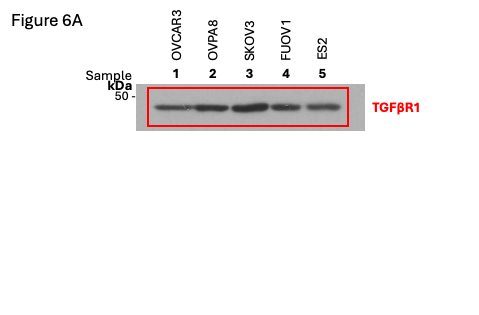

Supplement: Supplementary file 8 — Source data Fig. 6 [file 44321_2025_210_MOESM8_ESM.zip › Figure 6/6A/9. Figure 6A - TGFBR1.tiff]

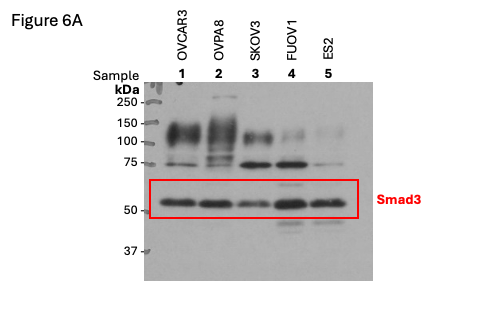

Supplement: Supplementary file 8 — Source data Fig. 6 [file 44321_2025_210_MOESM8_ESM.zip › Figure 6/6A/13. Figure 6A - Smad3.tiff]

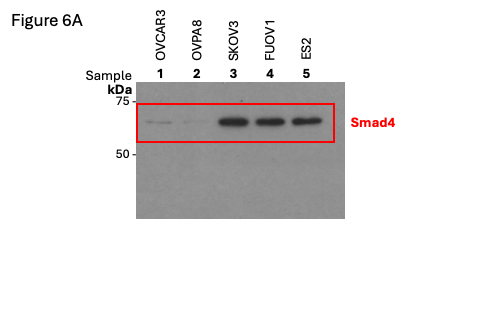

Supplement: Supplementary file 8 — Source data Fig. 6 [file 44321_2025_210_MOESM8_ESM.zip › Figure 6/6A/14. Figure 6A - Smad4.tiff]

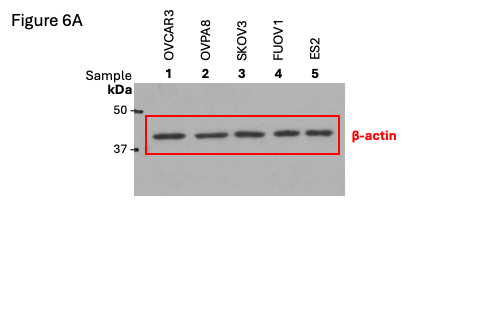

Supplement: Supplementary file 8 — Source data Fig. 6 [file 44321_2025_210_MOESM8_ESM.zip › Figure 6/6A/7. Figure 6A - b-actin (2).tiff]

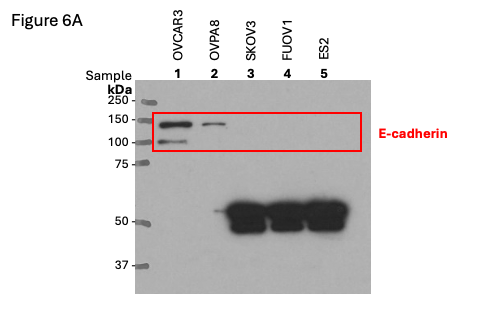

Supplement: Supplementary file 8 — Source data Fig. 6 [file 44321_2025_210_MOESM8_ESM.zip › Figure 6/6A/4. Figure 6A - E-cadherin.tiff]

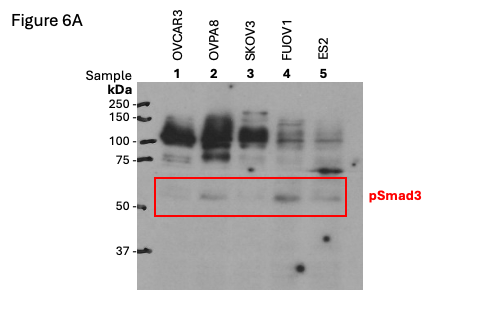

Supplement: Supplementary file 8 — Source data Fig. 6 [file 44321_2025_210_MOESM8_ESM.zip › Figure 6/6A/12. Figure 6A - pSmad3.tiff]

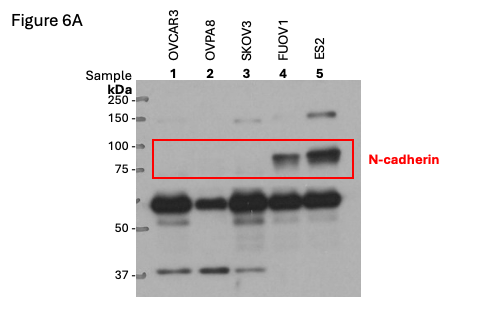

Supplement: Supplementary file 8 — Source data Fig. 6 [file 44321_2025_210_MOESM8_ESM.zip › Figure 6/6A/5. Figure 6A - N-cadherin.tiff]

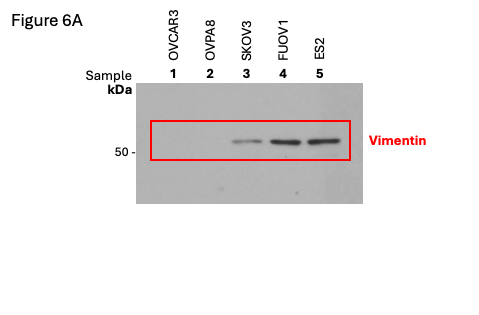

Supplement: Supplementary file 8 — Source data Fig. 6 [file 44321_2025_210_MOESM8_ESM.zip › Figure 6/6A/6. Figure 6A - Vimentin.tiff]

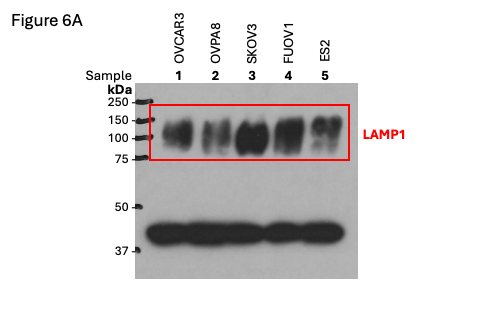

Supplement: Supplementary file 8 — Source data Fig. 6 [file 44321_2025_210_MOESM8_ESM.zip › Figure 6/6A/2. Figure 6A - LAMP1.tiff]

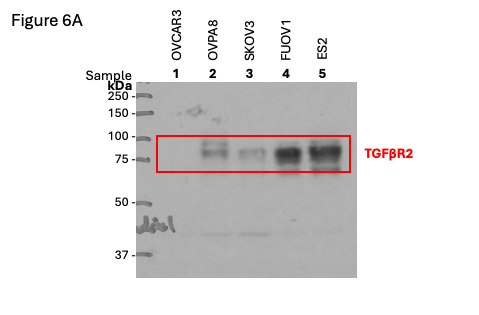

Supplement: Supplementary file 8 — Source data Fig. 6 [file 44321_2025_210_MOESM8_ESM.zip › Figure 6/6A/8. Figure 6A - TGFBR2.tiff]

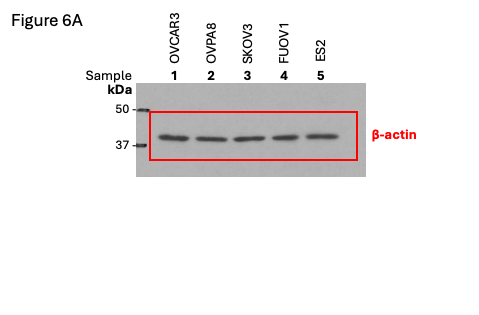

Supplement: Supplementary file 8 — Source data Fig. 6 [file 44321_2025_210_MOESM8_ESM.zip › Figure 6/6A/3. Figure 6A - b-actin (1).tiff]

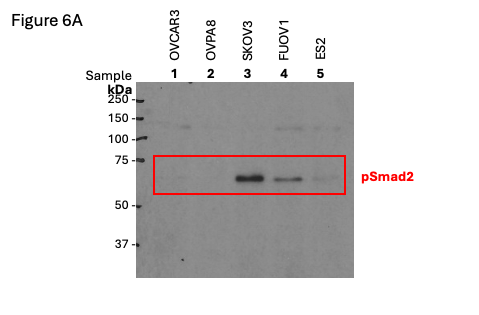

Supplement: Supplementary file 8 — Source data Fig. 6 [file 44321_2025_210_MOESM8_ESM.zip › Figure 6/6A/10. Figure 6A - pSmad2.tiff]

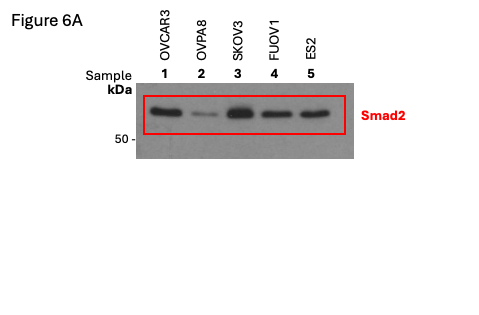

Supplement: Supplementary file 8 — Source data Fig. 6 [file 44321_2025_210_MOESM8_ESM.zip › Figure 6/6A/11. Figure 6A - Smad2.tiff]

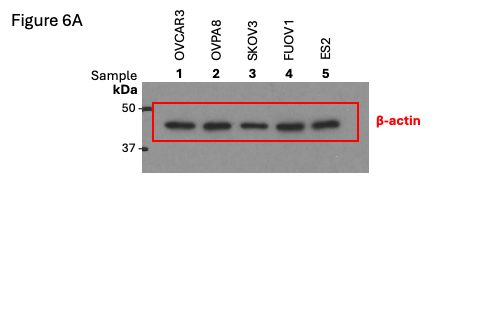

Supplement: Supplementary file 8 — Source data Fig. 6 [file 44321_2025_210_MOESM8_ESM.zip › Figure 6/6A/15. Figure 6A - b-actin (3).tiff]

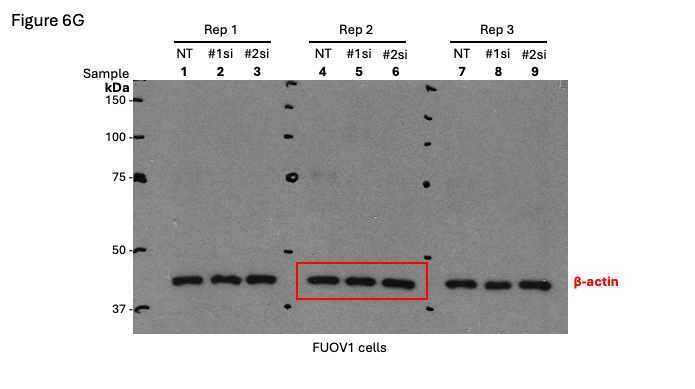

Supplement: Supplementary file 8 — Source data Fig. 6 [file 44321_2025_210_MOESM8_ESM.zip › Figure 6/6G/Figure 6G - b-actin.tiff]

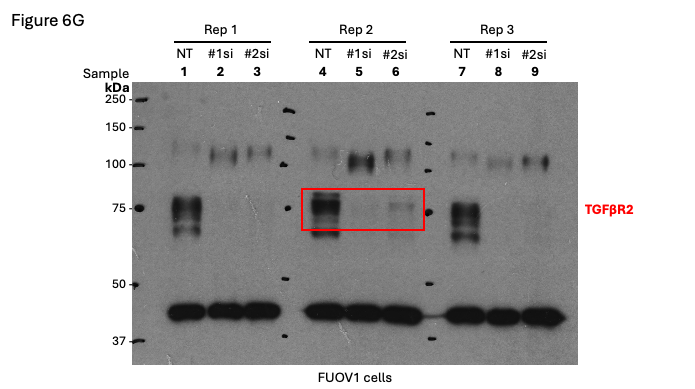

Supplement: Supplementary file 8 — Source data Fig. 6 [file 44321_2025_210_MOESM8_ESM.zip › Figure 6/6G/Figure 6G - TGFBR2.tiff]

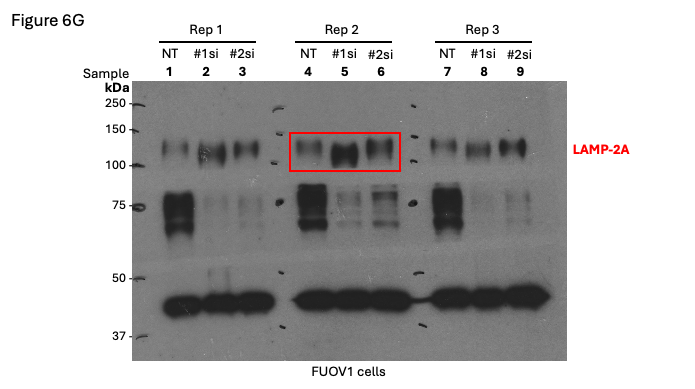

Supplement: Supplementary file 8 — Source data Fig. 6 [file 44321_2025_210_MOESM8_ESM.zip › Figure 6/6G/Figure 6G - LAMP-2A.tiff]

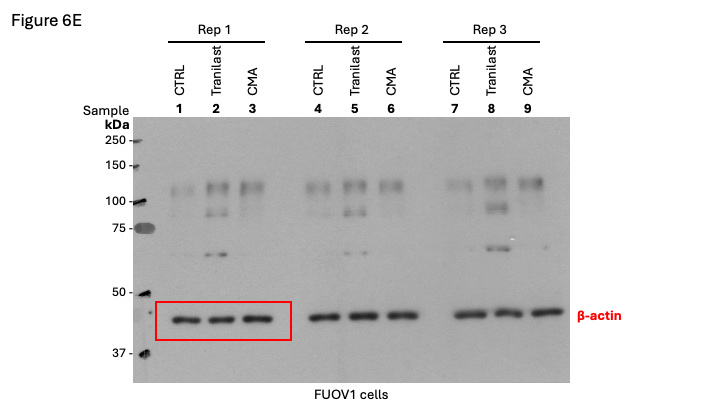

Supplement: Supplementary file 8 — Source data Fig. 6 [file 44321_2025_210_MOESM8_ESM.zip › Figure 6/6E/Figure 6E - b-actin.tiff]

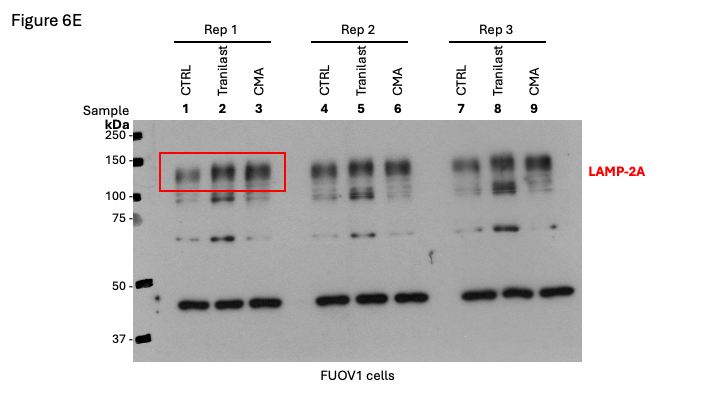

Supplement: Supplementary file 8 — Source data Fig. 6 [file 44321_2025_210_MOESM8_ESM.zip › Figure 6/6E/Figure 6E- LAMP-2A.tiff]

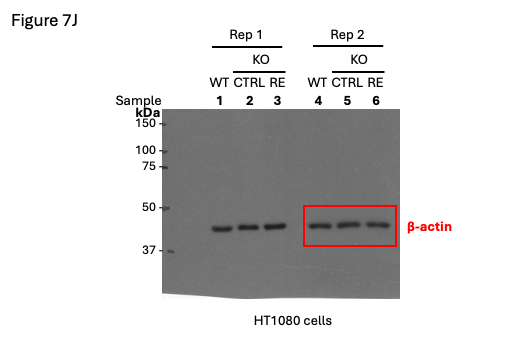

Supplement: Supplementary file 9 — Source data Fig. 7 [file 44321_2025_210_MOESM9_ESM.zip › Figure 7/7J/b-actin.tiff]

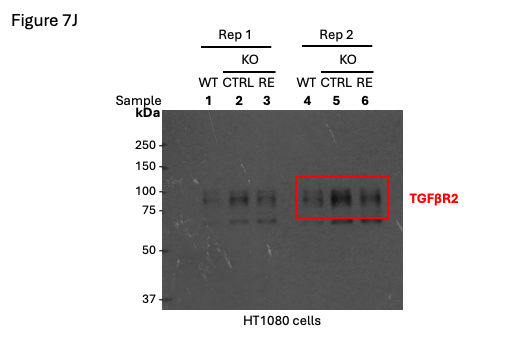

Supplement: Supplementary file 9 — Source data Fig. 7 [file 44321_2025_210_MOESM9_ESM.zip › Figure 7/7J/TGFBR2.tiff]

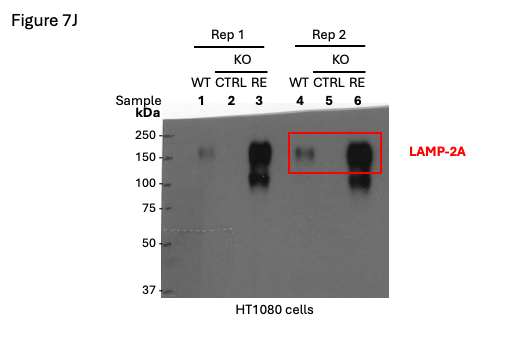

Supplement: Supplementary file 9 — Source data Fig. 7 [file 44321_2025_210_MOESM9_ESM.zip › Figure 7/7J/LAMP-2A.tiff]

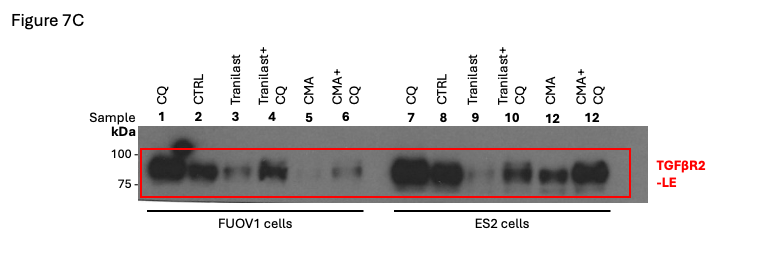

Supplement: Supplementary file 9 — Source data Fig. 7 [file 44321_2025_210_MOESM9_ESM.zip › Figure 7/7C/1. Figure 7C - TGFBR2-LE.tiff]

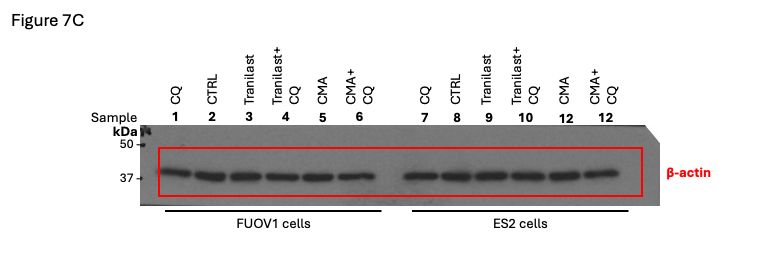

Supplement: Supplementary file 9 — Source data Fig. 7 [file 44321_2025_210_MOESM9_ESM.zip › Figure 7/7C/3. Figure 7C - b-actin.tiff]

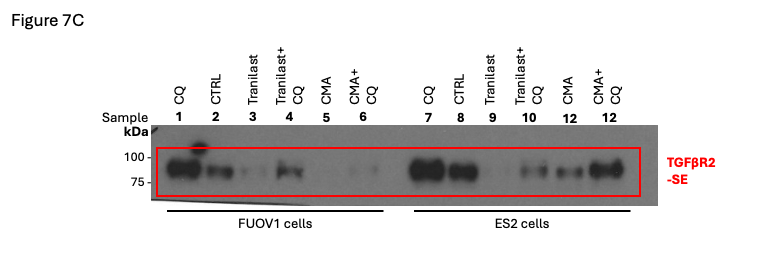

Supplement: Supplementary file 9 — Source data Fig. 7 [file 44321_2025_210_MOESM9_ESM.zip › Figure 7/7C/2. Figure 7C - TGFBR2-SE.tiff]

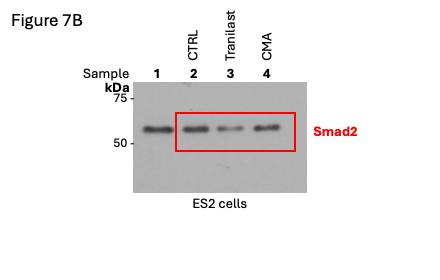

Supplement: Supplementary file 9 — Source data Fig. 7 [file 44321_2025_210_MOESM9_ESM.zip › Figure 7/7B/2. Figure 7B - Smad2.tiff]

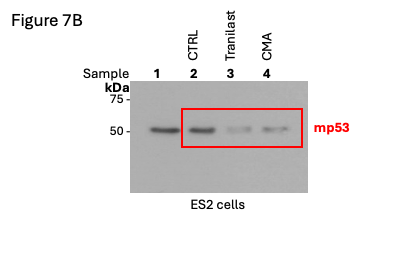

Supplement: Supplementary file 9 — Source data Fig. 7 [file 44321_2025_210_MOESM9_ESM.zip › Figure 7/7B/Figure 7B - mp53.tif]

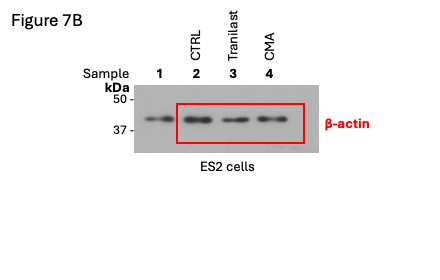

Supplement: Supplementary file 9 — Source data Fig. 7 [file 44321_2025_210_MOESM9_ESM.zip › Figure 7/7B/Figure 7B - b-actin (2).tiff]

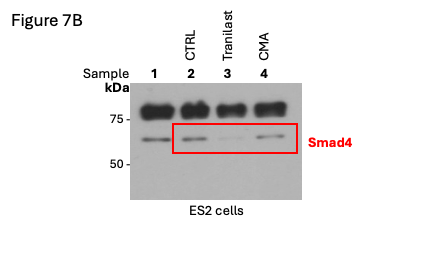

Supplement: Supplementary file 9 — Source data Fig. 7 [file 44321_2025_210_MOESM9_ESM.zip › Figure 7/7B/4. Figure 7B - Smad4.tiff]

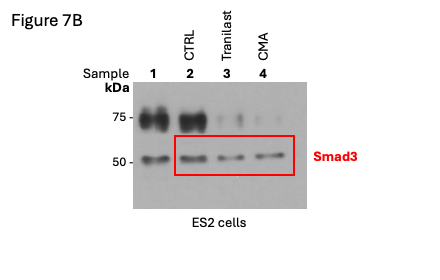

Supplement: Supplementary file 9 — Source data Fig. 7 [file 44321_2025_210_MOESM9_ESM.zip › Figure 7/7B/3. Figure 7B - Smad3.tiff]

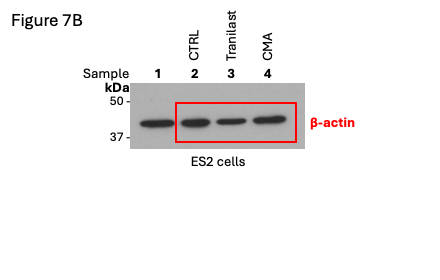

Supplement: Supplementary file 9 — Source data Fig. 7 [file 44321_2025_210_MOESM9_ESM.zip › Figure 7/7B/4. Figure 7B - b-actin (1).tiff]

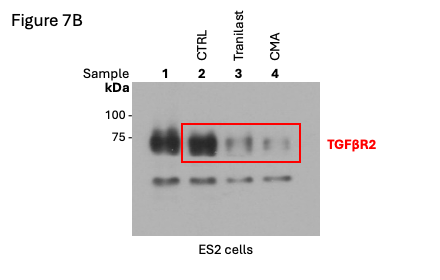

Supplement: Supplementary file 9 — Source data Fig. 7 [file 44321_2025_210_MOESM9_ESM.zip › Figure 7/7B/1. Figure 7B - TGFBR2.tiff]

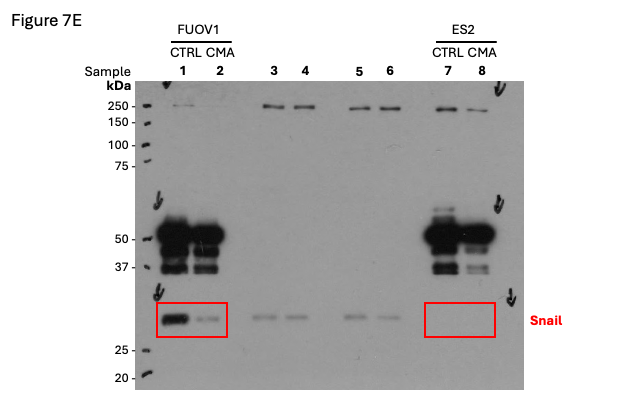

Supplement: Supplementary file 9 — Source data Fig. 7 [file 44321_2025_210_MOESM9_ESM.zip › Figure 7/7E/1. Figure 7E - Snail.tiff]

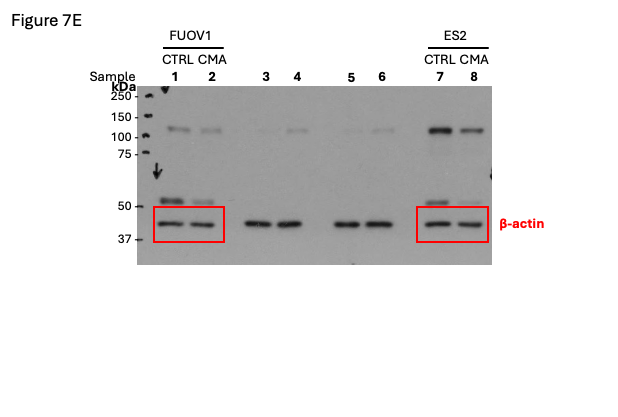

Supplement: Supplementary file 9 — Source data Fig. 7 [file 44321_2025_210_MOESM9_ESM.zip › Figure 7/7E/5. Figure 7E - b-actin.tiff]

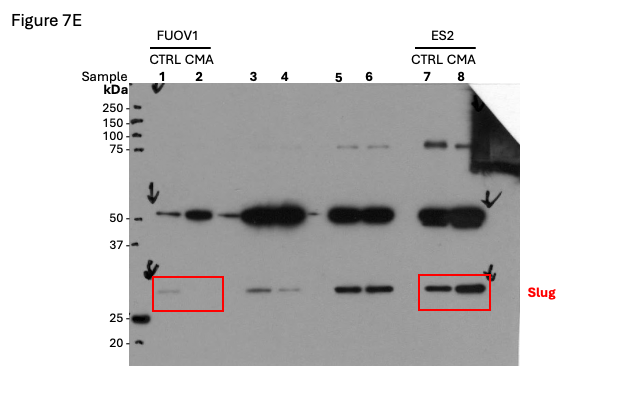

Supplement: Supplementary file 9 — Source data Fig. 7 [file 44321_2025_210_MOESM9_ESM.zip › Figure 7/7E/4. Figure 7E - Slug.tiff]

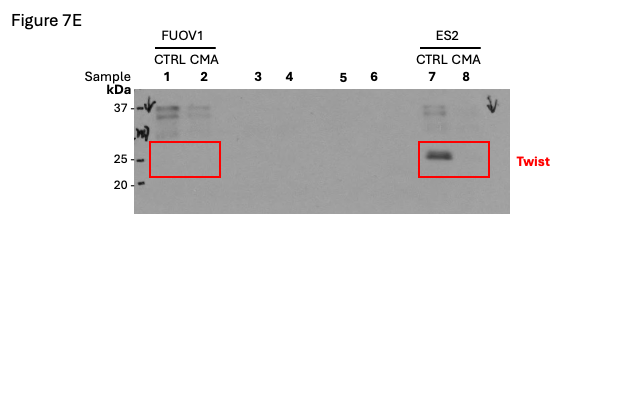

Supplement: Supplementary file 9 — Source data Fig. 7 [file 44321_2025_210_MOESM9_ESM.zip › Figure 7/7E/2. Figure 7E - Twist.tiff]

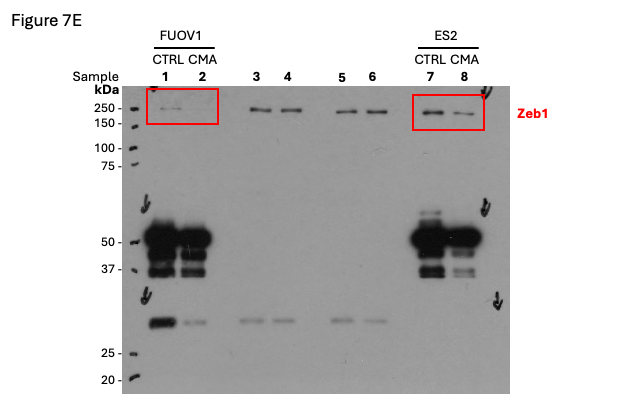

Supplement: Supplementary file 9 — Source data Fig. 7 [file 44321_2025_210_MOESM9_ESM.zip › Figure 7/7E/3. Figure 7E - Zeb1.tiff]

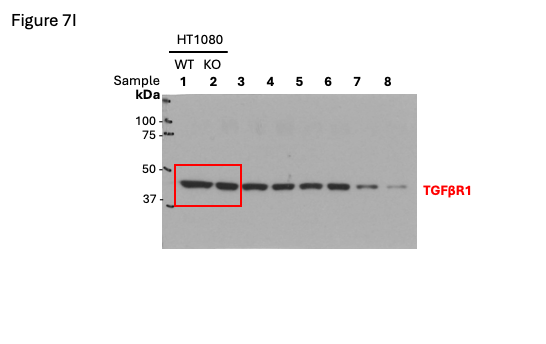

Supplement: Supplementary file 9 — Source data Fig. 7 [file 44321_2025_210_MOESM9_ESM.zip › Figure 7/7I/2. TGFBR1.tiff]

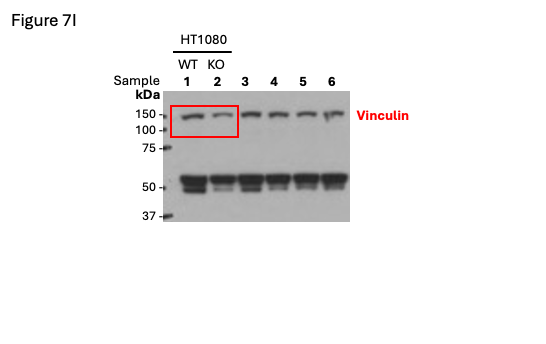

Supplement: Supplementary file 9 — Source data Fig. 7 [file 44321_2025_210_MOESM9_ESM.zip › Figure 7/7I/9. Vinculin (2).tiff]

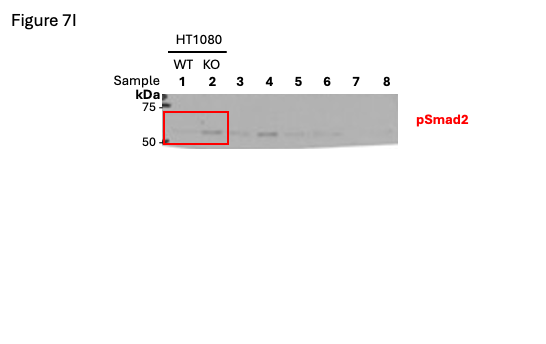

Supplement: Supplementary file 9 — Source data Fig. 7 [file 44321_2025_210_MOESM9_ESM.zip › Figure 7/7I/4. pSmad2.tiff]

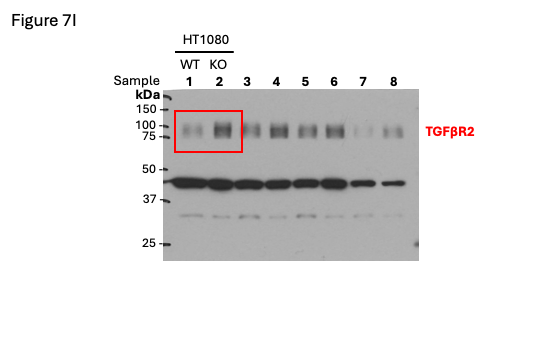

Supplement: Supplementary file 9 — Source data Fig. 7 [file 44321_2025_210_MOESM9_ESM.zip › Figure 7/7I/1. TGFBR2.tiff]

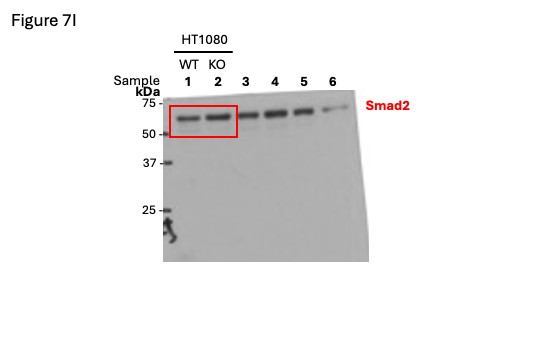

Supplement: Supplementary file 9 — Source data Fig. 7 [file 44321_2025_210_MOESM9_ESM.zip › Figure 7/7I/5. Smad2.tiff]

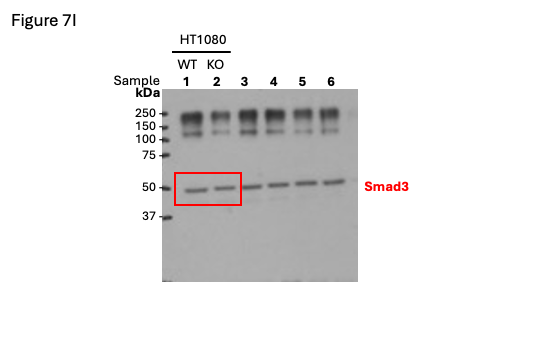

Supplement: Supplementary file 9 — Source data Fig. 7 [file 44321_2025_210_MOESM9_ESM.zip › Figure 7/7I/7. Smad3.tiff]

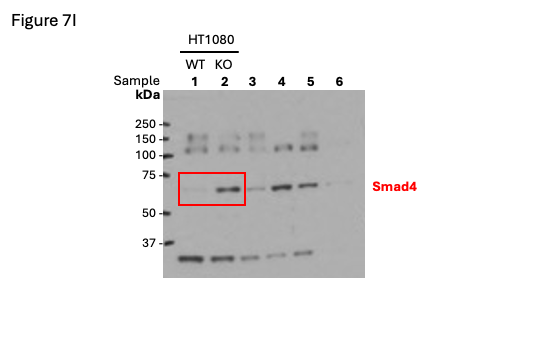

Supplement: Supplementary file 9 — Source data Fig. 7 [file 44321_2025_210_MOESM9_ESM.zip › Figure 7/7I/8. Smad4.tiff]

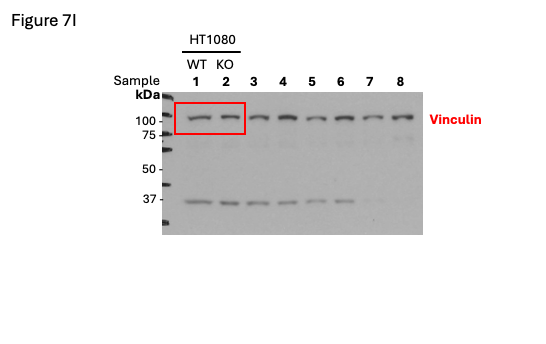

Supplement: Supplementary file 9 — Source data Fig. 7 [file 44321_2025_210_MOESM9_ESM.zip › Figure 7/7I/3. Vinculin (1).tiff]

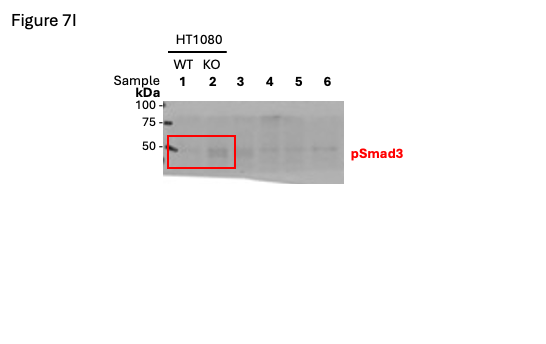

Supplement: Supplementary file 9 — Source data Fig. 7 [file 44321_2025_210_MOESM9_ESM.zip › Figure 7/7I/6. pSmad3.tiff]

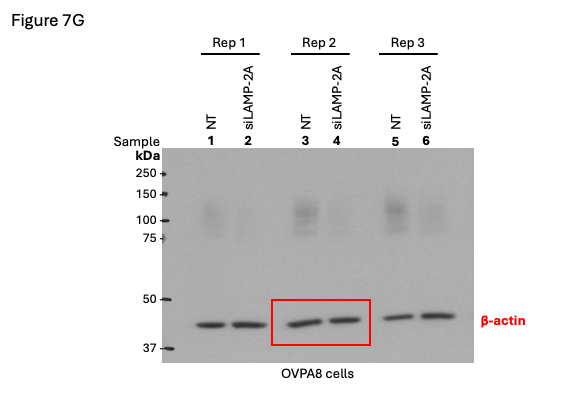

Supplement: Supplementary file 9 — Source data Fig. 7 [file 44321_2025_210_MOESM9_ESM.zip › Figure 7/7G/Figure 7G - b-actin.tiff]

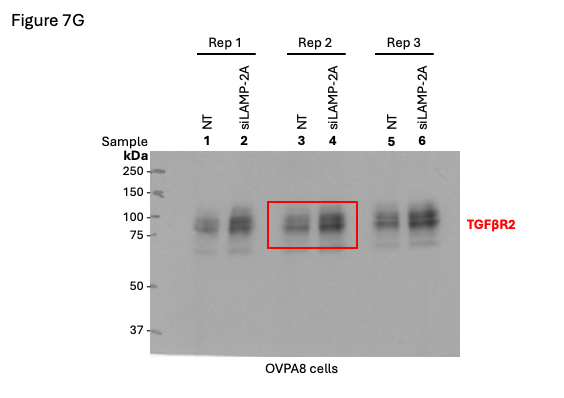

Supplement: Supplementary file 9 — Source data Fig. 7 [file 44321_2025_210_MOESM9_ESM.zip › Figure 7/7G/Figure 7G - TGFBR2.tiff]

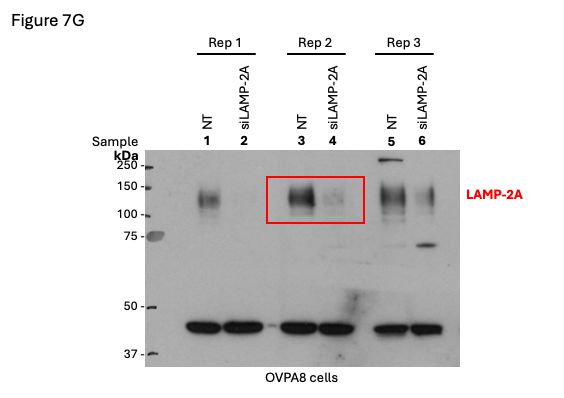

Supplement: Supplementary file 9 — Source data Fig. 7 [file 44321_2025_210_MOESM9_ESM.zip › Figure 7/7G/Figure 7G - LAMP-2A.tiff]

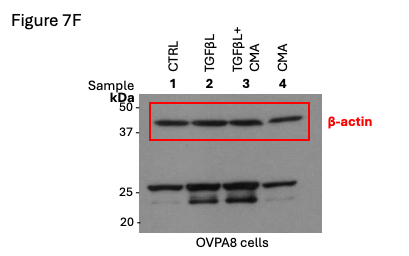

Supplement: Supplementary file 9 — Source data Fig. 7 [file 44321_2025_210_MOESM9_ESM.zip › Figure 7/7F/Figure 7F - b-actin.tiff]

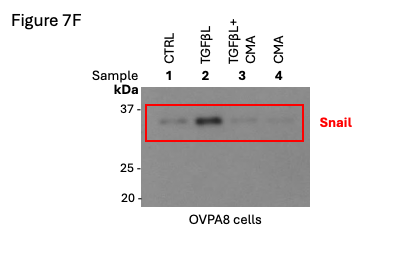

Supplement: Supplementary file 9 — Source data Fig. 7 [file 44321_2025_210_MOESM9_ESM.zip › Figure 7/7F/Figure 7F - Snail.tiff]

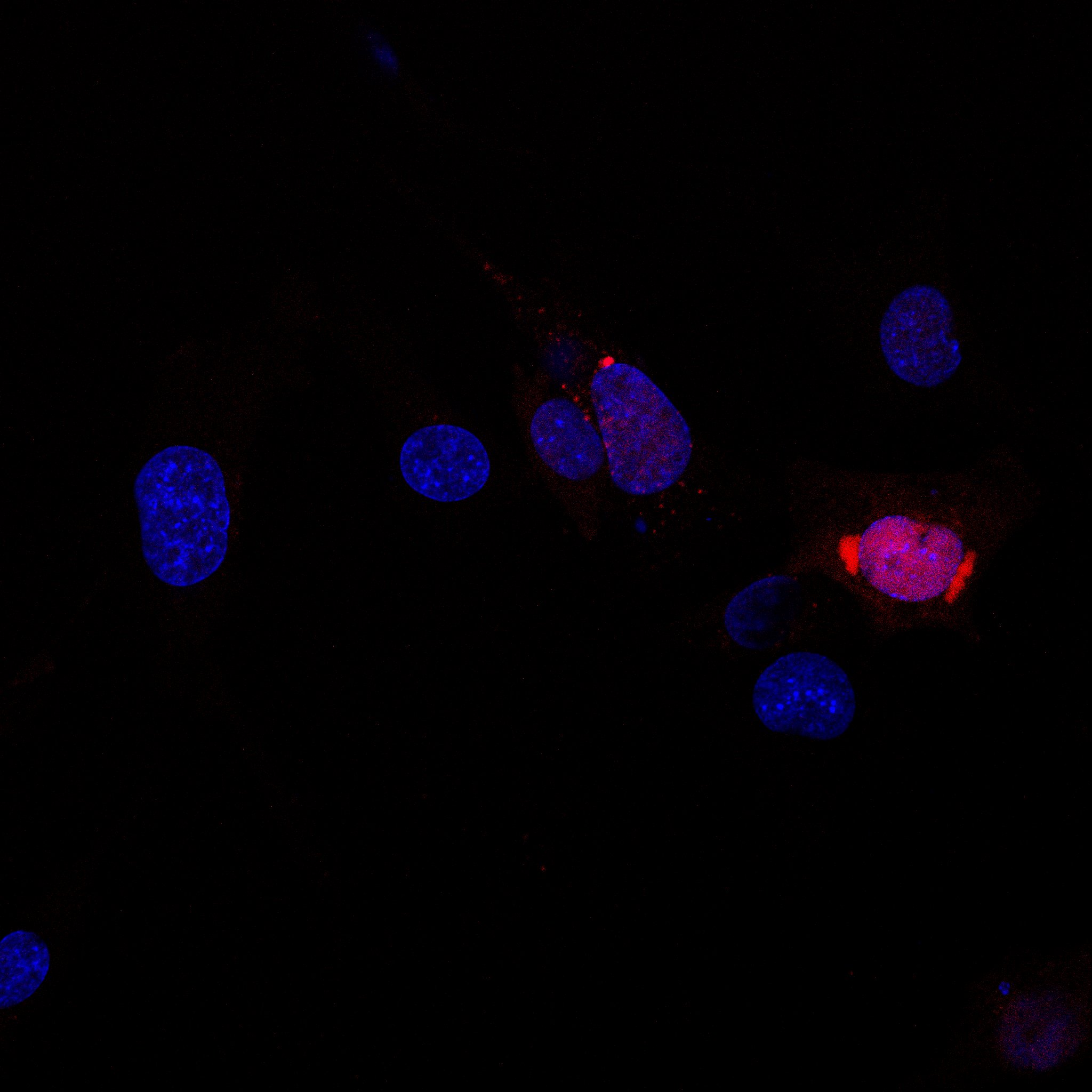

Supplement: Supplementary file 9 — Source data Fig. 7 [file 44321_2025_210_MOESM9_ESM.zip › Figure 7/7A/2. Tranilast_merge.jpg]

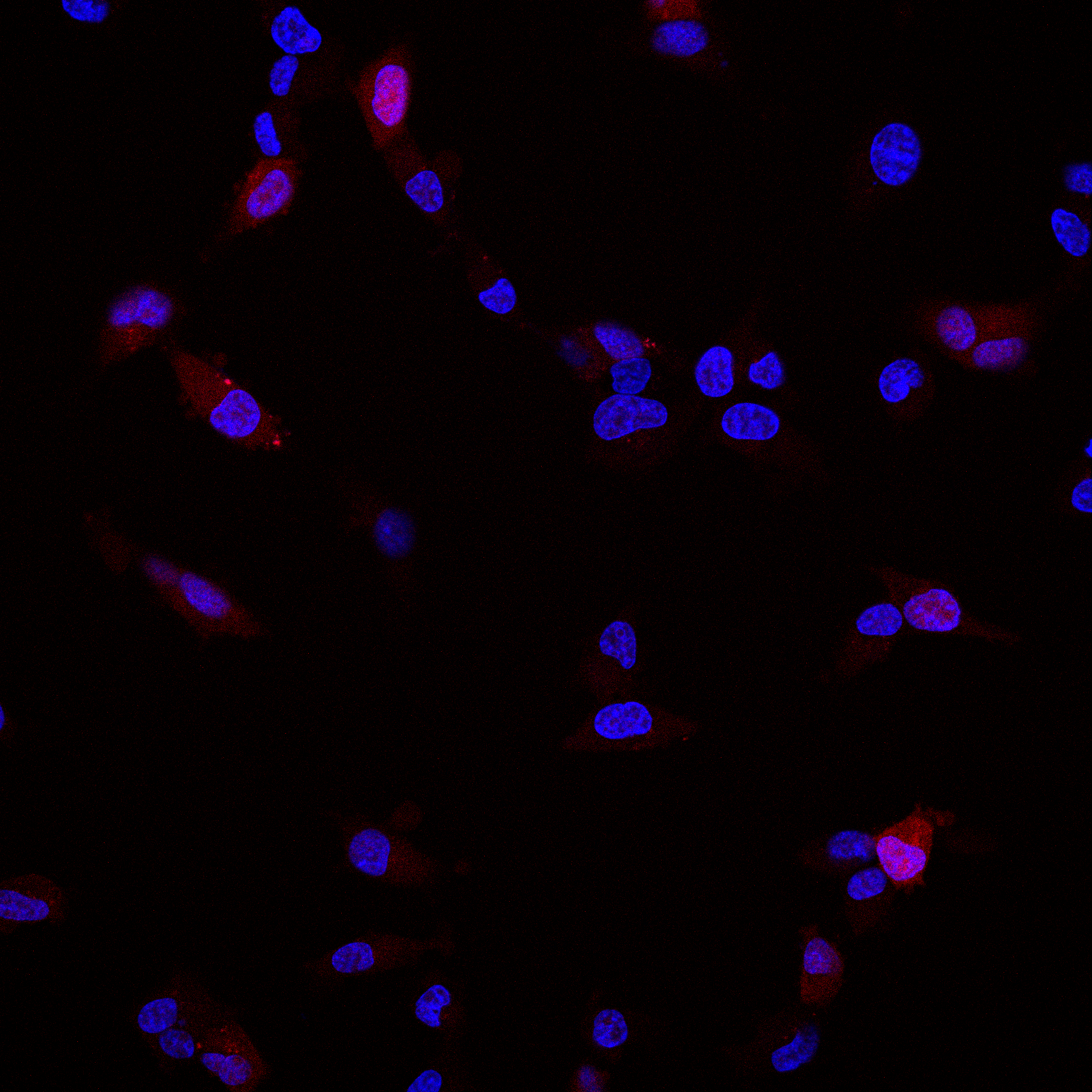

Supplement: Supplementary file 9 — Source data Fig. 7 [file 44321_2025_210_MOESM9_ESM.zip › Figure 7/7A/1. CTRL_merge.tif]

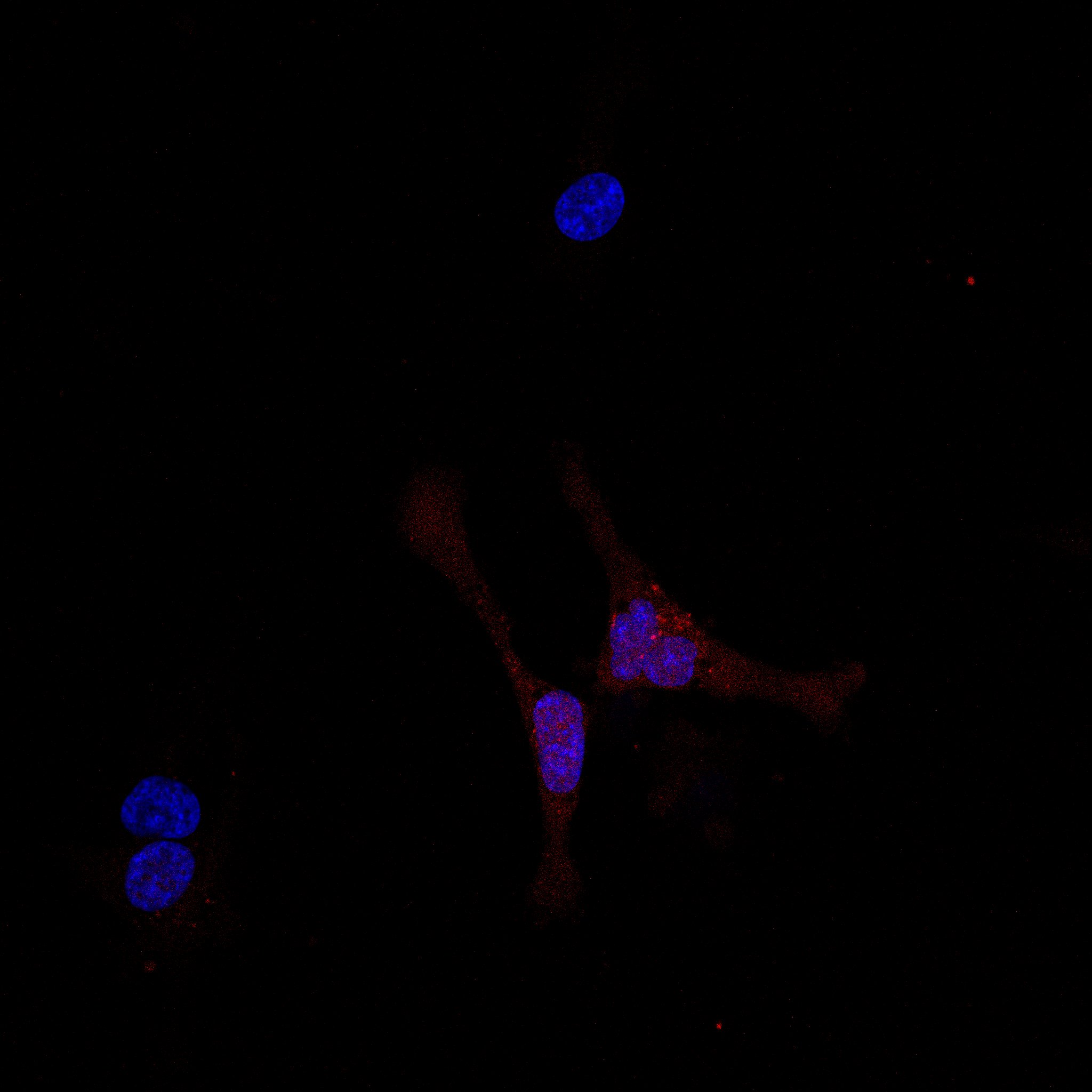

Supplement: Supplementary file 9 — Source data Fig. 7 [file 44321_2025_210_MOESM9_ESM.zip › Figure 7/7A/3. CMA_merge.jpg]

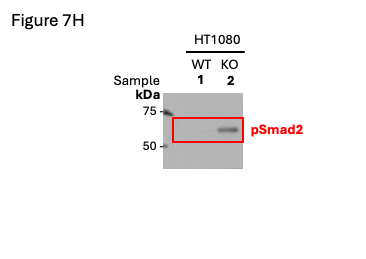

Supplement: Supplementary file 9 — Source data Fig. 7 [file 44321_2025_210_MOESM9_ESM.zip › Figure 7/7H/4. HT1080 pSmad2.tiff]

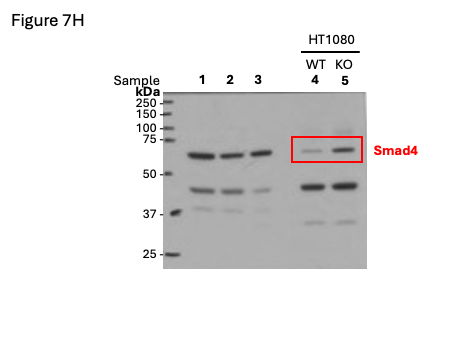

Supplement: Supplementary file 9 — Source data Fig. 7 [file 44321_2025_210_MOESM9_ESM.zip › Figure 7/7H/8. HT1080 Smad4.tiff]

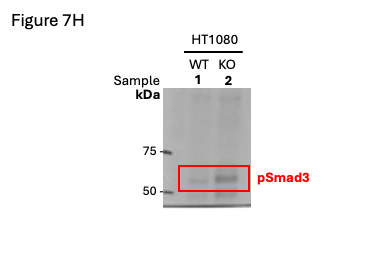

Supplement: Supplementary file 9 — Source data Fig. 7 [file 44321_2025_210_MOESM9_ESM.zip › Figure 7/7H/6. HT1080 pSmad3.tiff]

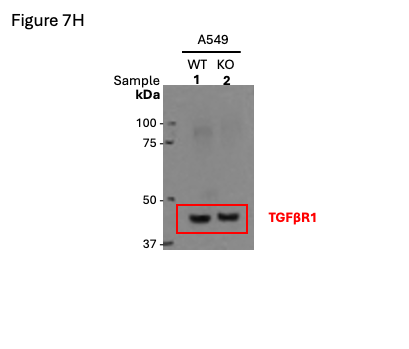

Supplement: Supplementary file 9 — Source data Fig. 7 [file 44321_2025_210_MOESM9_ESM.zip › Figure 7/7H/2. A549 TGFBR1.tiff]
