# Supplementary figures and images for: Chaperone-mediated autophagy regulates the metastatic state of mesenchymal tumors (part 2 of 2)
Source: EMBO Mol Med. 2025 Mar 7;17(4):747–74. doi: 10.1038/s44321-025-00210-w (PMC11982252; doi:10.1038/s44321-025-00210-w)

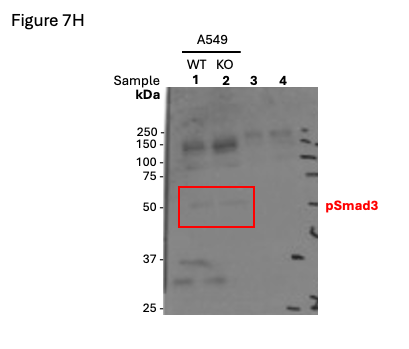

Supplement: Supplementary file 9 — Source data Fig. 7 [file 44321_2025_210_MOESM9_ESM.zip › Figure 7/7H/6. A549 pSmad3.tiff]

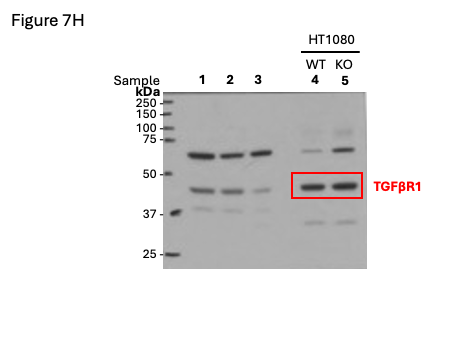

Supplement: Supplementary file 9 — Source data Fig. 7 [file 44321_2025_210_MOESM9_ESM.zip › Figure 7/7H/2. HT1080 TGFBR1.tiff]

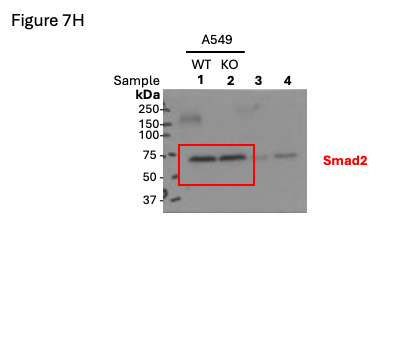

Supplement: Supplementary file 9 — Source data Fig. 7 [file 44321_2025_210_MOESM9_ESM.zip › Figure 7/7H/5. A549 Smad2.tiff]

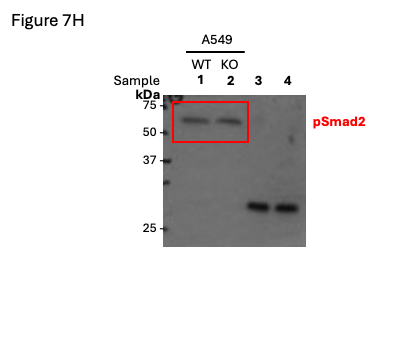

Supplement: Supplementary file 9 — Source data Fig. 7 [file 44321_2025_210_MOESM9_ESM.zip › Figure 7/7H/4. A549 pSmad2.tiff]

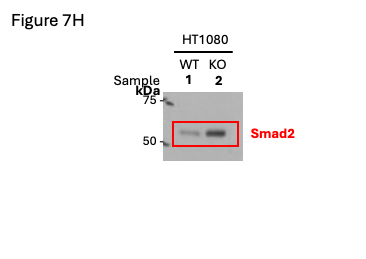

Supplement: Supplementary file 9 — Source data Fig. 7 [file 44321_2025_210_MOESM9_ESM.zip › Figure 7/7H/5. HT1080 Smad2.tiff]

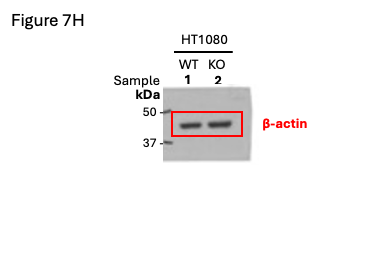

Supplement: Supplementary file 9 — Source data Fig. 7 [file 44321_2025_210_MOESM9_ESM.zip › Figure 7/7H/9. HT1080 b-actin(2).tiff]

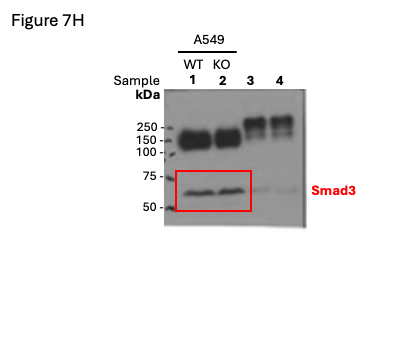

Supplement: Supplementary file 9 — Source data Fig. 7 [file 44321_2025_210_MOESM9_ESM.zip › Figure 7/7H/7. A549 Smad3.tiff]

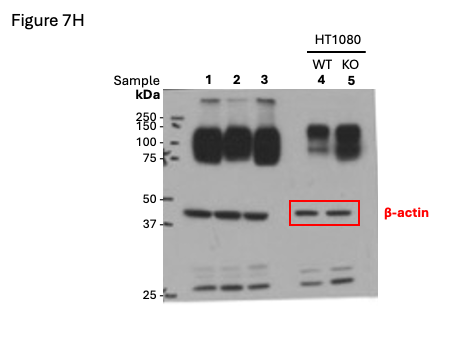

Supplement: Supplementary file 9 — Source data Fig. 7 [file 44321_2025_210_MOESM9_ESM.zip › Figure 7/7H/3. HT1080 b-actin(1).tiff]

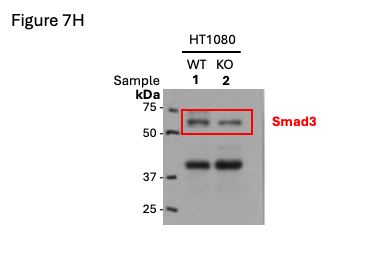

Supplement: Supplementary file 9 — Source data Fig. 7 [file 44321_2025_210_MOESM9_ESM.zip › Figure 7/7H/7. HT1080 Smad3.tiff]

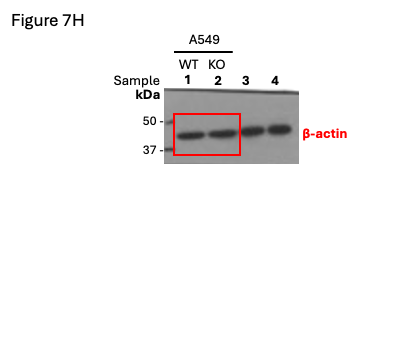

Supplement: Supplementary file 9 — Source data Fig. 7 [file 44321_2025_210_MOESM9_ESM.zip › Figure 7/7H/9. A549b-actin (2).tiff]

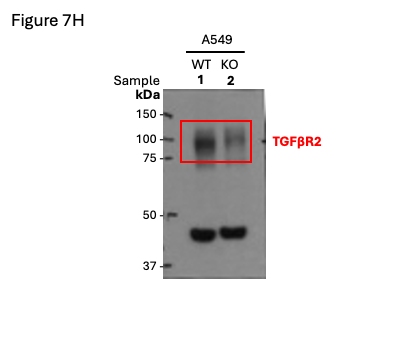

Supplement: Supplementary file 9 — Source data Fig. 7 [file 44321_2025_210_MOESM9_ESM.zip › Figure 7/7H/1. A549 TGFBR2.tiff]

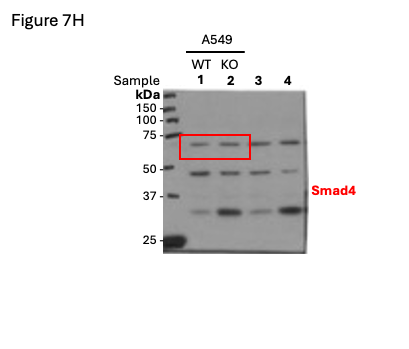

Supplement: Supplementary file 9 — Source data Fig. 7 [file 44321_2025_210_MOESM9_ESM.zip › Figure 7/7H/8. A549 Smad4.tiff]

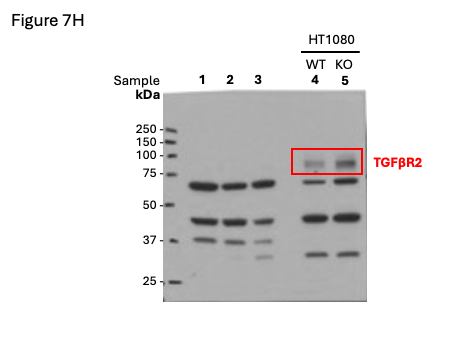

Supplement: Supplementary file 9 — Source data Fig. 7 [file 44321_2025_210_MOESM9_ESM.zip › Figure 7/7H/1. HT1080 TGFBR2.tiff]

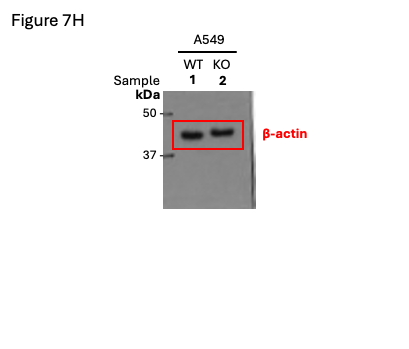

Supplement: Supplementary file 9 — Source data Fig. 7 [file 44321_2025_210_MOESM9_ESM.zip › Figure 7/7H/3. A549 b-actin (1).tiff]

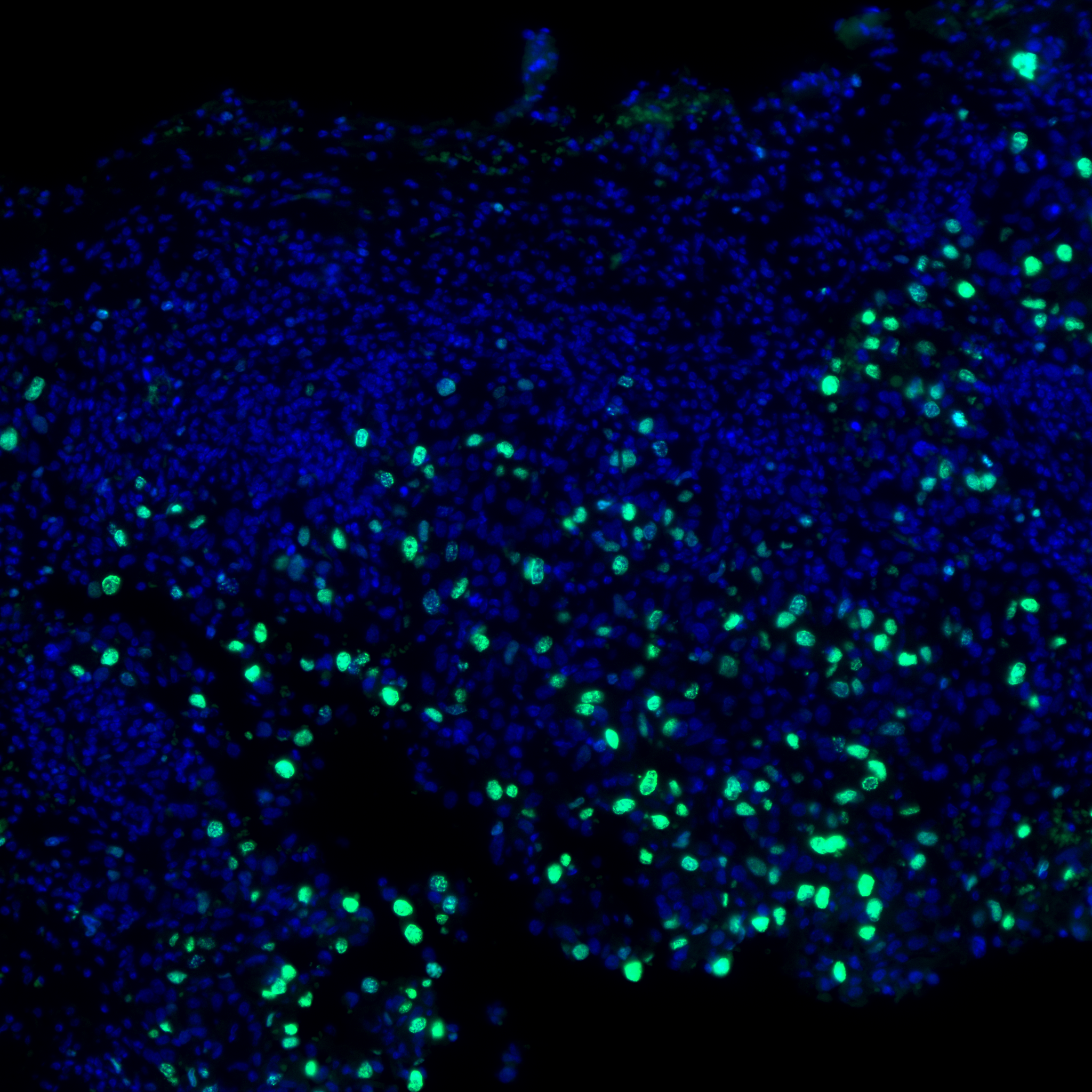

Supplement: Supplementary file 10 — Source data Fig. 8 [file 44321_2025_210_MOESM10_ESM.zip › Figure 8/8G/6. KO 6AN HT1080_EdU.tif]

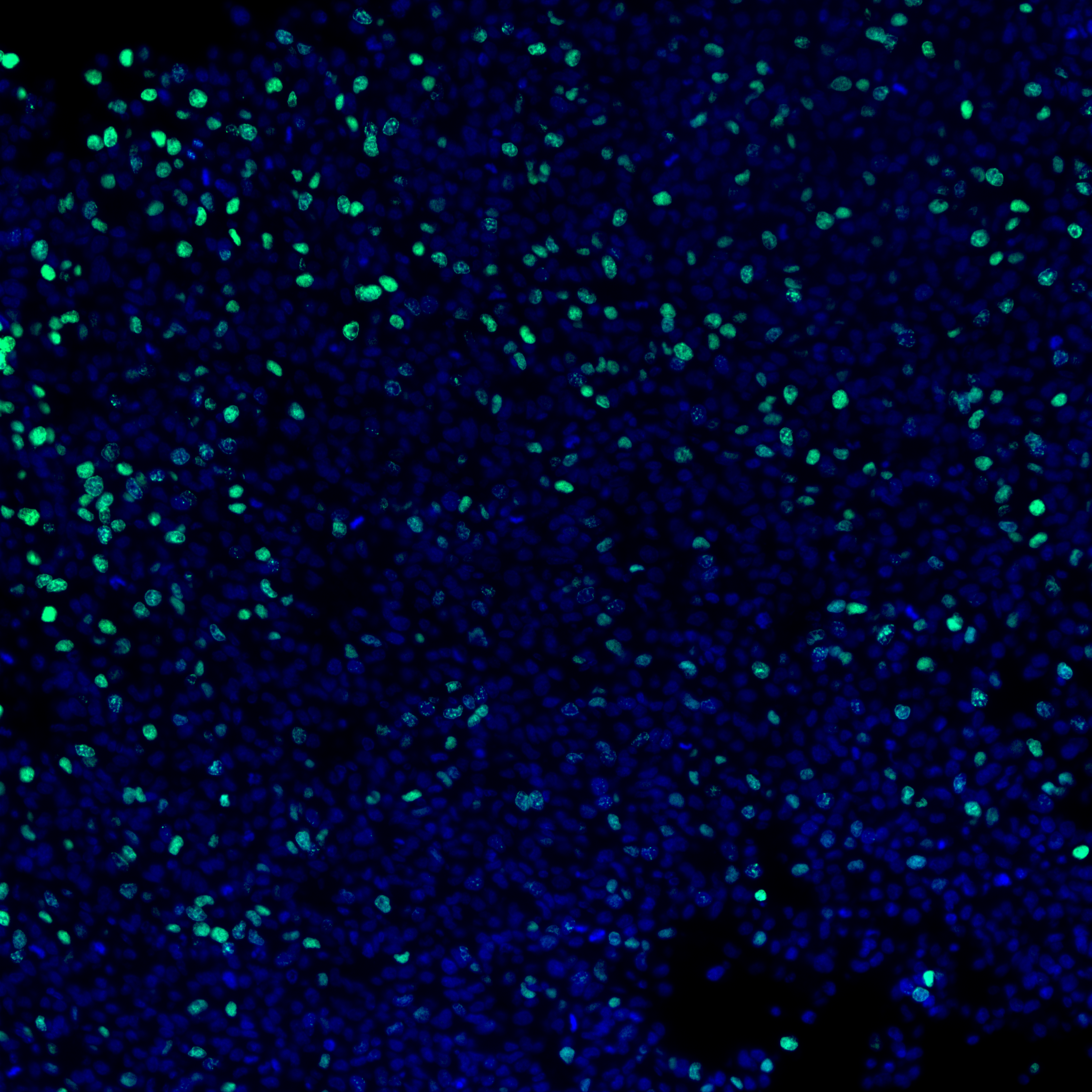

Supplement: Supplementary file 10 — Source data Fig. 8 [file 44321_2025_210_MOESM10_ESM.zip › Figure 8/8G/5. KO TRAN HT1080_EdU.tif]

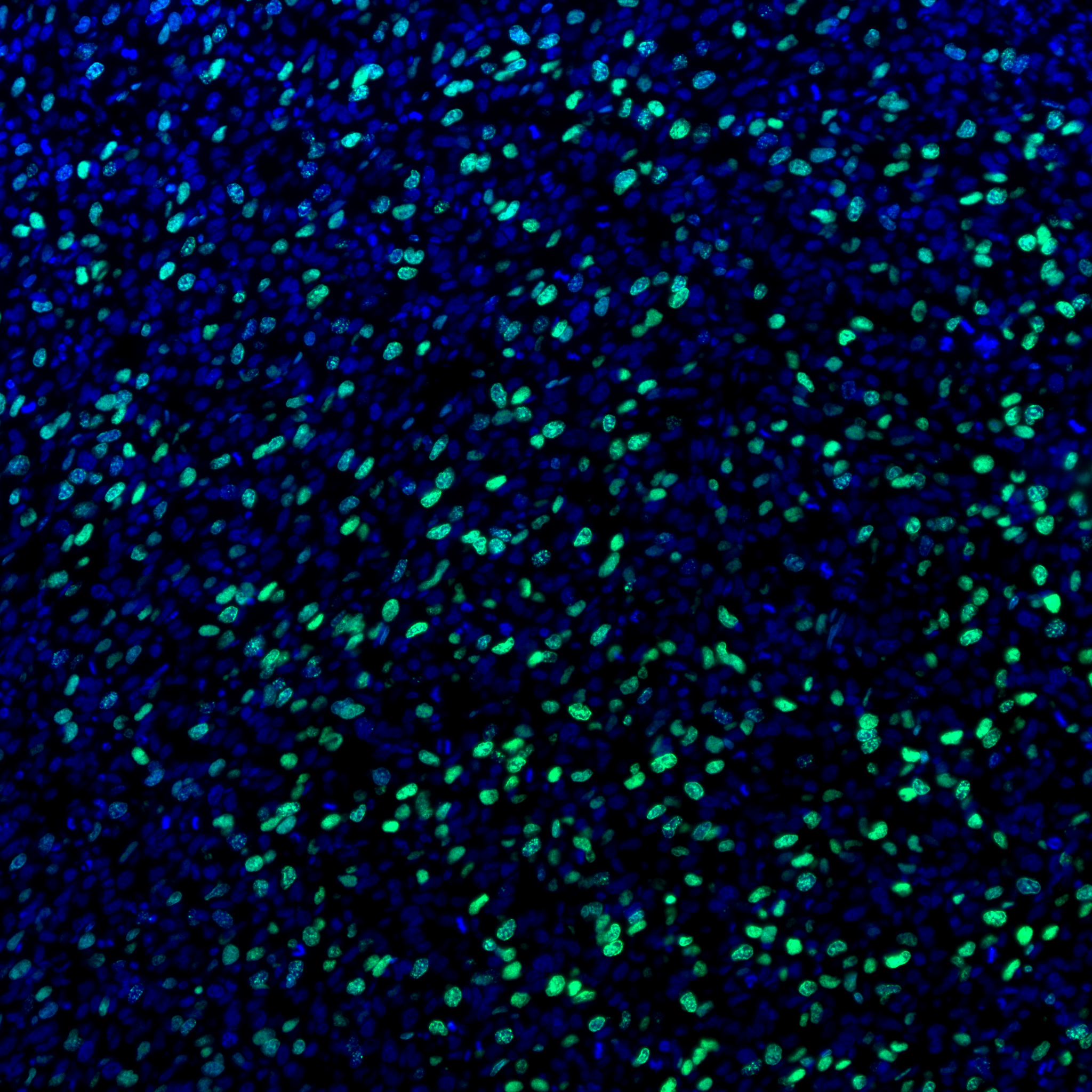

Supplement: Supplementary file 10 — Source data Fig. 8 [file 44321_2025_210_MOESM10_ESM.zip › Figure 8/8G/4. KO Ctrl_HT1080_EdU.tif]

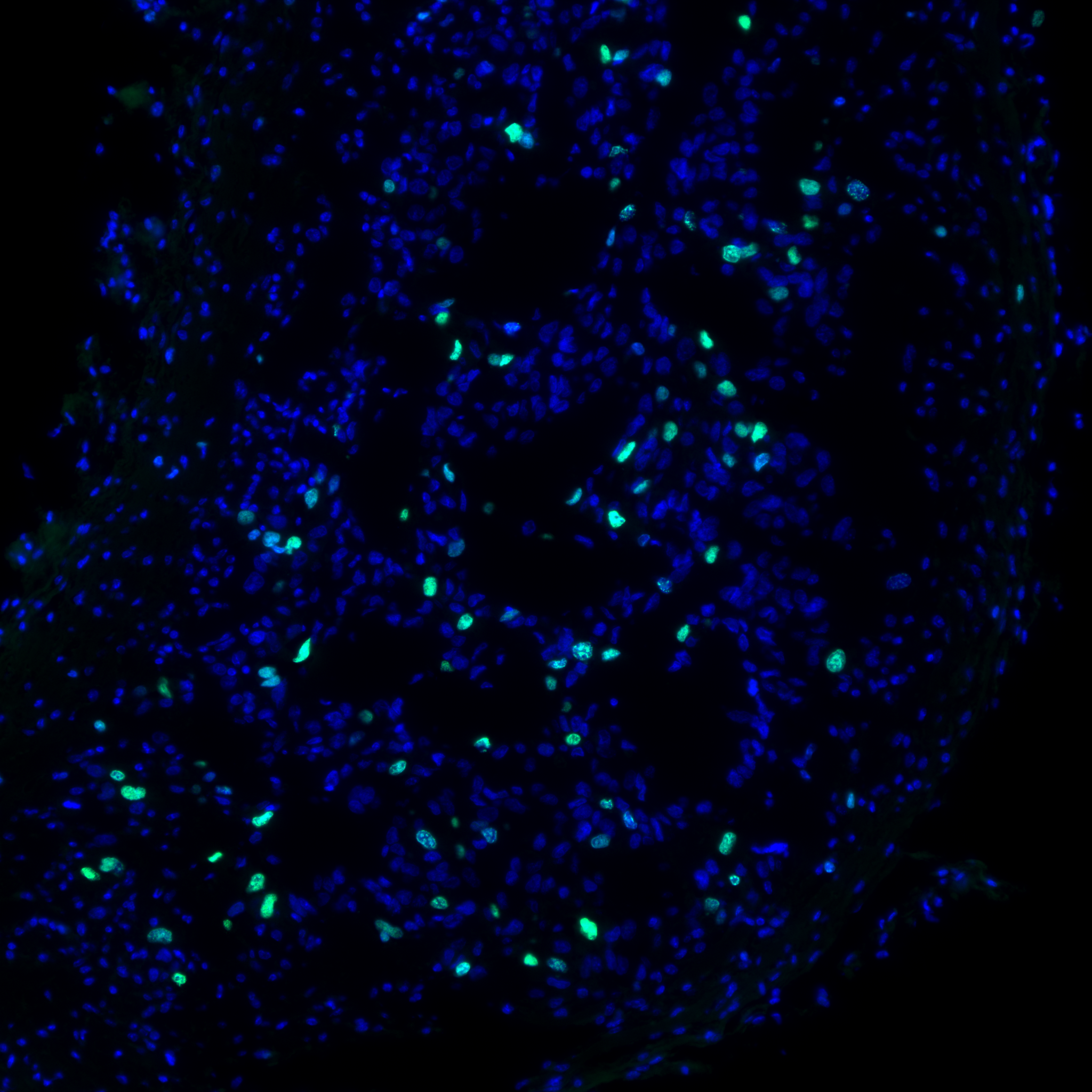

Supplement: Supplementary file 10 — Source data Fig. 8 [file 44321_2025_210_MOESM10_ESM.zip › Figure 8/8G/3. wt 6AN HT1080_EdU.tif]

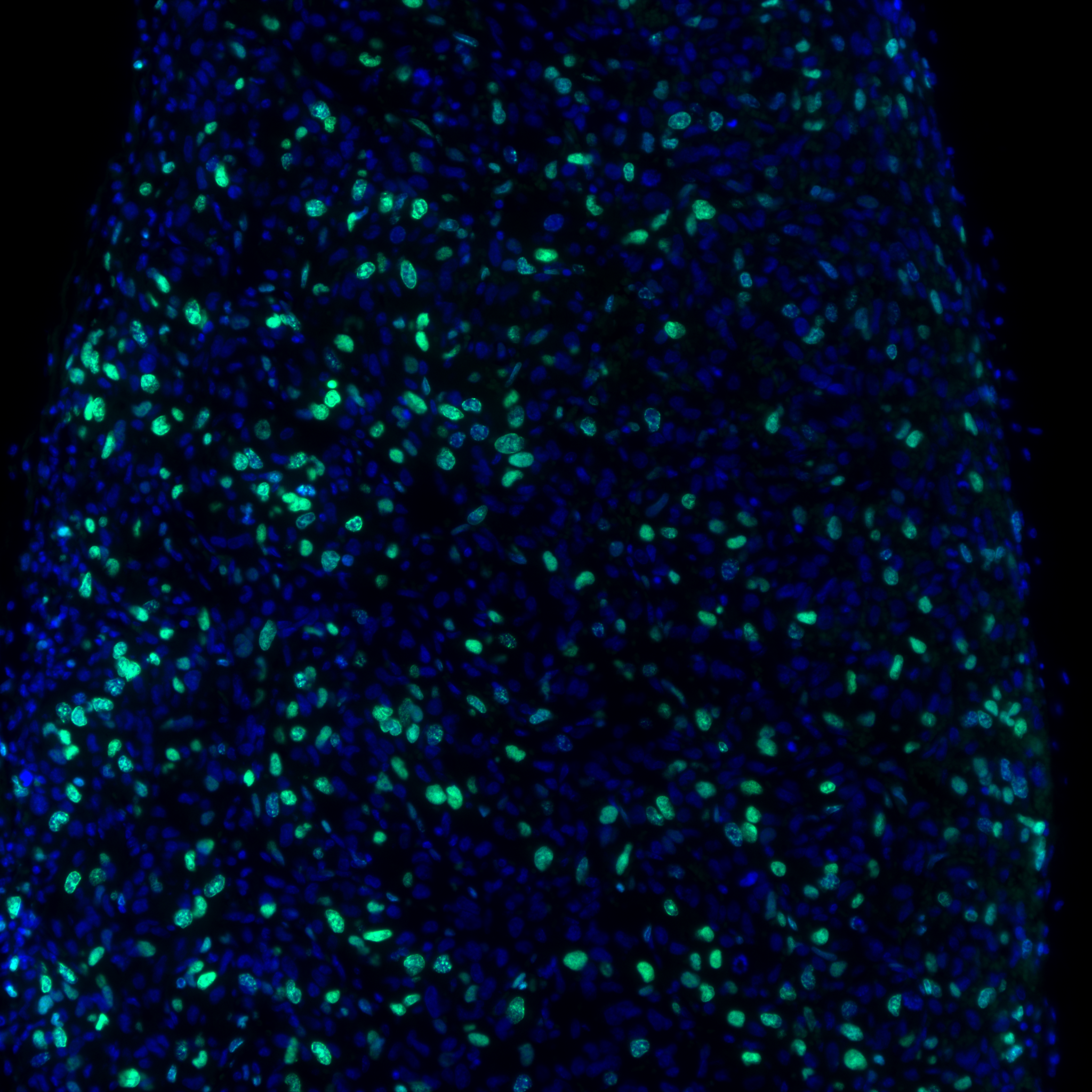

Supplement: Supplementary file 10 — Source data Fig. 8 [file 44321_2025_210_MOESM10_ESM.zip › Figure 8/8G/1. WT Ctrl HT1080_EdU.tif]

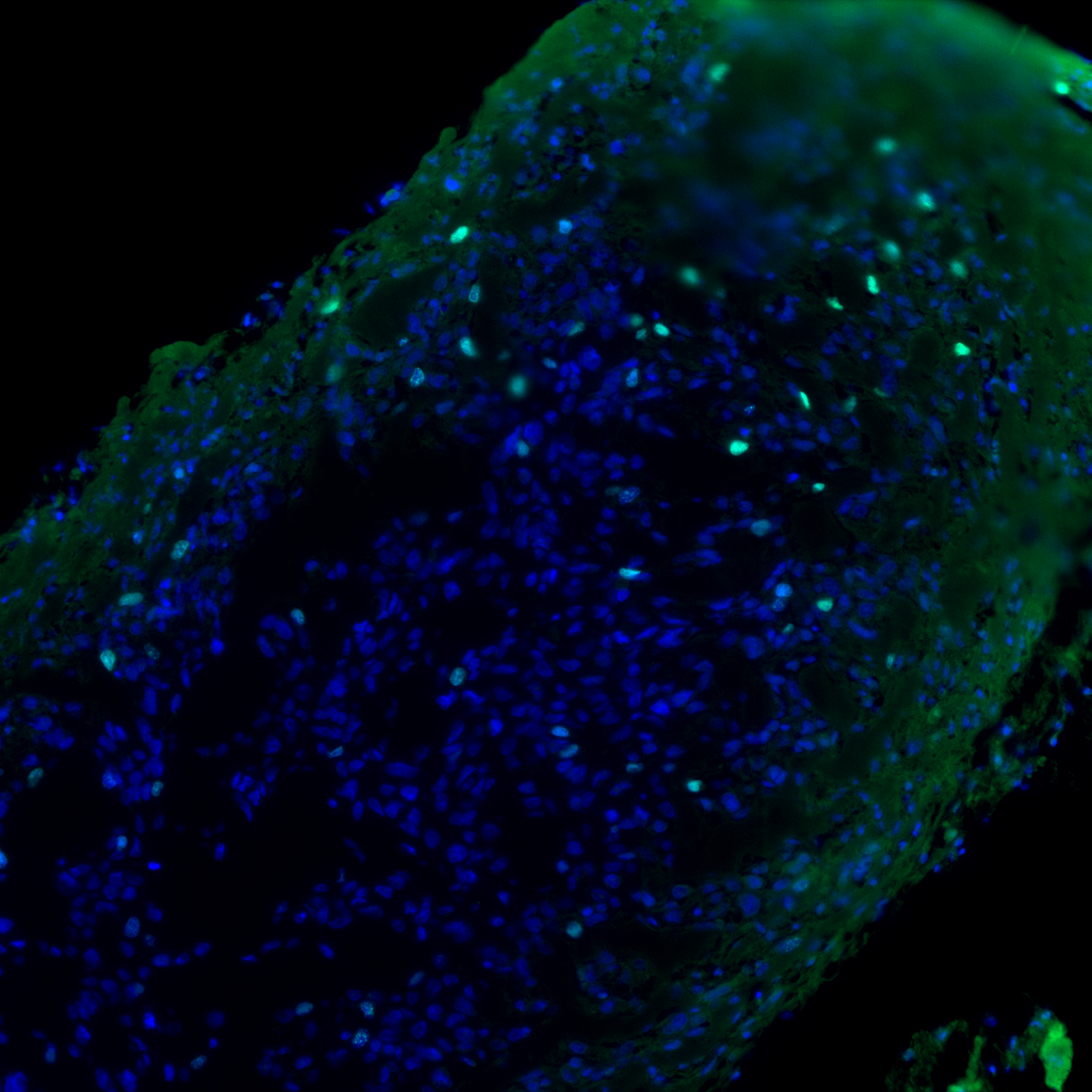

Supplement: Supplementary file 10 — Source data Fig. 8 [file 44321_2025_210_MOESM10_ESM.zip › Figure 8/8G/2. wt_TRAN HT1080_EdU tif.tif]
