# Supplementary material for: Chaperone-mediated autophagy regulates the metastatic state of mesenchymal tumors
Source: EMBO Mol Med. 2025 Mar 7;17(4):747–74. doi: 10.1038/s44321-025-00210-w (PMC11982252; doi:10.1038/s44321-025-00210-w)
Supplement: Supplementary file 12 — Expanded View Figures [file 44321_2025_210_MOESM12_ESM.pdf]

Expanded View Figures

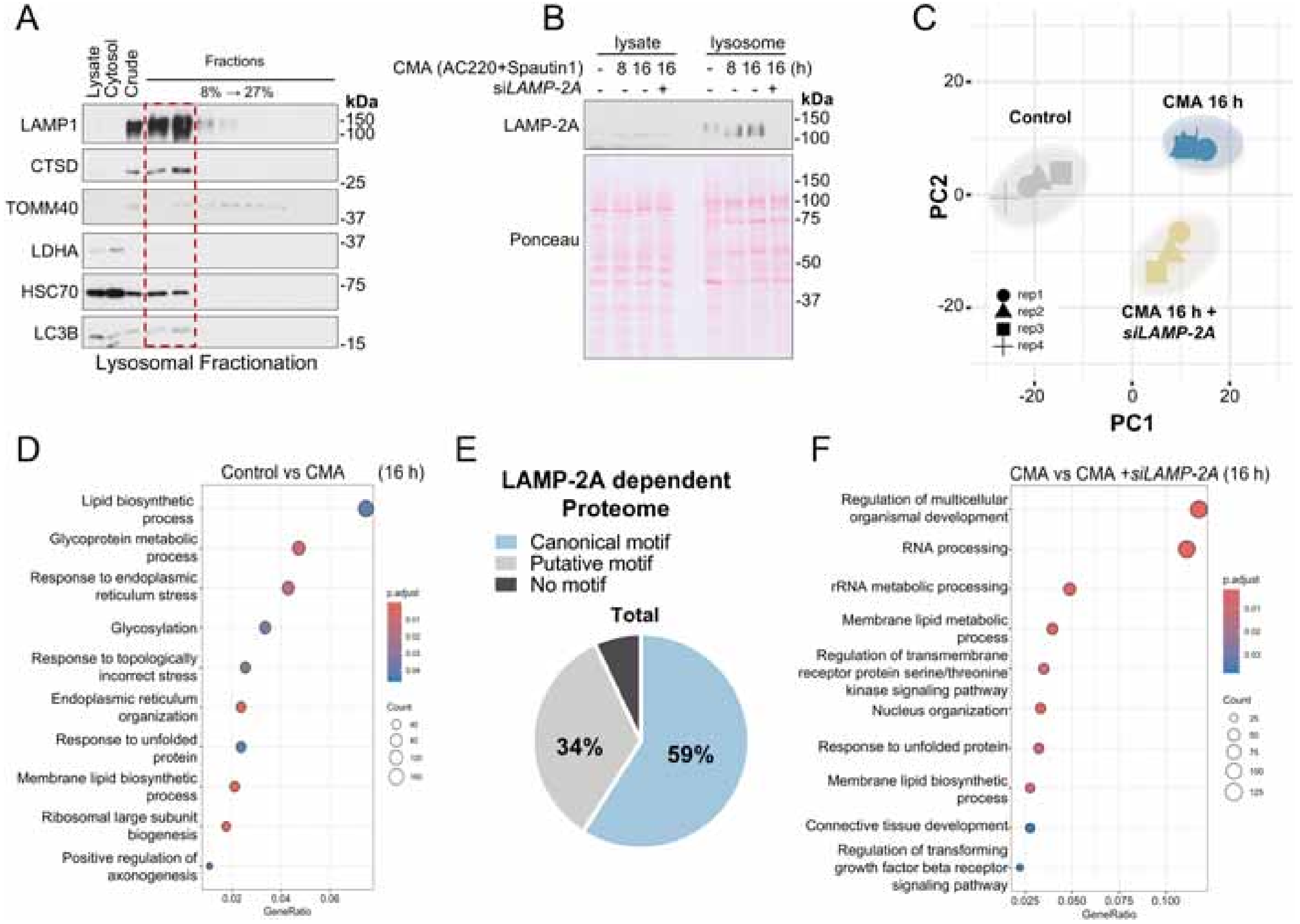

Figure EV1. Lysosomal fractionation and quantitative proteomics of ES2 cells.

(**A**) Cellular fractions isolated from ES2 cells by differential large-scale multi-layered density gradient centrifugations and analyzed by immunoblotting using antibodies against lysosomal membrane protein LAMP1, lysosomal matrix protein cathepsin D (CTSD), mitochondrial protein translocase of outer mitochondrial membrane 40 (TOMM40), cytosolic protein Lactate dehydrogenase A (LDHA), the Hsc70 chaperone protein and macroautophagy marker LC3B. The red square indicates the low percentage gradient fraction, enriched in lysosomes, and chosen for further analysis. (**B**) Cellular lysate and lysosomal fractions from control (DMSO), 8 and 16 h CMA activated (AC220+Spautin1) and 16 h CMA+si*LAMP-2A* conditions analyzed by immunoblotting using LAMP-2A antibody. Total protein loading is visualized by Ponceau S Red staining. (**C**) Score plot of principle component analysis (PCA) in 2D for control, 16 h CMA, and 16 h CMA+si*LAMP-2A* treated sample sets ($n_{exp} = 4$). (**D**) Pathway enrichment analysis by Gene ontology (GO) of biological processes comparing control vs 16 h CMA treated sample sets (Over Representation Analysis). (**E**) Pie graphs of the experimentally validated LAMP-2A-dependent lysosome proteome showing the percentage of proteins with indicated types of KFERQ-like motifs. (**F**) GO analysis comparing 16 h CMA vs CMA+si*LAMP-2A* treated sample sets. Dot plots of top 10 enriched pathways are indicated as the ratio of the differentially expressed gene number to the total gene number for a certain annotation. The size and color of the dots represent the gene ratio and the range of adjusted *P* values, respectively (Over Representation Analysis). Source data are available online for this figure.

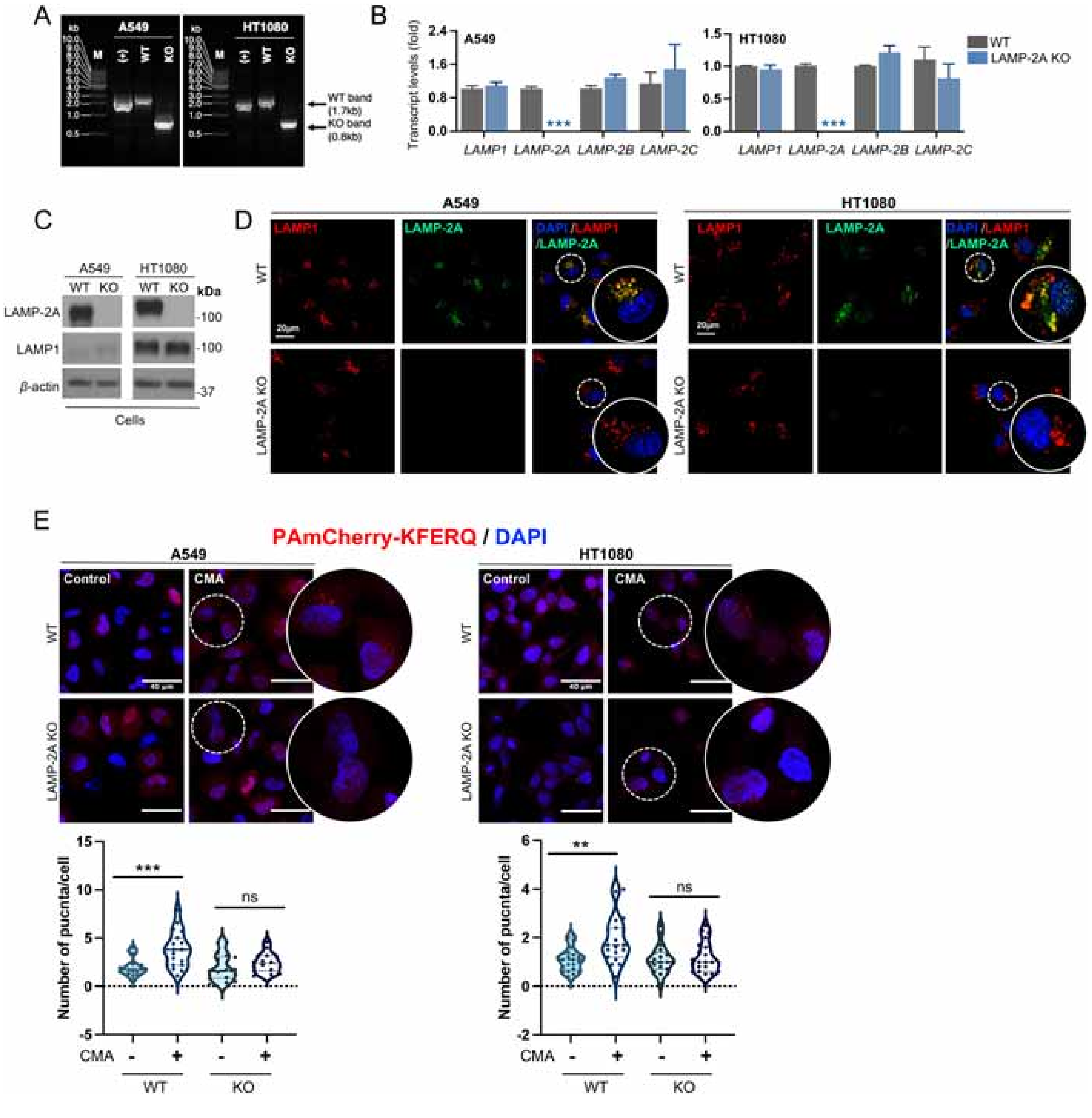

Figure EV2. Generation and validation of isoform-specific knockout of *LAMP-2A*.

(**A**) CRISPR/Cas9 genome editing and genotyping by agarose gel electrophoresis/PCR in regular (+), wild-type (WT), and *LAMP-2A* knockout (KO) A549 and HT1080 cells. Target amplicon in human *LAMP2* gene is detected by the fragment of ~800 bp. M: marker. (**B**) RT–qPCR detection of *LAMP1*, *LAMP-2A*, *-2B*, and *-2C* expression in WT and *LAMP-2A* KO A459 and HT1080 cell lines. Bars represent mean ± sd ($n_{exp} = 3$). *P* values refer to $^{***}P < 0.001$ (A549: $P_{LAMP\text{-}2A} = 0.0001$; HT1080: $P_{LAMP\text{-}2A} < 0.0001$ Student's *t*-test). (**C**) Immunoblot detection of LAMP-2A and LAMP1 in WT and *LAMP-2A* KO A549 and HT1080 cells. *β*-Actin: loading control. (**D**) Representative immunofluorescence confocal images of WT and *LAMP-2A* KO A549 and HT1080 cell lines stained with anti-LAMP-2A (green), anti-LAMP1 (red), and DAPI (blue) for nuclei highlighted with (circular insets) magnification. Scale bars: 20 μm. (**E**) CMA activity measured in WT and *LAMP-2A* KO A549 and HT1080 cells expressing PAmCherry-KFERQ CMA reporter. Cells were treated with AC220+Spautin1 to activate CMA for 16 h. Confocal images (upper panel) of merged red (PAmCherry-KFERQ) and blue (DAPI) for nuclei highlighted with (circular insets) magnification. Scale bars: 40 μm. Quantification (lower panel) of CMA activity as number of fluorescent puncta per cell. For each replicate ($n_{exp} = 3$), at least four images were analyzed. Each dot in the graph represents the average number of puncta per cell. *P* values refer to $^{***}P < 0.001$, ns: non-significant (A549: $P_{WT\ Ctrl\ vs\ CMA} = 0.0008$, $P_{KO\ Ctrl\ vs\ CMA} = 0.28$; HT1080: $P_{WT\ Ctrl\ vs\ CMA} = 0{,}0013$, $P_{KO\ Ctrl\ vs\ CMA} = 0{,}75$; Student's *t*-test). Source data are available online for this figure.

 © The Author(s)

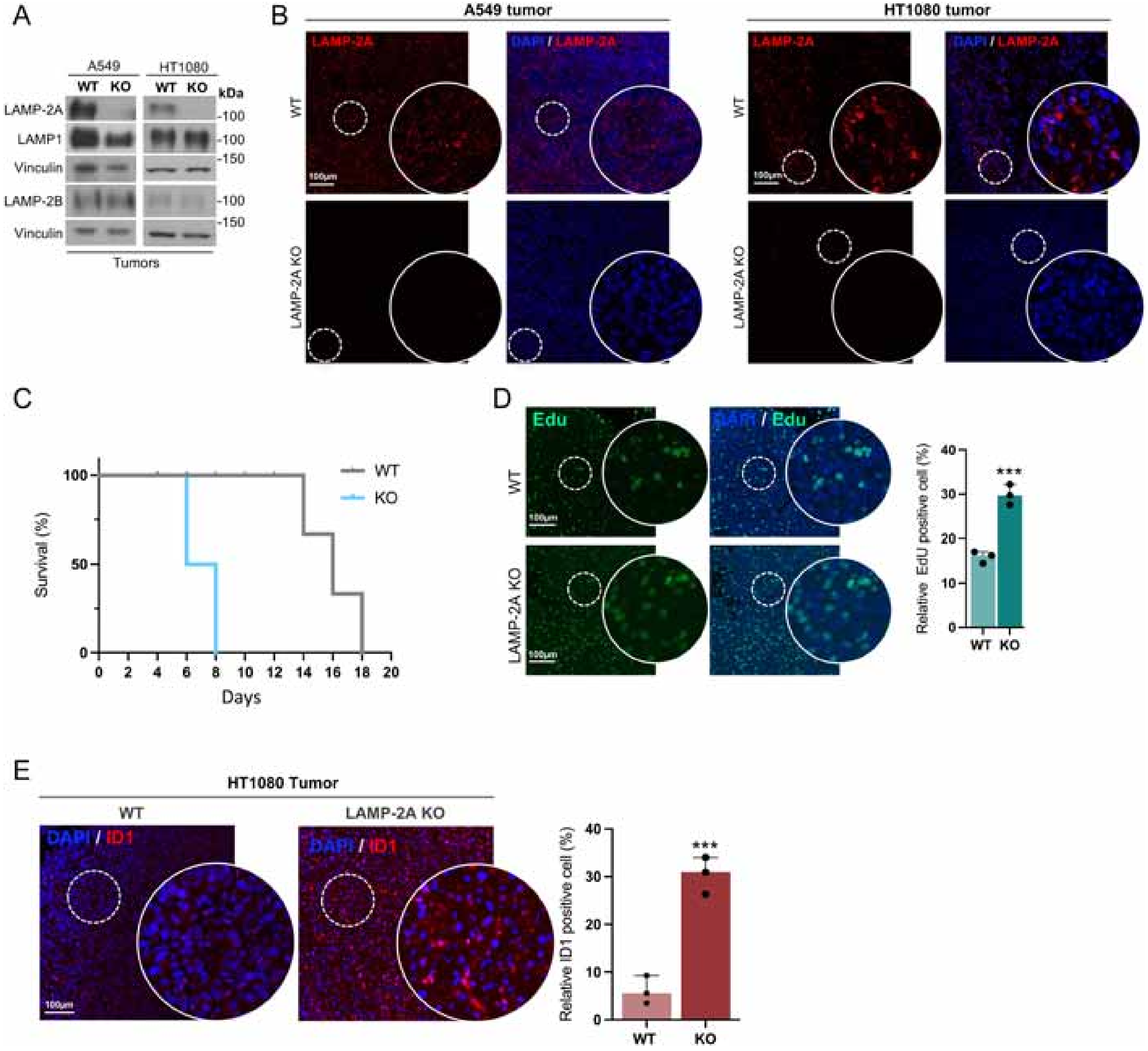

Figure EV3. The impact of *LAMP-2A* knockout in cancer cells and tumors.

(**A**) Immunoblot detection of LAMP-2A, LAMP-2B, and LAMP1 in WT and *LAMP-2A* KO A549 and HT1080 tumor lysates. Vinculin: loading control. (**B**) Immunofluorescence confocal images of WT and *LAMP-2A* KO A549 and HT1080 tumor sections stained with anti-LAMP-2A (red) and DAPI- (blue) for nuclei highlighted with (circular insets) magnification. Scale bars: 100 µm. (**C**) Kaplan–Meier survival curves for mice bearing WT or *LAMP-2A* KO HT1080 tumors. (**D**) Immunofluorescence confocal images (left panel) of EdU positive (green) and DAPI (nuclei, blue) labeled WT and *LAMP-2A* KO HT1080 tumor sections ($n_{exp} = 3$). Scale bars: 100 µm. Quantification (right panel) of the percentage EdU+ cell ratio per 20× field (bar graph). Bars present mean ± sd. *P* values refer to ****P* < 0.001 (*P* = 0.0008; Student's *t*-test). (**E**) Representative immunofluorescence confocal images (left panel) and quantification (right panel) of HT1080 WT and *LAMP-2A* KO tumor section stained with anti-ID1 (red) and DAPI (blue) for nuclei highlighted with (circular insets) magnification. Scale bar: 100 µm. Quantification of the ID1+ cell ratio per 20× field (bar graph) ($n = 3$). Error bars, ±SD. ****P* < 0.001 (*P* = 0.0010; Student's *t*-test). Source data are available online for this figure.

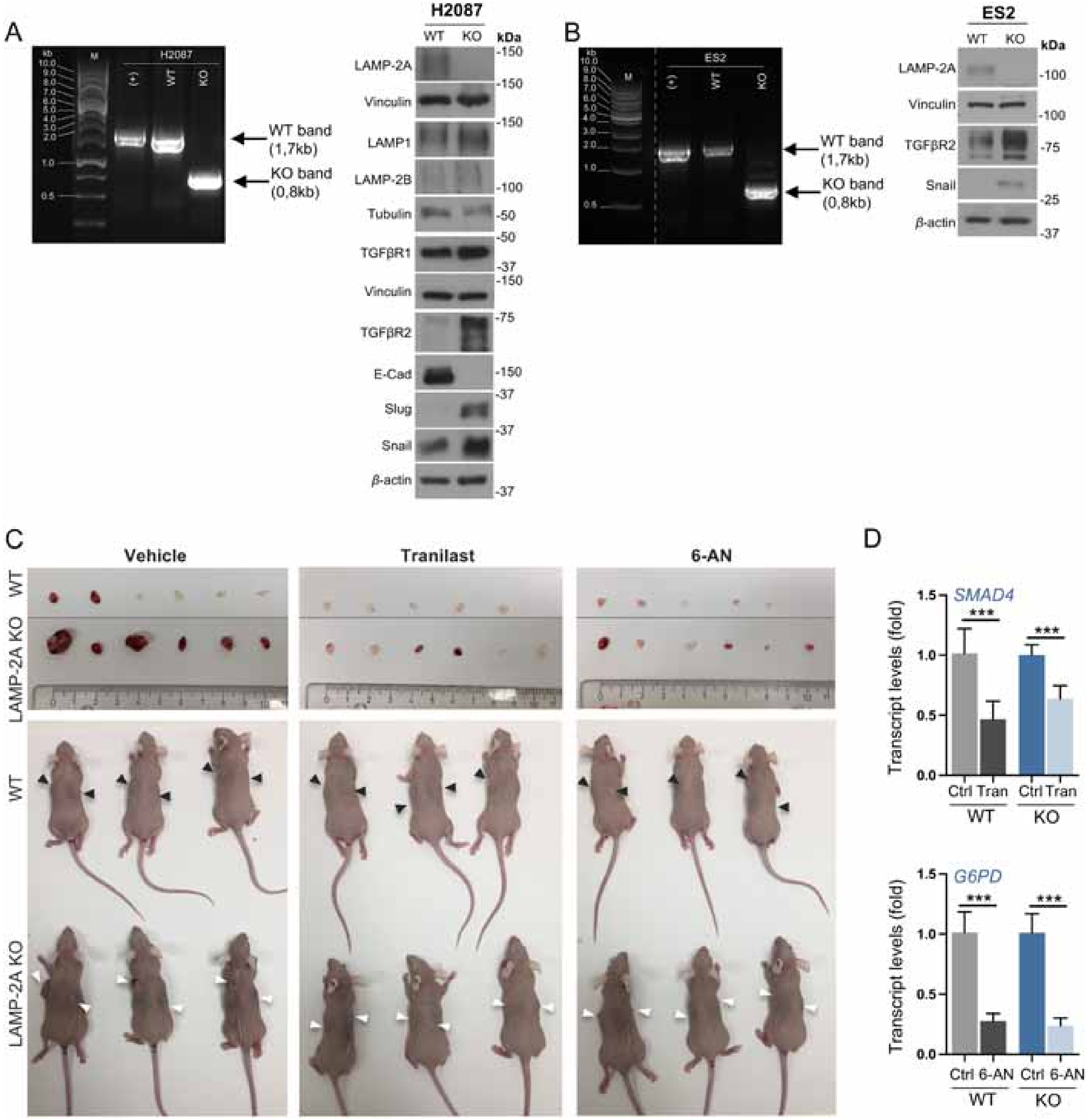

Figure EV4. LAMP-2A-KO tumor regression in vivo.

(**A**) (Left panel) CRISPR/Cas9 genome editing and genotyping by agarose gel electrophoresis/PCR in regular (+), WT, and *LAMP-2A* KO NCI-H2087 cells. Target amplicon in human *LAMP2* gene is detected by the fragment of ~800 bp. M: marker. (Right panel) Immunoblot detection of indicated LAMP proteins, TGFβ receptors, and EMT proteins in WT and *LAMP-2A* KO H2087 cells. Vinculin and tubulin: loading controls. (**B**) (Left panel) CRISPR/Cas9 genome editing and genotyping by agarose gel electrophoresis/PCR in regular (+), WT and *LAMP-2A* KO ES2 cells. Target amplicon in human *LAMP2* gene is detected by the fragment of ~800 bp. M: marker. (Right panel) Immunoblot detection of LAMP-2A, TGFβR2 and Snail in WT and *LAMP-2A* KO ES2 cells. *β*-Actin: loading control. (**C**) Representative images of WT and *LAMP-2A* KO HT1080 xenograft nude mice (two transplants per mice ($n_{mice} = 3$)) and harvested tumors ($n = 6$) after administration with Tranilast or 6-AN for nine days compared to vehicle (corn oil) treated mice/tumors. (**D**) RT–qPCR detection of *SMAD4* or *G6PD* in WT and *LAMP-2A* KO HT1080 tumors after administration with Tranilast (Tran) or 6-AN, respectively, compared to control (Ctrl) vehicle (corn oil) treatment. ($n_{mice} = 3$; Tran: $P_{WT} = 0.0003$, $P_{KO} < 0{,}0001$; 6-AN: $P_{WT} < 0.0001$, $P_{KO} < 0.0001$, Student's *t*-test). Bars present mean ± sd. *P* values refer to ***$P < 0.001$. Source data are available online for this figure.

 © The Author(s)
